# Supplementary material for: STEPFORWARD study: a randomised controlled feasibility trial of a self-aligning prosthetic ankle-foot for older patients with vascular-related amputations
Source: BMJ Open. 2021 Mar 18;11(3):e045195. doi: 10.1136/bmjopen-2020-045195 (PMC7978257; doi:10.1136/bmjopen-2020-045195)
Supplement: Author's manuscript [file bmjopen-2020-045195.draft_revisions.pdf]

BMJ Open is committed to open peer review. As part of this commitment we make the peer review history of every article we publish publicly available.

When an article is published we post the peer reviewers' comments and the authors' responses online. We also post the versions of the paper that were used during peer review. These are the versions that the peer review comments apply to.

The versions of the paper that follow are the versions that were submitted during the peer review process. They are not the versions of record or the final published versions. They should not be cited or distributed as the published version of this manuscript.

BMJ Open is an open access journal and the full, final, typeset and author-corrected version of record of the manuscript is available on our site with no access controls, subscription charges or pay-per-view fees (http://bmjopen.bmj.com).

If you have any questions on BMJ Open's open peer review process please email info.bmjopen@bmj.com

BMJ Open

The STEPFORWARD study: a randomised controlled feasibility trial of a self-aligning prosthetic ankle-foot for older patients with vascular-related amputations

Journal:	*BMJ Open*
Manuscript ID	bmjopen-2020-045195
Article Type:	Original research
Date Submitted by the Author:	26-Sep-2020
Complete List of Authors:	Vanicek, Natalie; University of Hull, Sport, Health and Exercise Science<br>Coleman, Elizabeth; University of York, Health Sciences<br>Watson, Judith; University of York, Health Sciences<br>Bell, Kerry; University of York, Health Sciences<br>McDaid, Catriona; University of York, Health Sciences<br>Barnett, Cleveland; Nottingham Trent University, School of Science and Tecnology<br>Twiste, Martin; University of Salford<br>Jepson, Fergus; Lancashire Teaching Hospitals NHS Foundation Trust, Specialist Mobility Rehabilitation Centre<br>Salawu, Abayomi; Hull University Teaching Hospitals NHS Trust, Disability Medicine and Rehabilitation Unit<br>Harrison, Dennis<br>Mitchell, Natasha; University of York, York Trials Unit, Department of Health Sciences
Keywords:	REHABILITATION MEDICINE, Clinical trials < THERAPEUTICS, Foot & ankle < ORTHOPAEDIC & TRAUMA SURGERY, Musculoskeletal disorders < ORTHOPAEDIC & TRAUMA SURGERY

SCHOLARONE™
Manuscripts

I, the Submitting Author has the right to grant and does grant on behalf of all authors of the Work (as defined in the below author licence), an exclusive licence and/or a non-exclusive licence for contributions from authors who are: i) UK Crown employees; ii) where BMJ has agreed a CC-BY licence shall apply, and/or iii) in accordance with the terms applicable for US Federal Government officers or employees acting as part of their official duties; on a worldwide, perpetual, irrevocable, royalty-free basis to BMJ Publishing Group Ltd ("BMJ") its licensees and where the relevant Journal is co-owned by BMJ to the co-owners of the Journal, to publish the Work in this journal and any other BMJ products and to exploit all rights, as set out in our licence.

The Submitting Author accepts and understands that any supply made under these terms is made by BMJ to the Submitting Author unless you are acting as an employee on behalf of your employer or a postgraduate student of an affiliated institution which is paying any applicable article publishing charge ("APC") for Open Access articles. Where the Submitting Author wishes to make the Work available on an Open Access basis (and intends to pay the relevant APC), the terms of reuse of such Open Access shall be governed by a Creative Commons licence – details of these licences and which Creative Commons licence will apply to this Work are set out in our licence referred to above.

Other than as permitted in any relevant BMJ Author's Self Archiving Policies, I confirm this Work has not been accepted for publication elsewhere, is not being considered for publication elsewhere and does not duplicate material already published. I confirm all authors consent to publication of this Work and authorise the granting of this licence.

For peer review only

Title page:

The STEPFORWARD study: a randomised controlled feasibility trial of a self-aligning prosthetic ankle-foot for older patients with vascular-related amputations

Authors: Natalie Vanicek[1], Elizabeth Coleman[2], Judith Watson[2], Kerry Bell[2], Catriona McDaid[2], Cleveland Barnett[3], Martin Twiste[4], Fergus Jepson[5], Abayomi Salawu[6], Dennis Harrison[7] and Natasha Mitchell[2]

Corresponding author: Professor Natalie Vanicek
Department of Sport, Health and Exercise Science
Faculty of Health Sciences
University of Hull
Hull
HU6 7RX
E: n.vanicek@hull.ac.uk
T: 01482 463607

Author affiliations

[1] Department of Sport, Health and Exercise Science, University of Hull, Hull, UK
n.vanicek@hull.ac.uk Orcid iD 000-0002-9602-3172

[2] York Trials Unit, Department of Health Sciences, University of York, York, UK
izzy.coleman@york.ac.uk Orcid iD 0000-0003-4210-1865
jude.watson@york.ac.uk Orcid iD 0000-0003-0694-3854
kerry.bell@york.ac.uk Orcid iD 0000-0001-5124-138X
catriona.mcdaid@york.ac.uk Orcid iD 0000-0002-3751-7260
natasha.mitchell@york.ac.uk Orcid iD 0000-0002-6904-2976

[3] School of Science and Technology, Nottingham Trent University, Nottingham, UK
cleveland.barnett@ntu.ac.uk Orcid iD 0000-0001-6898-9095

[4] School of Health and Society, University of Salford, Manchester, UK
m.twiste@salford.ac.uk Orcid iD 0000-0003-2724-3283

[5] Specialist Mobility Rehabilitation Centre, Lancashire Teaching Hospitals NHS Foundation Trust, Preston, UK
Fergus.Jepson@lthtr.nhs.uk

[6] Disability Medicine and Rehabilitation Unit, Hull University Teaching Hospitals NHS Trust, Hull, UK
abayomi.salawu@hey.nhs.uk Orcid iD 0000-0003-2496-0826

[7] Public Involvement Member
dharrisonpublic@gmail.com

Keywords

Randomised controlled trial, feasibility, self-aligning, ankle-foot prosthesis, transtibial amputation, acceptability, vascular

Word count: 4,070 words

For peer review only - http://bmjopen.bmj.com/site/about/guidelines.xhtml

ABSTRACT

Objectives: To determine the feasibility of conducting a full-scale randomised controlled trial (RCT) of the effectiveness and cost-effectiveness of a self-aligning prosthetic ankle-foot compared with a standard prosthetic ankle-foot.

Design: Multi-centre parallel group feasibility RCT.

Setting: Five prosthetics centres in England recruiting from July 2018 to August 2019.

Participants: Adults aged ≥ 50 years with a vascular-related or non-traumatic transtibial amputation for one year or longer, categorised as 'limited mobility' and with a non-self-aligning ankle-foot.

Intervention: Participants were randomised into one of two trial arms: self-aligning prosthetic ankle-foot or existing standard prosthetic ankle-foot (non-self-aligning) for a 12-week intervention period.

Outcomes: Feasibility measures: recruitment, consent and retention rates; and completeness of questionnaire and clinical assessment datasets across multiple time points. Feasibility of collecting daily activity data with wearable technology and health resource use data with a bespoke questionnaire. Identification of a primary outcome.

Results: 55 participants were randomised: n=27 self-aligning ankle-foot group, n=28 non-self-aligning ankle-foot group. The consent rate and retention at final follow-up were 86% and 93%, respectively. The average recruitment rate was 1.25 participants/site/month (95% CI 0.39 to 2.1). Completeness of questionnaires ranged from 89-94%, and clinical assessment were 92-95%, including the activity monitor data. The average completion rates for the EQ-5D-5L and bespoke resource use questionnaire were 93% and 63%, respectively.

Conclusions: This feasibility trial recruited and retained participants who were categorised as 'limited mobility' following a transtibial amputation, indicating the trial was acceptable to participants and feasible to deliver as a full-scale RCT. The findings support a future, fully-powered evaluation of the effectiveness and cost-effectiveness of a self-aligning prosthetic ankle-foot compared to a standard non-self-aligning version.

Trial registration

ISRCTN15043643

For peer review only - http://bmjopen.bmj.com/site/about/guidelines.xhtml

ARTICLE SUMMARY

Strengths and limitations of this study

- This was one of few clinical trials in prosthetics services conducted in the UK. The study design is a multi-centre, randomised controlled trial in an under-researched clinical population involving prosthetics.
- The completeness of subjective and objective assessment measures, administered in a clinical setting and via post, was high.
- Feasibility and acceptability were ascertained with quantitative and trial procedure data.
- Recruitment was lower and slower than anticipated, but the ability to retain participants was high.
- Effectiveness and cost-effectiveness of a self-aligning ankle-foot will need to be established in a definitive full-scale trial.

For peer review only

For peer review only - http://bmjopen.bmj.com/site/about/guidelines.xhtml

INTRODUCTION

In the UK, there are approximately 6,000 new referrals to prosthetics services every year, most commonly at the transtibial (below-knee) level and in patients aged over 50 years.[1] Major lower limb amputations result from long-term diabetes mellitus, coronary heart, cerebrovascular and peripheral vascular diseases.[2, 3] Consequently, most new referrals for a prosthesis involve older adults who have other health comorbidities and who are categorised as 'limited mobility'. These patients are an under-researched group, with few studies focused on their mobility with a prosthesis.

There are many factors that determine the type of prosthesis that a patient receives, including estimation of patient outcomes, patient goals, and budget.[4] A Cochrane Review reported there was insufficient evidence from robust studies to support the "overall superiority of any individual type of prosthetic ankle-foot mechanism".[5] There are also no standardised criteria for the prescription of ankle-foot prostheses. Practice varies across UK centres and is frequently cost-driven. Subsequently, the majority of older people with a transtibial amputation receive a standard, non-self-aligning ankle-foot, such as the non-articulated solid ankle cushioned heel (SACH), uniaxial or multiaxial prosthetic foot. These prostheses cannot align to sloped surfaces commonly found in daily environments, making walking more difficult and tiring. Our public involvement members reported that a poorly functioning prosthesis can contribute to sedentary behaviour, pain and more frequent visits to prosthetics centres and other healthcare services, which may lead to disuse and poorer quality-of-life. Therefore, identifying a more functionally suitable prosthesis could benefit patients in meaningful ways following amputation.

A self-aligning prosthetic ankle-foot has been designed to attenuate many of the burdens associated with a standard, non-self-aligning ankle-foot and specifically for users categorised as 'limited mobility' (K2 users).[6] This prosthesis uses a hydraulic mechanism to self-align to sloped surfaces (due to ground reaction forces) and remains in a dorsiflexed position throughout the swing phase, thereby improving ground clearance and securing the biological knee, which is important for falls prevention.[7] Previous laboratory-based studies reported that a self-aligning ankle-foot reduced residuum-socket interface pressures in active users,[8] which could alleviate pain in the residuum longer-term, improved walking[9] and enhanced quality of life[10, 11] in users with lower activity levels. More functional prostheses may improve patient function and mobility following a lower limb amputation.[12] Although a self-aligning ankle-foot is more expensive than a non-self-aligning version (by approximately £900 at 2020 prices, although this varies somewhat across manufacturers), the potential patient benefits, such as improved mobility, better quality-of-life, and reduced falls, could offset future healthcare and socioeconomic costs. Robust study designs are needed, particularly involving patients that represent the majority of people with a transtibial amputation and their health comorbidities, to ascertain the effectiveness and cost-effectiveness of a self-aligning prosthetic ankle-foot.

For peer review only - http://bmjopen.bmj.com/site/about/guidelines.xhtml

The STEPFORWARD study used a mixed-methods approach to determine feasibility and acceptability of the study procedures to assess the possibility of conducting a large-scale randomised controlled trial (RCT) of the effectiveness and cost-effectiveness of a self-aligning prosthetic ankle-foot for older patients, with a vascular-related or non-traumatic transtibial amputation, compared to a standard, non-self-aligning ankle-foot. This article reports the feasibility as determined by the quantitative data, which was assessed according to: (1) participant recruitment, including time to recruit, consent and retention rates and adverse events; (2) completeness of data across multiple time points, including health economics data while piloting a bespoke health resource use questionnaire; and (3) day-to-day activity measured with wearable technology.

METHODS

Study design and participants

The STEPFORWARD trial was a multi-centre, parallel group, randomised controlled feasibility trial to assess the possibility of conducting a full-scale RCT. The trial received favourable ethical approval from the National Health Service Yorkshire and the Humber—Leeds West Research Ethics Committee (ref:18/YH/0089) and Health Research Authority in May 2018. The trial was registered on the ISRCTN clinical trial registry (ISRCTN15043643). The full study protocol, with detailed inclusion/exclusion criteria and recruitment pathways, has been published[13] and is briefly described below.

We aimed to recruit 90 participants who were aged ≥ 50 years with a unilateral transtibial amputation for ≥1 year due to vascular, neurological or life-limiting reasons, who were categorised as 'limited mobility', with a stable residuum, and using a non-self-aligning prosthetic ankle-foot (e.g., SACH, uniaxial, multiaxial (e.g., Multiflex) or other K1/K2 feet). Allowing for 20% attrition, we anticipated 72 participants in our final analysis.

Recruitment and randomisation

Potential participants were identified by database screening or during routine appointments to prosthetics centres. Those who agreed to be contacted were first screened over the telephone. If they were potentially eligible, they were invited into clinic for a face-to-face screening and to complete the outstanding screening questions. Following consent, eligible patients completed all baseline measures before being randomised into one of two trial arms: keep their non-self-aligning prosthetic ankle-foot (standard treatment group) or receive a new self-aligning ankle-foot (intervention group). Participants were randomised individually, stratified according to prosthetics centre on a 1:1 basis, by a telephone randomisation service set up by York Trials Unit. Blinding of the participants and investigators was not possible because of the nature of the intervention.

For peer review only - http://bmjopen.bmj.com/site/about/guidelines.xhtml

Standard treatment and intervention

Participants in the standard treatment group continued using their normal prosthetic ankle-foot and had access to all clinical services as normal. They were asked to carry on with their normal daily routine during the intervention period.

Participants in the intervention group were fitted with the self-aligning prosthetic ankle-foot by their regular prosthetist. The self-aligning ankle-foot used in this trial was the Avalon^{K2}, manufactured by Blatchford, Basingstoke, UK (Patent reg: 5336386), which was already commercially available and could be prescribed under the national health service (NHS). Once fitted, participants were asked to ambulate with it as they would normally with any new prosthetic ankle-foot for approximately 12 weeks after fitting (intervention period). At the end of the study, participants in the intervention group only could keep the self-aligning ankle-foot if they preferred it. Participants in standard treatment group were not offered the self-aligning ankle-foot at the end of the study.

Outcome measures

The primary outcome related to feasibility data on recruitment, consent, and retention rates. Reasons for non-participation or withdrawal were recorded where possible. We assessed the completeness of data across multiple time points: baseline, interim (week 9) and final (week 15) follow-ups. The baseline and final assessments were conducted at the participant's usual prosthetics centre. At the interim follow-up, only questionnaire data were collected via post. Data were gathered from questionnaires (baseline demographics and bespoke health resource use questionnaires, the Locomotor Capabilities Index (LCI-5),[(14)] Houghton Scale,[(15)] the Patient-Reported Outcomes Measurement Information System (PROMIS) Short-Form v1.0 Pain (3a and 8a) questionnaires[(16)] and EQ-5D-5L[(17)]), clinical assessments (Timed Up and Go (TUG) test,[(18)] Timed Up and Down Stairs (TUDS) test,[(19)] 2-minute walk test (2mWT)[(20)] and Berg Balance Scale (BBS)[(21)]) and from a loaned activity monitor (activPAL4™, PAL Technologies Ltd, Glasgow, UK) worn on the prosthesis for one week, which collected information related to daily step count and time spent walking/sitting/standing. At baseline, all participants wore their normal (non-self-aligning) prosthetic ankle-foot during the clinical assessments and activity monitor data collection; at final follow-up they wore whichever prosthetic ankle-foot they were using currently. We consulted with members of our Patient Advisory Group to identify a suitable primary outcome to inform our sample size calculation for a future full-scale RCT. Adverse events (AEs) related to the prosthesis only, and any serious adverse events (SAEs), were recorded throughout the study.

Statistical analyses

The recruitment rate was reported monthly, and overall, by site. An average monthly recruitment rate was calculated, with a 95% confidence interval (CI) estimated from the

For peer review only - http://bmjopen.bmj.com/site/about/guidelines.xhtml

data collected. The number of eligible patients and those approached for consent was summarised overall, by site, using counts and percentages.

Baseline data were summarised by trial arm, as randomised, with no formal comparison between the groups. Continuous data were reported descriptively and categorical data by counts and percentages. Completion rates of all the clinical outcome measures were reported by trial arm and overall.

Health economic analysis

A full cost-effectiveness analysis was not undertaken as this was a feasibility study. Nonetheless, the costing approach was undertaken from an NHS perspective; unit costs were derived from established national costing sources such as NHS Reference Costs[22] and Personal Social Services Research Unit costs of health and social care.[23]

RESULTS

Feasibility assessment

Recruitment

Over 880 potential participants were initially screened by searching patient databases across five prosthetics centres in England between July 2018 and August 2019. Eighty-six percent of those screened, who were found to be fully eligible, consented to participating (55 of 64 participants) in the trial. The denominator was 64 as two were randomised in error and subsequently withdrawn (Figure 1). As we used a two-stage screening process, only potential participants who met the initial inclusion criteria were approached; those uninterested in being involved during the initial screening were not screened further. Therefore, the consent rate may be artificially high. Reasons for non-participation are also presented in Figure 1. In total, 55 participants (61% of the target) were included and randomised as follows: 27 to the self-aligning prosthetic ankle-foot (intervention) group and 28 to the non-self-aligning (standard treatment) group.

Initial recruitment was slower than anticipated, therefore two additional recruiting sites were included midway through the trial, bringing the total number of participating sites to five. The average recruitment rate was 1.25 participants/site/month (95% CI 0.39 to 2.1) ranging from 0.83 to 2.2 participants per month. Each site recruited approximately the same number of participants (mean=11, range=9-13 participants), despite them being open to recruitment for different lengths of time (range=5.0-13.7 months).

Participants

The mean (SD) age of participants in this study was 68.8 (9.6) years, ranging from 51.8 to 86.8 years. Most participants were male (85.5%) and all classified themselves as White British, Irish or other. The most common reason for amputation was diabetes (45.5%). Full participant characteristics are described in Table 1.

For peer review only - http://bmjopen.bmj.com/site/about/guidelines.xhtml

Follow-up and adverse events

In addition to the two people randomised in error and withdrawn, a further two participants who were allocated to the intervention group withdrew from the study: one as a result of having suffered a stroke and one reported the self-aligning prosthetic ankle-foot was too heavy. At final follow-up, 51 participants returned the questionnaire (71% of the expected number of participants included in the final analysis), giving a retention rate of 93%.

A total of four SAEs and one AE were reported. The SAEs were due to the following reasons: stroke; fall and femoral fracture when not wearing their prosthesis (both of these cases occurred in the intervention group); fall and hip fracture, leading to hospitalisation and subsequently death; and recurrent falls with fibular fracture (both of these cases were in the standard treatment group). The AE occurred because one participant (intervention group) developed blistering on their residuum.

Data completeness

There were four standardised measures completed at all three time points: LCI-5, Houghton, PROMIS 3A and PROMIS 8A. The average completion rate across all time points ranged from 89-94%. Data from four clinical assessments (TUG, TUDS, 2mWT and BBS), and an activity monitor worn for a week, were collected at both baseline and final follow-up, and their average completion rate ranged from 92-95%. All the measures were well completed, or attempted, with at least 75% completed in full. Details of the outcome measures and completion rates can be found in Tables 2 and 3.

None of the four standardised measures appeared to show a change between baseline and follow-up. For the clinical assessments, participants with the self-aligning prosthetic ankle-foot walked on average 7.8 metres further at final follow-up compared to baseline. Conversely, participants walking with their non-self-aligning ankle-foot decreased the distance they walked in 2 minutes by an average of 10.8 metres. Scores for the TUG, TUDS and BBS remained largely unchanged across both groups. Activity monitor data revealed that the intervention group increased their average daily standing time more than the standard treatment group at final follow-up compared to baseline (+140.2 vs. +64.5 minutes). Similarly, the intervention group decreased their average daily time spent sitting more than the standard treatment group (-113.6 vs. -34.1 minutes). These findings did not translate to meaningful changes in daily steps. On average, both groups took fewer than 1800 daily steps over 30 minutes throughout the day.

Health economic data

The health economic outcomes are based on the patient-completed questionnaires which incorporated the EQ-5D-5L and a bespoke resource use questionnaire. The average completion rates were 93% and 63%, respectively. Over 89% of participants completed the EQ-5D-5L fully. At baseline, responses to the EQ-5D-5L indicated relatively equal levels of

For peer review only - http://bmjopen.bmj.com/site/about/guidelines.xhtml

health utility between the trial arms. By final follow-up, an increase in mean utility of 0.13 in the intervention group was observed, whilst mean utility in the standard treatment group remained the same. A positive mean difference in favour of the self-aligning prosthetic ankle-foot in the intervention arm remained consistent after adjusting for baseline utility (Table 4).

For the resource use questionnaire, the completion rates did not differ by the respective components, suggesting all sections were acceptable and each type of data was feasible to collect. Resource use was minimal across both primary and secondary/tertiary care for participants in both arms of the trial. The most frequently received care was via outpatient attendances at NHS hospitals (mean (SD): 0.21 (0.51) rising to 2.04 (3.72) visits from baseline to final follow-up for the intervention group; 0.08 (0.27) rising to 1.14 (2.19) visits for the standard treatment group.

Medications were grouped according to comorbidity to investigate their inclusion in a full-scale trial as follows: blood pressure/heart problems, neuropathic pain, diabetes, phantom pain. Participant uptake of the groups of medications explored using the resource use questionnaire was high, and there were some differences between groups, suggesting a micro-costing exercise including medications data may be merited in a full trial. Approximately 73% of participants overall reported taking medications related to blood pressure and/or heart problems, and 62% of participants overall took medications for diabetes. Medication use appeared to increase over the course of the trial for participants in both arms.

The intervention (self-aligning prosthetic ankle-foot) was delivered in the NHS setting by the usual staff providing post-amputation aftercare (prosthetist, physiotherapist). Other than the self-aligning ankle-foot itself, participants received equal care to standard treatment. The cost (at 2020 prices) of the self-aligning ankle-foot used in the trial was £1192 compared to the non-self-aligning version at £287 (although this depends on the exact standard ankle-foot). The additional intervention cost to the NHS of delivering the self-aligning ankle-foot is therefore approximately £905.

Identification of primary outcome measure

The findings from this study have demonstrated feasibility, acceptability and a signal of efficacy for three measures: walking ability with the 2mWT, daily activity with activity monitor data, and quality-of-life with the EQ-5D-5L. We consulted with our patient advisory group (PAG) to inform the appropriateness of a primary outcome to be used in a future full-scale trial. The PAG members reported that, of the three measures, the EQ-5D-5L resonated most strongly with them as it captured the constructs that were important to them (e.g., mobility, pain, usual activities, general health). This measure will be considered for use as the primary outcome in a future trial.

For peer review only - http://bmjopen.bmj.com/site/about/guidelines.xhtml

DISCUSSION

Main findings

The primary objective of this study was to determine the feasibility of conducting a future, full-scale RCT of the effectiveness and cost-effectiveness of a self-aligning prosthetic ankle-foot compared to a standard prosthetic ankle-foot. There are few clinical trials involving older adults living with a lower limb amputation, who are an under-researched but growing group of patients. The retention, consent and completion rates were high, demonstrating that it is feasible to recruit and retain participants to a future trial addressing the research question. While the consent rate may be artificially high, due to screening methods used in the current study, the final retention and completion rates indicate the study was acceptable to participants.

Although we did not meet the recruitment target of 90, this could be related to the strict inclusion criteria used which could be adjusted in a future trial. It is common that the residuum undergoes considerable volume changes within the first year post-amputation, necessitating the fabrication of new prosthetic sockets. As the intervention period of this feasibility study was only 12 weeks long (due to resource constraints), we sought to recruit established prosthesis users who had no current contraindications to wearing their ankle-foot prosthesis, had a stable residuum volume and the same prosthetic socket for ≥ 3 months and were free of open wounds or infections on the residuum. We also only included patients who had a transtibial amputation secondary to a health condition, as this group is most likely categorised as 'limited mobility' and frequently prescribed a non-self-aligning prosthetic ankle-foot. It would be clinically relevant to broaden the eligibility criteria, which would also widen the pool of patients potentially eligible for the trial.

Five AEs, of which four were categorised as serious, occurred throughout this trial. All of the SAEs (one stroke and three falls) were not deemed related to the self-aligning prosthetic ankle-foot. This clinical group is vulnerable to falling, with one recent study stating that over 57% of community-living lower limb prosthesis users fall at least once a year(24) and that 36-75% of fallers experience recurrent falls.(24, 25) The participant who developed blistering on the residuum (the one AE) had been allocated to the intervention group. It is not uncommon for blistering to occur if the skin on the residuum is subjected to increased friction as a result of wearing the prosthesis for longer. In most instances, blisters heal relatively quickly, and the prosthesis user can soon return to normal function with their prosthesis.

Strengths and weaknesses

Prosthetics services are an under-researched area within the NHS. As this was one of few clinical trials conducted in this area in the UK, we now have a better understanding of the challenges of recruiting in this setting, such as the limited research resources at clinical sites

For peer review only - http://bmjopen.bmj.com/site/about/guidelines.xhtml

and variation in how sites organise their patient database systems. One of the strengths of this feasibility study was the broad geographical spread of the recruiting sites in England (North East, North West, East of England, Midlands and South of England); however, the participants recruited across these sites were homogenous in their ethnicity (self-reported as White British, Irish or other). In future, we will develop strategies to maximise participation from patients across different ethnic groups and nations in the United Kingdom.

The majority of participants were recruited after a member of their clinical team screened the patient database for potentially eligible participants and posted them a study invitation pack. Therefore, the majority of the screening forms (79%) were initially completed over the phone before the face-to-face screening process confirmed their final eligibility. A limitation of this feasibility study was our lack of prior knowledge of the management of patient databases which varied across the sites. Four sites used an electronic database for patient records while one used a paper-based system only; one of these sites used three different patient record systems. Not all patient records were up-to-date, resulting in two participants being randomised in error because they did not fulfil the inclusion criteria fully, and they were subsequently withdrawn. In a future trial, it will be essential to gather relevant information about the organisation of patient records at the local participating sites prior to opening that site to facilitate effective recruitment. This would also help sites recruit participants more consistently across the recruitment period.

Results in context with other research

This study was not designed to assess the effectiveness of the self-aligning prosthetic ankle-foot, and no formal comparisons between randomised groups were undertaken as the study was not powered to report effects. Three measures indicated a signal of potential efficacy including the 2-minute walk test (walking ability), daily activity as measured with activity monitors, and quality-of-life as measured with the EQ-5D-5L. Conversely, other measures (including some population-specific measures and common clinical assessments) did not indicate any difference. The findings partly support existing literature that reported improved walking and better quality-of-life for transtibial prosthesis users, classified as ‘limited mobility’, when they used a self-aligning ankle-foot, albeit from cross-sectional study designs and only when performed in a laboratory setting.[9-11] However, a full-scale RCT is needed to provide robust evidence of the effectiveness and cost-effectiveness to inform clinical commissioning policies for the prescription of ankle-foot prostheses in future.

Importantly, we demonstrated that the use of activity monitors, and their implementation using postal delivery, was feasible in this patient group. We believe this was supported by our study procedures, which included regular participant information letters alerting them to the next stages of the trial, and a detailed bespoke leaflet explaining how participants were requested to fit the activity monitor onto the prosthesis themselves. As the monitor

For peer review only - http://bmjopen.bmj.com/site/about/guidelines.xhtml

was affixed to the prosthesis, there were few issues with discomfort or discontinued use. Moreover, the data related to daily steps and stepping time reinforced the largely sedentary behaviour experienced by this clinical group. Our findings are broadly similar to a previous study that reported an average of 1450 (SD 1309) steps/day in a group of older individuals (age: 64 (SD 9) years) with a predominantly transtibial amputation (95% of total participants) as a result of vascular disease when measured over 10 days.[26] Further research is warranted to ascertain whether a more functional prosthetic ankle-foot, compared to current standard care with a non-self-aligning ankle-foot, has the long-term potential to increase daily stepping, which could have important implications for patient benefit.

Health economics

It was feasible to collect the data required for a full-scale trial economic evaluation from this patient population. The high levels of completion suggest acceptability of instruments. The EQ-5D-5L is likely to be sensitive to changes in health states over time. Some changes to the bespoke health resource use questionnaire could be made in future. Use of privately funded healthcare services was negligible so this section could potentially be omitted in a full-scale trial. Participant uptake of the medications in the resource use questionnaire was high and there were some differences between groups suggesting a micro-costing exercise including medications data may be merited in a full-scale trial. Although no resource use of walking aids, adaptations and accessories had an uptake greater than 20%, participants were established prosthesis users who may have already accessed the required adaptations prior to entering the trial. Therefore, if a future trial had broader eligibility criteria to include unestablished prosthesis users, it may be important to retain this item.

The approximate NHS cost of the intervention is ~£900 as it is assumed that the self-aligning prosthetic ankle-foot is delivered in the usual NHS setting and by the usual staff. There are various self-aligning prosthetic ankle-feet available commercially (e.g., using hydraulic mechanisms vs. micro-processor controlled and with actively powered propulsion), at varying costs and suitability (e.g., some prosthetic ankle-feet are heavier and more suitable for different patient needs). At the present time, we have only considered a single prosthetic ankle-foot, but one that is designed for our clinical population of interest. A full health economics evaluation, as part of full-scale trial, would be able to evaluate whether the initial higher cost of the ankle-foot could be offset by reduced use of other health resources (e.g., medications, prosthetic adjustments) and/or increased patient quality-of-life.

For peer review only - http://bmjopen.bmj.com/site/about/guidelines.xhtml

Future research

Based on the findings from this feasibility study, we propose some adaptations to the study protocol for a future full-scale trial. They include:

- broadening the participant eligibility criteria to include new patients with a transtibial amputation who would be categorised as 'limited mobility' by their multi-disciplinary team and therefore likely to receive a non-self-aligning prosthetic ankle-foot;
- lengthening the intervention period of accommodating to the self-aligning ankle-foot, and including a longer follow-up period, would be important for unestablished prosthesis users if the criteria were more inclusive in a full trial;
- refining the screening process to screen more potentially eligible participants, even if some prove ineligible afterwards, with advanced information about site patient databases;
- reviewing the secondary outcome measures; and
- redesigning some aspects of the resource use questionnaire.

CONCLUSION

The STEPFORWARD study demonstrated feasibility to recruit and retain participants and the ability to obtain a variety of complete datasets collected in clinic and via post. We have also evidenced the ability to deliver the trial across multiple prosthetics centres in England. The knowledge gained will be implemented in a future definitive trial where we will determine the effectiveness and cost-effectiveness of a self-aligning prosthetic ankle-foot compared to a non-self-aligning version for people with a transtibial amputation and categorised as 'limited mobility'.

FIGURE CAPTION

Figure 1: CONSORT flowchart for the STEPFORWARD study

For peer review only

For peer review only - http://bmjopen.bmj.com/site/about/guidelines.xhtml

REFERENCES

1. University of Salford. LIMBLESS STATISTICS: Repository for quantitative information on the UK limbless population REFERRED for prosthetics treatment, Annual report 2011-2012. University of Salford; 2013.

2. Davies M, Burdett L, Bowling F, Ahmad N, McClennon J. The epidemiology of major lower-limb amputation in England: a systematic review highlighting methodological differences of reported trials. The Diabetic Foot Journal. 2019;22:53-61.

3. Crawford F, Cezard G, Chappell FM, Murray GD, Price JF, Sheikh A, et al. A systematic review and individual patient data meta-analysis of prognostic factors for foot ulceration in people with diabetes: the international research collaboration for the prediction of diabetic foot ulcerations (PODUS). Health Technology Assessment. 2015;19(57).

4. Sansam K, O'Connor RJ, Neumann V, Bhakta B. Clinicians' perspectives on decision making in lower limb amputee rehabilitation. Journal of Rehabilitation Medicine. 2014;46(5):447-53.

5. Hofstad C, Linde H, Limbeek J, Postema K. Prescription of prosthetic ankle-foot mechanisms after lower limb amputation. The Cochrane database of systematic reviews. 2004(1):CD003978.

6. Agrawal V, Gailey RS, Gaunaurd IA, O'Toole C, Finnieston A, Tolchin R. Comparison of four different categories of prosthetic feet during ramp ambulation in unilateral transtibial amputees. Prosthetics and Orthotics International. 2015;39(5):380-9.

7. Johnson L, De Asha AR, Munjal R, Kulkarni J, Buckley JG. Toe clearance when walking in people with unilateral transtibial amputation: Effects of passive hydraulic ankle. Journal of Rehabilitation Research and Development. 2014;51(3):429-37.

8. Portney LG, Watkins MP. Foundations of clinical research: Applications to practice (Third Edition). New Jersey, USA: Pearson Education; 2009.

9. Barnett CT, Brown OH, Bisele MM, Brown MJ, De Asha AR, Strutzenberger G. Individuals with unilateral transtibial amputation and lower activity levels walk more quickly when using a hydraulically articulating versus rigidly attached prosthetic ankle-foot device. Journal of Prosthetics and Orthotics. 2018;30(3):158-64.

10. Moore R. Effect on stance phase timing asymmetry in individuals with amputation using hydraulic ankle units. Journal of Prosthetics and Orthotics. 2016;28:44-8.

11. Moore R. Patient evaluation of a novel prosthetic foot with hydraulic ankle aimed at persons with amputation with lower activity levels. Journal of Prosthetics and Orthotics. 2017;29:44-7.

12. Goujon H, Bonnet X, Sautreuil P, Maurisset M, Darmon L, Fode P, et al. A functional evaluation of prosthetic foot kinematics during lower-limb amputee gait. Prosthetics and Orthotics International. 2006;30(2):213-23.

13. Mitchell N, Coleman E, Watson J, Bell K, McDaid C, Barnett C, et al. Self-aligning prosthetic device for older patients with vascular-related amputations: protocol for a randomised feasibility study (the STEPFORWARD study). BMJ Open. 2019;9(9):e032924.

For peer review only - http://bmjopen.bmj.com/site/about/guidelines.xhtml

14. Franchignoni F, Orlandini D, Ferriero G, Moscato TA. Reliability, validity, and responsiveness of the locomotor capabilities index in adults with lower-limb amputation undergoing prosthetic training. Archives of Physical Medicine and Rehabilitation. 2004;85(5):743-8.

15. Devlin M, Pauley T, Head K, Garfinkel S. Houghton Scale of prosthetic use in people with lower extremity amputations: Reliability, validity, and responsiveness to change. Archives of Physical Medicine and Rehabilitation. 2004;85(8):1339-44.

16. Morgan SJ, Friedly JL, Amtmann D, Salem R, Hafner BJ. Cross-Sectional Assessment of Factors Related to Pain Intensity and Pain Interference in Lower Limb Prosthesis Users. Archives of Physical Medicine and Rehabilitation. 2017;98(1):105-13.

17. Hurst NP, Kind P, Ruta D, Hunter M, Stubbings A. Measuring health-related quality of life in rheumatoid arthritis: Validity, responsiveness and reliability of EuroQol (EQ-5D). British Journal of Rheumatology. 1997;36(5):551-9.

18. Podsiadlo D, Richardson S. The Timed Up and Go - A test of basic functional mobility for frail elderly persons. Journal of the American Geriatrics Society. 1991;39(2):142-8.

19. Bonnyaud C, Zory R, Pradon D, Vuillerme N, Roche N. Clinical and biomechanical factors which predict Timed Up and Down Stairs test performance in hemiparetic patients. Gait & Posture. 2013;38(3):466-70.

20. Butland RJA, Pang J, Gross ER, Woodcock AA, Geddes DM. 2-minute, 6-minute, and 12-minute walking tests in respiratory-disease. British Medical Journal. 1982;284(6329):1607-8.

21. Major MJ, Fatone S, Roth EJ. Validity and Reliability of the Berg Balance Scale for Community-Dwelling Persons With Lower-Limb Amputation. Archives of Physical Medicine and Rehabilitation. 2013;94(11):2194-202.

22. Health Do. NHS reference costs 2017/18 London2018 [Available from: https://improvement.nhs.uk/resources/reference-costs/#rc1718.

23. Curtis L, Burns A. Unit Costs of Health and Social Care 2016. . Personal Social Services Research Unit: University of Kent; 2016.

24. Kim J, Major MJ, Hafner B, Sawers A. Frequency and Circumstances of Falls Reported by Ambulatory Unilateral Lower Limb Prosthesis Users: A Secondary Analysis. PM&R. 2019;11(4):344-53.

25. Miller WC, Speechley M, Deathe B. The prevalence and risk factors of falling and fear of falling among lower extremity amputees. Archives of Physical Medicine and Rehabilitation. 2001;82(8):1031-7.

26. Miller MJ, Cook PF, Kline PW, Anderson CB, Stevens-Lapsley JE, Christiansen CL. Physical Function and Pre-Amputation Characteristics Explain Daily Step Count after Dysvascular Amputation. PM&R. 2019;11(10):1050-8.

For peer review only - http://bmjopen.bmj.com/site/about/guidelines.xhtml

Table 1: Demographic characteristics according to trial arm

	Self-aligning (n=27)	Standard (n=28)	Overall (n=55)
**Age, years**	N=27	N=28	N=55
Mean (SD)	70.0 (9.2)	67.6 (10.0)	68.8 (9.6)
Median (min, max)	71.5 (52.7, 86.8)	68.3 (51.8, 84.5)	70.7 (51.8, 86.8)
**Gender, n(%)**			
Male	23 (85.2)	24 (85.7)	47 (85.5)
Female	4 (14.8)	4 (14.3)	8 (14.6)
**Ethnicity, n(%)**			
White (British, Irish or Other)	27 (100.0)	28 (100.0)	55 (100.0)
Black (African, Caribbean or Other)	0 (0.0)	0 (0.0)	0 (0.0)
Asian (Indian, Pakistani, Bangladeshi or Other)	0 (0.0)	0 (0.0)	0 (0.0)
Mixed Background	0 (0.0)	0 (0.0)	0 (0.0)
Chinese	0 (0.0)	0 (0.0)	0 (0.0)
**Marital status, n(%)**			
Living alone never married	2 (7.4)	3 (10.7)	5 (9.1)
Living with partner	4 (14.8)	0 (0.0)	4 (7.3)
Married/civil partnership	9 (33.3)	19 (67.9)	28 (50.9)
Separated	2 (7.4)	1 (3.6)	3 (5.5)
Divorced	5 (18.5)	4 (14.3)	9 (16.4)
Widowed	2 (7.4)	1 (3.6)	3 (5.5)
Missing	3 (11.1)	0 (0.0)	3 (5.5)
**Main activity, n(%)**			
Full-time employment	1 (3.7)	3 (10.7)	4 (7.3)
Part-time employment	0 (0.0)	1 (3.6)	1 (1.8)
Self-employed	0 (0.0)	0 (0.0)	0 (0.0)
Unable to work due to poor health	1 (3.7)	5 (17.9)	6 (10.9)
Unemployed	0 (0.0)	1 (3.6)	1 (1.8)
Retired	24 (88.9)	17 (60.7)	41 (74.6)
Other	1 (3.7)	1 (3.6)	2 (3.6)
**Smoking status, n(%)**			
Ex-smoker	15 (55.6)	17 (60.7)	32 (58.2)
Current smoker	1 (3.7)	3 (10.7)	4 (7.3)
Never smoked	11 (40.7)	8 (28.6)	19 (34.6)
**Diabetes status, n(%)**			
Yes	19 (70.4)	18 (64.3)	37 (67.3)
No	8 (29.6)	10 (35.7)	18 (32.7)
**If yes:**			
Type one	5 (26.3)	2 (11.1)	7 (18.9)
Type two	12 (63.2)	13 (72.2)	25 (67.8)
Missing	2 (10.5)	3 (16.7)	5 (13.5)
**Cause of amputation, n(%)[b]**			
Diabetes	14 (51.9)	11 (39.3)	25 (45.5)
Peripheral vascular disease	10 (37.0)	10 (35.7)	20 (36.4)
Blood clot	3 (11.1)	2 (7.1)	5 (9.1)

For peer review only - http://bmjopen.bmj.com/site/about/guidelines.xhtml

Deep vein thrombosis	1 (3.7)	0 (0.0)	1 (1.8)
Aneurysm	0 (0.0)	1 (3.6)	1 (1.8)
Diabetic neuropathy	2 (7.4)	1 (3.6)	3 (5.5)
Other neuropathy	1 (3.7)	0 (0.0)	1 (1.8)
Cancer	1 (3.7)	1 (3.6)	2 (3.6)
Other	5 (18.5)	6 (21.4)	11 (20.0)

For peer review only

For peer review only - http://bmjopen.bmj.com/site/about/guidelines.xhtml

Table 2. Standard outcomes at each time point (baseline, interim and final follow-up), by trial arm and overall

	Self-aligning (n=27)	Standard (n=28)	Overall (n=55)
**LCI-5 overall score (0 – 56): average completion 94%**			
**Baseline**	N=25	N=28	N=53
Mean (SD)	36.4 (12.0)	39.6 (11.2)	39.1 (11.6)
Median (p25, p75)	38 (30.3, 46)	38 (31, 50)	38 (30.3, 47)
**Interim**	N=24	N=25	N=49
Mean (SD)	37.3 (12.3)	38.5 (10.8)	37.9 (11.4)
Median (p25, p75)	39.5 (28.5, 47.5)	41 (30, 44.7)	41 (29, 46)
**Final**	N=25	N=26	N=51
Mean (SD)	37.8 (11.4)	39.2 (11.2)	38.5 (11.2)
Median (p25, p75)	40 (30, 47)	40.5 (29, 49)	40 (29, 47)
**Houghton overall score (0 – 12): average completion: 89%**			
**Baseline**	N=24	N=28	N=52
Mean (SD)	7.8 (2.0)	8.8 (1.8)	8.3 (1.9)
Median (p25, p75)	8 (7.5, 9)	9 (8, 10)	9 (8, 9)
**Interim**	N=20	N=23	N=43
Mean (SD)	8.4 (2.0)	8.5 (1.3)	8.5 (1.6)
Median (p25, p75)	9 (7, 10)	9 (8, 9)	9 (8, 10)
**Final**	N=23	N=26	N=49
Mean (SD)	8.3 (1.9)	8.5 (2.0)	8.4 (1.9)
Median (p25, p75)	8 (8, 10)	9 (8, 9)	9 (8, 9)
**PROMIS 3a overall score (30.7 – 71.8): average completion: 90%**			
**Baseline**	N=27	N=27	N=54
Mean (SD)	41.3 (10.0)	43.1 (9.5)	42.2 (9.7)
Median (p25, p75)	43.5 (30.7, 52.1)	43.5 (30.7, 49.4)	43.5 (30.7, 49.4)
**Interim**	N=21	N=25	N=46
Mean (SD)	43.6 (9.6)	45.6 (10.3)	44.7 (10.0)
Median (p25, p75)	43.5 (30.7, 49.4)	46.3 (40.2, 52.1)	46.3 (30.7, 52.1)
**Final**	N=24	N=22	N=46
Mean (SD)	40.4 (9.4)	42.7 (8.7)	41.4 (9.0)
Median (p25, p75)	40.2 (30.7, 47.9)	44.9 (30.7, 49.4)	43.5 (30.7, 49.4)
**PROMIS 8a overall score (40.7 – 77.0): average completion: 94%**			
**Baseline**	N=27	N=28	N=55
Mean (SD)	52.8 (11.2)	51.7 (9.4)	52.2 (10.2)
Median (p25, p75)	55 (40.7, 63.5)	51.8 (40.7, 58.5)	52.3 (40.7, 60.2)
**Interim**	N=23	N=24	N=47
Mean (SD)	53.5 (11.3)	56.7 (8.9)	55.1 (10.1)
Median (p25, p75)	55 (40.7, 64.1)	58.8 (52.3, 63.2)	57.4 (40.7, 63.5)
**Final**	N=25	N=26	N=51
Mean (SD)	51.2 (11.4)	53.3 (10.0)	52.3 (10.7)
Median (p25, p75)	49.9 (40.7, 60.2)	54.5 (40.7, 60.2)	53.2 (40.7, 60.2)

LCI-5 (Locomotor Capabilities Index-5): higher scores indicate a greater locomotor capability with the prosthesis; Houghton: higher scores indicate greater performance and comfort; PROMIS (Patient-Reported Outcomes Measurement Information System) 3a (pain intensity): coverts raw scores to T-scores, higher scores indicate more intense pain; PROMIS 8a: higher scores indicate more pain interference.

For peer review only

Table 3. Clinical assessments (TUG, TUDS, 2mWT and BBS) at baseline and final follow-up, by trial arm and overall

	Self-aligning (n=27)	Standard (n=28)	Overall (n=55)
**TUG, seconds: average completion 95%**			
**Baseline**	N=27	N=28	N=55
Mean (SD)	20.8 (15.2)	21.3 (23.5)	21.1 (19.7)
Median (p25, p75)	17 (12, 24)	14.5 (11.5, 23)	17 (12, 23)
Unable to complete	0	0	0
**Final**	N=23	N=22	N=45
Mean (SD)	21.0 (19.1)	17.8 (8.4)	19.5 (14.8)
Median (p25, p75)	16 (11, 13)	14.5 (13, 22)	15 (11, 23)
Unable to complete	0	2	0
**TUDS, seconds: average completion 93%**			
**Baseline**	N=17	N=27	N=44
Mean (SD)	61.4 (33.0)	66.9 (38.5)	64.7 (36.2)
Median (p25, p75)	50 (39, 72)	51 (41, 88)	50.5 (41, 88)
Unable to complete	9	1	10
**Final**	N=19	N=21	N=40
Mean (SD)	59.2 (29.2)	68.1 (56.7)	63.9 (45.4)
Median (p25, p75)	51 (33, 79)	48 (39, 74)	48.5 (38, 76.5)
Unable to complete:	4	3	7
**2mWT, metres: average completion 95%**			
**Baseline**	N=27	N=28	N=55
Mean (SD)	81.0 (31.4)	94.7 (33.5)	87.9 (33.5)
Median (p25, p75)	77 (60, 110)	95 (68, 113)	83 (61, 113)
**Final**	N=23	N=24	N=47
Mean (SD)	88.8 (40.0)	83.9 (41.5)	86.3 (39.5)
Median (p25, p75)	80 (62, 120)	80 (57.5, 106)	80 (60, 108)
**BBS, scored 0-56: average completion 92%**			
**Baseline**	N=26	N=27	N=53
Mean (SD)	39.5 (11.3)	42.9 (8.1)	41.3 (9.8)
Median (p25, p75)	41.5 (32, 48)	43 (36, 51)	43 (34, 48)
**Final**	N=22	N=24	N=46
Mean (SD)	40.7 (12.9)	39.4 (11.6)	40.0 (12.1)
Median (p25, p75)	44 (30, 51)	41.5 (33, 49)	41.5 (33, 50)
**Activity monitor: minutes standing per day: average completion 93%**			
**Baseline**	N=26	N=27	N=53
Mean (SD)	869.5 (306.1)	801.6 (228.7)	834.9 (269.0)
Median (p25, p75)	862.6 (638.0, 1036.9)	770.5 (639.1, 908.1)	808.1 (639.1, 969.3)
**Final**	N=22	N=25	N=47
Mean (SD)	1009.7 (304.5)	866.1 (257.9)	933.3 (286.8)
Median (p25, p75)	1021.0 (810.6, 1309.7)	857.0 (750.7, 972.7)	906.7 (757.7, 1162.9)
**Activity monitor: minutes sedentary per day**			
**Baseline**	N=26	N=27	N=53
Mean (SD)	510.3 (295.6)	565.6 (239.9)	538.5 (267.5)
Median (p25, p75)	476.6 (401.1, 783.7)	613.1 (482.9, 742.9)	549.3 (410.3, 745.1)
**Final**	N=22	N=25	N=47

For peer review only - http://bmjopen.bmj.com/site/about/guidelines.xhtml

Mean (SD)	396.7 (297.0)	531.5 (244.2)	468.4 (275.7)
Median (p25, p75)	416.4 (120.0, 553.0)	510.7 (425.1, 626.3)	497.4 (246.7, 619.0)
**Activity monitor: minutes stepping per day**			
**Baseline**	N=26	N=27	N=53
Mean (SD)	29.9 (30.8)	31.1 (24.9)	30.5 (27.7)
Median (p25, p75)	24.7 (9.4, 31.4)	26 (14.5, 40.3)	25.9 (10.6, 34.1)
**Final**	N=22	N=25	N=47
Mean (SD)	27.7 (24.0)	31.2 (25.8)	29.6 (24.8)
Median (p25, p75)	24.1 (3.6, 45.0)	25.9 (17.3, 31.1)	25.7 (10.6, 40.3)
**Activity monitor: steps taken per day**			
**Baseline**	N=26	N=27	N=53
Mean (SD)	1755.5 (1963.7)	1750.9 (1369.6)	1753.2 (1670.9)
Median (p25, p75)	1292.3 (587.1, 1835.7)	1572.9 (802.3, 2234.6)	1448.6 (654.6, 2144.0)
**Final**	N=22	N=25	N=47
Mean (SD)	1673.2 (1594.4)	1836.1 (1647.8)	1759.9 (1607.4)
Median (p25, p75)	1356.4 (204.0, 2719.4)	1510.3 (922.9, 1908.0)	1446.3 (551.4, 2270.0)

TUG (Timed Up and Go test) and TUDS (Timed Up and Down Stair test): faster time indicates better performance; 2mWT (2-minute walk test): further distance indicates better walking ability; BBS (Berg Balance Scale): higher score indicates better balance.

For peer review only

Table 4: EQ-5D-5L mean (SD) utility scores, unadjusted mean difference (95% CI) and adjusted for baseline utility at each time point (baseline, interim and final follow-up), by trial arm

	Self-aligning (n=27)	Standard (n=28)	Unadjusted difference (self-aligning vs. standard) (95% CI)	Adjusted difference (self-aligning vs. standard) (95% CI)
**EQ-5D-5L (average completion 93%)**				
**Baseline**	0.62 (0.30)	0.63 (0.35)	-0.009 (-0.188, 0.169)	-0.009 (-0.188, 0.169)
**Interim**	0.64 (0.18)	0.57 (0.24)	0.067 (-0.055, 0.189)	0.104 (0.001, 0.207)
**Final**	0.75 (0.16)	0.63 (0.32)	0.120 (-0.021, 0.262)	0.117 (0.017, 0.216)

For peer review only

Acknowledgements

This work uses data provided by patients and collected by researchers and members of the NHS who worked collaboratively on the STEPFORWARD trial. The collection of data would not have been possible without the support of the participating NHS Trusts and their staff. The authors would also like to thank the members of the Project Advisory Group (PAG): Dennis Harrison, Peter Wignall, Anthony Gick and Kevin Waller for their invaluable advice on many aspects of the project.

Author contributions

NV led on the conception, design and writing of this manuscript. NM, EC, JW, KB, CMcD, CB, MT, FJ, AS and DH contributed to the design, writing, critical review of intellectual content and final manuscript approval. All authors agree to be accountable for their work. As Chief Investigator, NV takes overall responsibility for the work. EC provided statistical expertise in the study design and development stages of the project and the protocol. NM, JW, and CMcD made substantial contributions to the trial design and management. DH provided particular input to PPI. KB was specifically responsible for the health economic aspects of the study design and CB, MT, FJ and AS were responsible for providing expert clinical support.

Funding statement

This paper presents independent research funded by the National Institute for Health Research (NIHR) under its Research for Patient Benefit (RfPB) Programme (Grant Reference Number PB-PG-0816-20029). Hull University Teaching Hospitals NHS Trust are the sponsors of the study, on behalf of the funder.

Blatchford (Basingstoke, UK) provided the prosthetic feet examined in this research at no cost. The prosthetic feet are currently commercially available devices. Blatchford have had no part in the study design nor are they be involved directly in the research, its subsequent analysis and ultimate dissemination.

Disclaimer

The views expressed are those of the author(s) and not necessarily those of the NHS, the NIHR, the Department of Health and Social Care, or Blatchford.

Patient and public involvement

Patients and public involvement members were involved in the design, conduct, reporting and dissemination plans of this research.

Patient consent for publication

Not required

Competing interests

None declared.

Ethics approval

Ethical approval by Yorkshire & The Humber – Leeds West Research Ethics Committee (IRAS project ID 232950; REC reference 18/YH/0089) and HRA approval were obtained on 4th May 2018.

For peer review only - http://bmjopen.bmj.com/site/about/guidelines.xhtml

Data availability statement

The data that support the findings of this study are available from the corresponding author, [NV], upon reasonable request.

For peer review only

For peer review only - http://bmjopen.bmj.com/site/about/guidelines.xhtml

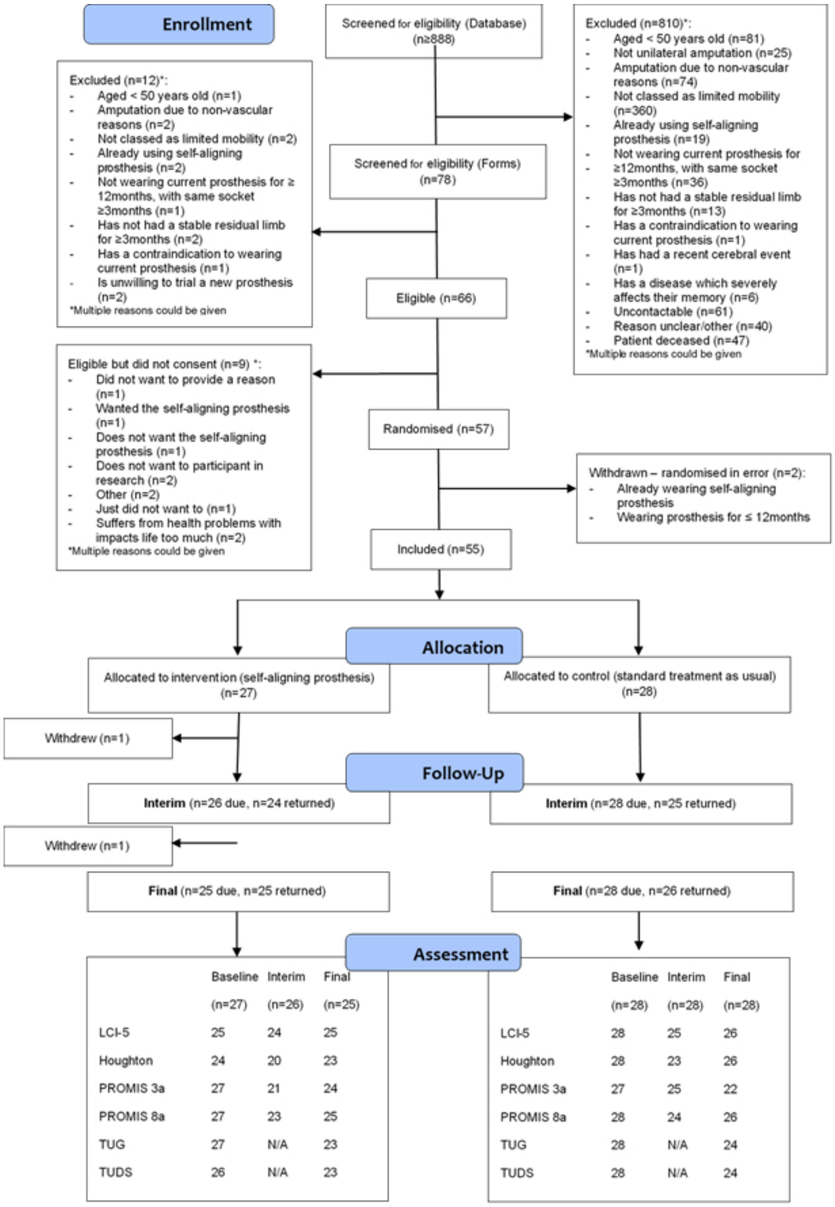

Figure 1: CONSORT flowchart for the STEPFORWARD study

161x233mm (96 x 96 DPI)

Reporting checklist for randomised trial.

Based on the CONSORT guidelines.

Instructions to authors

Complete this checklist by entering the page numbers from your manuscript where readers will find each of the items listed below.

Your article may not currently address all the items on the checklist. Please modify your text to include the missing information. If you are certain that an item does not apply, please write "n/a" and provide a short explanation.

Upload your completed checklist as an extra file when you submit to a journal.

In your methods section, say that you used the CONSORTreporting guidelines, and cite them as:

Schulz KF, Altman DG, Moher D, for the CONSORT Group. CONSORT 2010 Statement: updated guidelines for reporting parallel group randomised trials

		Reporting Item	Page Number
**Title and Abstract**			
Title	#1a	Identification as a randomized trial in the title.	1
Abstract	#1b	Structured summary of trial design, methods, results, and conclusions	2
**Introduction**			

For peer review only

Background and objectives	#2a	Scientific background and explanation of rationale	4
Background and objectives	#2b	Specific objectives or hypothesis	n/a

Methods

Trial design	#3a	Description of trial design (such as parallel, factorial) including allocation ratio.	5
Trial design	#3b	Important changes to methods after trial commencement (such as eligibility criteria), with reasons	n/a
Participants	#4a	Eligibility criteria for participants	5
Participants	#4b	Settings and locations where the data were collected	5 and 7
Interventions	#5	The experimental and control interventions for each group with sufficient details to allow replication, including how and when they were actually administered	6
Outcomes	#6a	Completely defined prespecified primary and secondary outcome measures, including how and when they were assessed	6
Sample size	#7a	How sample size was determined.	n/a
Sample size	#7b	When applicable, explanation of any interim analyses and stopping guidelines	n/a

For peer review only

Randomization - Sequence generation	#8a	Method used to generate the random allocation sequence.	
5			
Randomization - Sequence generation	#8b	Type of randomization; details of any restriction (such as blocking and block size)	
5			
Randomization - Allocation concealment mechanism	#9	Mechanism used to implement the random allocation sequence (such as sequentially numbered containers), describing any steps taken to conceal the sequence until interventions were assigned	5
Randomization - Implementation	#10	Who generated the allocation sequence, who enrolled participants, and who assigned participants to interventions	5
Blinding	#11a	If done, who was blinded after assignment to interventions (for example, participants, care providers, those assessing outcomes) and how.	5
Blinding	#11b	If relevant, description of the similarity of interventions	n/a
Statistical methods	#12a	Statistical methods used to compare groups for primary and secondary outcomes	6
Statistical methods	#12b	Methods for additional analyses, such as subgroup analyses and adjusted analyses	7

For peer review only

Outcomes	#6b	Any changes to trial outcomes after the trial commenced, with reasons	n/a
**Results**			
Participant flow diagram (strongly recommended)	#13a	For each group, the numbers of participants who were randomly assigned, received intended treatment, and were analysed for the primary outcome	7
Participant flow	#13b	For each group, losses and exclusions after randomization, together with reason	8
Recruitment	#14a	Dates defining the periods of recruitment and follow-up	7
Recruitment	#14b	Why the trial ended or was stopped	n/a
Baseline data	#15	A table showing baseline demographic and clinical characteristics for each group	7
Numbers analysed	#16	For each group, number of participants (denominator) included in each analysis and whether the analysis was by original assigned groups	7
Outcomes and estimation	#17a	For each primary and secondary outcome, results for each group, and the estimated effect size and its precision (such as 95% confidence interval)	7
Outcomes and estimation	#17b	For binary outcomes, presentation of both absolute and relative effect sizes is recommended	n/a

For peer review only

Ancillary analyses	#18	Results of any other analyses performed, including subgroup analyses and adjusted analyses, distinguishing pre-specified from exploratory	n/a
Harms	#19	All important harms or unintended effects in each group (For specific guidance see CONSORT for harms)	8
**Discussion**			
Limitations	#20	Trial limitations, addressing sources of potential bias, imprecision, and, if relevant, multiplicity of analyses	11
Interpretation	#22	Interpretation consistent with results, balancing benefits and harms, and considering other relevant evidence	10
Registration	#23	Registration number and name of trial registry	5
**Other Information**			
Protocol	#24	Where the full trial protocol can be accessed, if available	5
Funding	#25	Sources of funding and other support (such as supply of drugs), role of funders	14

The CONSORT checklist is distributed under the terms of the Creative Commons Attribution License CC-BY. This checklist was completed on 23. September 2020 using https://www.goodreports.org/, a tool made by the EQUATOR Network in collaboration with Penelope.ai

For peer review only

BMJ Open

The STEPFORWARD study: a randomised controlled feasibility trial of a self-aligning prosthetic ankle-foot for older patients with vascular-related amputations

Journal:	*BMJ Open*
Manuscript ID	bmjopen-2020-045195.R1
Article Type:	Original research
Date Submitted by the Author:	01-Dec-2020
Complete List of Authors:	Vanicek, Natalie; University of Hull, Sport, Health and Exercise Science<br>Coleman, Elizabeth; University of York, Health Sciences<br>Watson, Judith; University of York, Health Sciences<br>Bell, Kerry; University of York, Health Sciences<br>McDaid, Catriona; University of York, Health Sciences<br>Barnett, Cleveland; Nottingham Trent University, School of Science and Tecnology<br>Twiste, Martin; University of Salford<br>Jepson, Fergus; Lancashire Teaching Hospitals NHS Foundation Trust, Specialist Mobility Rehabilitation Centre<br>Salawu, Abayomi; Hull University Teaching Hospitals NHS Trust, Disability Medicine and Rehabilitation Unit<br>Harrison, Dennis<br>Mitchell, Natasha; University of York, York Trials Unit, Department of Health Sciences
**Primary Subject Heading**:	Rehabilitation medicine
Secondary Subject Heading:	Patient-centred medicine
Keywords:	REHABILITATION MEDICINE, Clinical trials < THERAPEUTICS, Foot & ankle < ORTHOPAEDIC & TRAUMA SURGERY, Musculoskeletal disorders < ORTHOPAEDIC & TRAUMA SURGERY

SCHOLARONE™
Manuscripts

I, the Submitting Author has the right to grant and does grant on behalf of all authors of the Work (as defined in the below author licence), an exclusive licence and/or a non-exclusive licence for contributions from authors who are: i) UK Crown employees; ii) where BMJ has agreed a CC-BY licence shall apply, and/or iii) in accordance with the terms applicable for US Federal Government officers or employees acting as part of their official duties; on a worldwide, perpetual, irrevocable, royalty-free basis to BMJ Publishing Group Ltd ("BMJ") its licensees and where the relevant Journal is co-owned by BMJ to the co-owners of the Journal, to publish the Work in this journal and any other BMJ products and to exploit all rights, as set out in our licence.

The Submitting Author accepts and understands that any supply made under these terms is made by BMJ to the Submitting Author unless you are acting as an employee on behalf of your employer or a postgraduate student of an affiliated institution which is paying any applicable article publishing charge ("APC") for Open Access articles. Where the Submitting Author wishes to make the Work available on an Open Access basis (and intends to pay the relevant APC), the terms of reuse of such Open Access shall be governed by a Creative Commons licence – details of these licences and which Creative Commons licence will apply to this Work are set out in our licence referred to above.

Other than as permitted in any relevant BMJ Author's Self Archiving Policies, I confirm this Work has not been accepted for publication elsewhere, is not being considered for publication elsewhere and does not duplicate material already published. I confirm all authors consent to publication of this Work and authorise the granting of this licence.

For peer review only

Title page:

The STEPFORWARD study: a randomised controlled feasibility trial of a self-aligning prosthetic ankle-foot for older patients with vascular-related amputations

Authors: Natalie Vanicek[1], Elizabeth Coleman[2], Judith Watson[2], Kerry Bell[2], Catriona McDaid[2], Cleveland Barnett[3], Martin Twiste[4], Fergus Jepson[5], Abayomi Salawu[6], Dennis Harrison[7] and Natasha Mitchell[2]

Corresponding author: Professor Natalie Vanicek
Department of Sport, Health and Exercise Science
Faculty of Health Sciences
University of Hull
Hull
HU6 7RX
E: n.vanicek@hull.ac.uk
T: 01482 463607

Author affiliations

[1] Department of Sport, Health and Exercise Science, University of Hull, Hull, UK
n.vanicek@hull.ac.uk Orcid iD 000-0002-9602-3172

[2] York Trials Unit, Department of Health Sciences, University of York, York, UK
izzy.coleman@york.ac.uk Orcid iD 0000-0003-4210-1865
jude.watson@york.ac.uk Orcid iD 0000-0003-0694-3854
kerry.bell@york.ac.uk Orcid iD 0000-0001-5124-138X
catriona.mcdaid@york.ac.uk Orcid iD 0000-0002-3751-7260
natasha.mitchell@york.ac.uk Orcid iD 0000-0002-6904-2976

[3] School of Science and Technology, Nottingham Trent University, Nottingham, UK
cleveland.barnett@ntu.ac.uk Orcid iD 0000-0001-6898-9095

[4] School of Health and Society, University of Salford, Manchester, UK
m.twiste@salford.ac.uk Orcid iD 0000-0003-2724-3283

[5] Specialist Mobility Rehabilitation Centre, Lancashire Teaching Hospitals NHS Foundation Trust, Preston, UK
Fergus.Jepson@lthtr.nhs.uk

[6] Disability Medicine and Rehabilitation Unit, Hull University Teaching Hospitals NHS Trust, Hull, UK
abayomi.salawu@hey.nhs.uk Orcid iD 0000-0003-2496-0826

[7] Public Involvement Member
dharrisonpublic@gmail.com

Keywords

Randomised controlled trial, feasibility, self-aligning, ankle-foot prosthesis, transtibial amputation, acceptability, vascular

Word count: 4,726 words

For peer review only - http://bmjopen.bmj.com/site/about/guidelines.xhtml

ABSTRACT

Objectives: To determine the feasibility of conducting a full-scale randomised controlled trial (RCT) of the effectiveness and cost-effectiveness of a self-aligning prosthetic ankle-foot compared with a standard prosthetic ankle-foot.

Design: Multi-centre parallel group feasibility RCT.

Setting: Five prosthetics centres in England recruiting from July 2018 to August 2019.

Participants: Adults aged ≥ 50 years with a vascular-related or non-traumatic transtibial amputation for one year or longer, categorised as having 'limited community mobility' and using a non-self-aligning ankle-foot.

Intervention: Participants were randomised into one of two groups for 12 weeks: self-aligning prosthetic ankle-foot or existing non-self-aligning prosthetic ankle-foot.

Outcomes: Feasibility measures: recruitment, consent and retention rates; and completeness of questionnaire and clinical assessment datasets across multiple time points. Feasibility of collecting daily activity data with wearable technology and health resource use data with a bespoke questionnaire.

Results: Fifty-five participants were randomised (61% of the target 90 participants): n=27 self-aligning ankle-foot group, n=28 non-self-aligning ankle-foot group. Fifty-one participants were included in the final analysis (71% of the target number of participants). The consent rate and retention at final follow-up were 86% and 93%, respectively. The average recruitment rate was 1.25 participants/site/month (95% CI 0.39 to 2.1). Completeness of questionnaires ranged from 89-94%, and clinical assessment were 92-95%, including the activity monitor data. The average completion rates for the EQ-5D-5L and bespoke resource use questionnaire were 93% and 63%, respectively.

Conclusions: This feasibility trial recruited and retained participants who were categorised as having 'limited community mobility' following a transtibial amputation. The high retention rate of 93% indicated the trial was acceptable to participants and feasible to deliver as a full-scale RCT. The findings support a future, fully-powered evaluation of the effectiveness and cost-effectiveness of a self-aligning prosthetic ankle-foot compared to a standard non-self-aligning version with some adjustments to the trial design and delivery.

Trial registration

ISRCTN15043643

For peer review only

ARTICLE SUMMARY

Strengths and limitations of this study

- This was one of few clinical trials in prosthetics services conducted in the UK. The study design is a multi-centre, randomised controlled trial in an under-researched clinical population involving prosthetics.
- The completeness of subjective and objective assessment measures, administered in a clinical setting and via post, was high.
- Feasibility and acceptability were ascertained with quantitative and trial procedure data.
- Recruitment was lower and slower than anticipated, but the ability to retain participants was high.
- Effectiveness and cost-effectiveness of a self-aligning ankle-foot will need to be established in a definitive full-scale trial.

For peer review only

For peer review only - http://bmjopen.bmj.com/site/about/guidelines.xhtml

INTRODUCTION

In the UK, there are approximately 6,000 new referrals to prosthetics services every year. Most of these referrals are patients aged over 50 years who have had an amputation at the transtibial (below-knee) level.[1] Major lower limb amputations result from long-term diabetes mellitus, coronary and peripheral vascular diseases.[2, 3] Consequently, most new referrals for a prosthesis involve older adults who have other health comorbidities and who are categorised as having 'limited community mobility'. These patients are an under-researched group, with few studies focused on their mobility with a prosthesis.

Many factors determine the type of prosthesis that a patient receives, such as estimation of patient outcomes and goals, and the healthcare budget.[4] A Cochrane Review reported there was insufficient evidence from robust studies to support the "overall superiority of any individual type of prosthetic ankle-foot mechanism".[5] There are also no standardised criteria for the prescription of ankle-foot prostheses. Practice varies across UK centres and is frequently cost-driven. Subsequently, the majority of older people with a transtibial amputation receive a standard, non-self-aligning ankle-foot, such as the non-articulated solid ankle cushioned heel (SACH), uniaxial or multiaxial prosthetic foot. These prosthetic ankle-feet have limited functionality. The SACH foot has a solid 'ankle' that does not allow any movement. The uniaxial ankle-foot is only capable of moving about a single axis with some ankle dorsi- and plantar-flexion; the multiaxial ankle-foot can rotate about multiple axes, allowing ankle dorsi- and plantar-flexion and eversion-inversion. The recoil mechanism to return to a plantigrade position makes the uni- and multi-axial ankle-feet unable to self-align to inclined surfaces. This requires the user to make compensatory actions at proximal joints (compensations at the hip bilaterally and intact knee of the affected limb) and at the intact ankle to achieve stability with a 'foot flat' position when walking on slopes.[6, 7] These actions result in greater asymmetry between the two limbs and increase the metabolic requirements of walking.[6] This can make walking more difficult and tiring. Our public involvement members reported that a poorly functioning prosthesis can contribute to sedentary behaviour, pain and more frequent visits to prosthetics centres and other healthcare services, which may lead to disuse and poorer quality-of-life. Therefore, identifying a more functionally suitable prosthesis could benefit patients in meaningful ways following amputation.

A 'family' of prosthetic ankle-feet that use a hydraulic mechanism to self-align to sloped surfaces have been designed to attenuate many of the burdens associated with standard, non-self-aligning ankle-feet. These prosthetic ankle-feet have been designed for users of varying ability (more active users, K3-K4 classification) and also for users categorised as having 'limited community mobility' (K2 users).[8] The hydraulic mechanism allows the prosthetic ankle-foot to self-align to sloped surfaces (due to ground reaction forces) and remain in a dorsiflexed position throughout the swing phase. This can improve ground clearance and securing the biological knee, which is important for falls prevention.[9] Previous

For peer review only - http://bmjopen.bmj.com/site/about/guidelines.xhtml

laboratory-based studies reported that a self-aligning ankle-foot reduced residuum-socket interface pressures in active users,[10] which could alleviate pain in the residuum longer-term, improved walking[11] and enhanced quality of life[12, 13] in users with lower activity levels. More functional prostheses may improve patient function and mobility following a lower limb amputation.[14] However, the effects of a self-aligning prosthesis have not been evaluated with robust randomised controlled trials (RCTs) in a community setting. In particular there is a lack of research involving participants who have had a non-traumatic (e.g., vascular-related) amputation. A self-aligning ankle-foot is more expensive than a non-self-aligning version by approximately £900 for the K2-user version (at 2020 UK prices) with variations in price across manufacturers. However, the potential patient benefits, such as improved mobility, better quality-of-life, and reduced falls, could offset future healthcare and socioeconomic costs.

There is a need for a RCT of the effectiveness and cost-effectiveness of a self-aligning prosthetic ankle-foot for older patients, with a vascular-related or non-traumatic transtibial amputation, compared to a standard, non-self-aligning ankle-foot. The STEPFORWARD study used a mixed-methods approach to determine feasibility of conducting such a study. A feasibility study was considered important in advance of a full-scale RCT given the lack of large community-based RCTs in this field. A feasibility study addresses the question of whether a specific study can be done, whether it should proceed and, if so, how.[15] It is not designed to address the question of whether an intervention works. This article reports the feasibility as determined by the quantitative data, which was assessed according to: (1) participant recruitment, including time to recruit, consent and retention rates and adverse events; (2) completeness of data across multiple time points, including health economics data while piloting a bespoke health resource use questionnaire; and (3) day-to-day activity measured with wearable technology. The findings from this work could be relevant to clinical practitioners, researchers and policy makers.

METHODS

Study design and participants

The STEPFORWARD trial was a multi-centre, parallel group, randomised controlled feasibility trial to assess the possibility of conducting a full-scale RCT in the future, which would be adequately powered to answer the primary research question. The trial received favourable ethical approval from the National Health Service Yorkshire and the Humber—Leeds West Research Ethics Committee (ref:18/YH/0089) and Health Research Authority in May 2018. The trial was registered on the ISRCTN clinical trial registry (ISRCTN15043643). The full study protocol, with detailed inclusion/exclusion criteria and recruitment/screening pathways, has been published[16] and is briefly described below.

We aimed to recruit 90 participants who were aged ≥ 50 years with a unilateral transtibial amputation for ≥1 year due to vascular, neurological or life-limiting reasons, who were

For peer review only - http://bmjopen.bmj.com/site/about/guidelines.xhtml

categorised as having 'limited community mobility', with a stable residuum, and using a non-self-aligning prosthetic ankle-foot (e.g., SACH, uniaxial, multiaxial (e.g., Multiflex) or other K1/K2 feet). We excluded anyone who had a contraindication to wearing their current prosthesis or the self-aligning ankle-foot (according to the manufacturer's specifications), had experienced a recent cerebrovascular event or had a disease affecting their memory. Allowing for 20% attrition, we anticipated 72 participants in our final analysis.

Recruitment and randomisation

We used a two-stage screening process to identify eligible patients; full details are provided in the published protocol.[16] In summary, potential participants were identified by database screening or during routine appointments to five prosthetics centres in England from July 2018 to August 2019. They were posted a study invitation pack and asked to return a Consent to Contact form if they were interested in being contacted about the trial. A member of their multidisciplinary team then contacted them and completed the first section of the Screening Form over the telephone. If potentially eligible, they were invited into clinic to complete the outstanding screening questions, which had to be done face-to-face, and provide their written consent. Following consent, eligible patients completed all baseline measures before being randomised into one of two trial arms: keep their non-self-aligning prosthetic ankle-foot (standard treatment group) or receive a new self-aligning ankle-foot (intervention group). Participants were randomised individually, stratified according to prosthetics centre on a 1:1 basis, by a telephone randomisation service set up by York Trials Unit. Blinding of the participants and investigators was not possible because of the nature of the intervention.

Standard treatment and intervention

Participants in the standard treatment group continued using their normal prosthetic ankle-foot and had access to all clinical services as normal. They were asked to carry on with their normal daily routine during the intervention period.

Participants in the intervention group were fitted with the self-aligning prosthetic ankle-foot by their regular prosthetist. The self-aligning ankle-foot used in this trial was the Avalon^{K2}, manufactured by Blatchford, Basingstoke, UK (Patent reg: 5336386), which was already commercially available and could be prescribed under the national health service (NHS). Once fitted, participants were asked to ambulate with it as they would normally with any new prosthetic ankle-foot for approximately 12 weeks after fitting (intervention period). At the end of the study, participants in the intervention group only could keep the self-aligning ankle-foot if they preferred it. Participants in standard treatment group were not offered the self-aligning ankle-foot at the end of the study as it is not known whether it is better or more acceptable to users than the standard prosthesis.

For peer review only - http://bmjopen.bmj.com/site/about/guidelines.xhtml

Outcome measures

The primary outcome related to feasibility data on recruitment, consent, and retention rates. Reasons for non-participation or withdrawal were recorded where possible. We assessed the completeness of data across multiple time points: baseline, interim and final follow-ups. The baseline and final assessments were conducted at the participant's usual prosthetics centre. At the interim follow-up, only questionnaire data were collected via post. Data were gathered from questionnaires (baseline demographics and bespoke health resource use questionnaires, the Locomotor Capabilities Index (LCI-5),[17] Houghton Scale,[18] the Patient-Reported Outcomes Measurement Information System (PROMIS) Short-Form v1.0 Pain (3a and 8a) questionnaires[19] and EQ-5D-5L[20]) and from clinical assessments (Timed Up and Go (TUG) test,[21] Timed Up and Down Stairs (TUDS) test,[22] 2-minute walk test (2mWT)[23] and Berg Balance Scale (BBS)[24]). Data related to daily step count and time spent walking were collected from a loaned activity monitor (activPAL4™, PAL Technologies Ltd, Glasgow, UK) worn on the prosthesis for one week. At baseline, all participants wore their normal (non-self-aligning) prosthetic ankle-foot during the clinical assessments and activity monitor data collection; at final follow-up they wore whichever prosthetic ankle-foot they were using currently. Adverse events (AEs) related to the prosthesis only, and any serious adverse events (SAEs), were recorded throughout the study.

Statistical analyses

The recruitment rate was reported monthly, and overall, by site. An average monthly recruitment rate was calculated, with a 95% confidence interval (CI) estimated from the data collected. The number of eligible patients and those approached for consent was summarised overall, by site, using counts and percentages.

Baseline data were summarised by trial arm, as randomised, with no formal comparison between the groups. Continuous data were reported descriptively and categorical data by counts and percentages. Completion rates of all the clinical outcome measures were reported by trial arm and overall.

Health economic analysis

The health economics work investigated the feasibility of collecting self-reported patient use of health service resources (e.g., primary care visits, prescription medication) over the study period and to evaluate the appropriateness of these data. This information would help to refine the bespoke questionnaire in a future trial. A full cost-effectiveness analysis was not completed as this was a feasibility study. Nonetheless, the costing approach was undertaken from an NHS perspective; unit costs were derived from established national costing sources such as NHS Reference Costs[25] and Personal Social Services Research Unit costs of health and social care.[26]

For peer review only - http://bmjopen.bmj.com/site/about/guidelines.xhtml

RESULTS

Feasibility assessment

Recruitment

Over 880 potential participants were initially screened by searching patient databases across the five prosthetics centres. Seventy-eight were identified as potentially eligible after the first screening, but 12 were subsequently excluded following the final, second screening. Eighty-six percent of those screened, who were found to be fully eligible, consented to participating (55 of 64 participants) in the trial. The denominator was 64 as two were randomised in error and subsequently withdrawn (Figure 1). As we used a two-stage screening process, only potential participants who met the initial inclusion criteria were approached; those uninterested in being involved during the initial screening were not screened further. Therefore, the consent rate may be artificially high. Reasons for non-participation are also presented in Figure 1. In total, 55 participants (61% of the target 90 participants) were included and randomised as follows: 27 to the self-aligning prosthetic ankle-foot (intervention) group and 28 to the non-self-aligning (standard treatment) group.

Initial recruitment was slower than anticipated, therefore two additional recruiting sites were included midway through the trial, bringing the total number of participating sites to five. The average recruitment rate was 1.25 participants/site/month (95% CI 0.39 to 2.1) ranging from 0.83 to 2.2 participants per month. Each site recruited approximately the same number of participants (mean=11, range=9-13 participants), despite them being open to recruitment for different lengths of time (range=5.0-13.7 months).

Participants

The mean (SD) age of participants in this study was 68.8 (9.6) years, ranging from 51.8 to 86.8 years. Most participants were male (85.5%) and all classified themselves as White British, Irish or other. The most common reason for amputation was diabetes (45.5%). Full participant characteristics are described in Table 1.

Follow-up and adverse events

In addition to the two people randomised in error and withdrawn, a further two participants who were allocated to the intervention group withdrew from the study: one as a result of having suffered a stroke and one reported the self-aligning prosthetic ankle-foot was too heavy. At final follow-up, 51 of the 55 participants returned the questionnaires giving a retention rate of 93%. This was 71% of the planned target of 72 participants included in the final analysis.

A total of four SAEs and one AE were reported. The SAEs were due to the following reasons: stroke; fall and femoral fracture when not wearing their prosthesis (both of these cases occurred in the intervention group); fall and hip fracture, leading to hospitalisation and subsequently death; and recurrent falls with fibular fracture (both of these cases were in the

For peer review only - http://bmjopen.bmj.com/site/about/guidelines.xhtml

standard treatment group). The AE occurred because one participant (intervention group) developed blistering on their residuum.

Data completeness

There were four standardised measures completed at all three time points: LCI-5, Houghton, PROMIS 3A and PROMIS 8A. The average completion rate across all time points ranged from 89-94%. Data from four clinical assessments (TUG, TUDS, 2mWT and BBS), and an activity monitor worn for a week, were collected at both baseline and final follow-up, and their average completion rate ranged from 92-95%. All the measures were well completed, or attempted, with at least 75% completed in full. Details of the outcome measures and completion rates can be found in Tables 2 and 3.

None of the four standardised measures appeared to show a change between baseline and follow-up. For the 2-minute walk test, participants with the self-aligning prosthetic ankle-foot walked on average 7.8 metres further at final follow-up (88.8 (40.0) metres) compared to baseline (81.0 (31.4) metres). Conversely, participants walking with their non-self-aligning ankle-foot decreased the distance they walked in 2 minutes by an average of 10.8 metres (94.7 (33.5) metres at baseline vs. 83.9 (41.5) metres at final follow-up). Scores for the TUG, TUDS and BBS remained largely unchanged across both groups. Activity monitor data revealed that the daily time spent stepping between baseline and follow-up was mostly unchanged for both groups, which was approximately 30 minutes per day. Conversely, the number of steps taken per day decreased by 82.3 metres in the intervention group (1755.5 (1963.7) metres at baseline vs. 1673.2 (1594.4) metres at final follow-up) yet increased by 85.2 metres in the standard treatment group (1750.9 (1369.9) metres at baseline vs. 1836.1 (1647.8) metres at final follow-up). In both groups, the variability in daily stepping was high.

Health economic data

The health economic outcomes are based on the patient-completed questionnaires, which incorporated the EQ-5D-5L and a bespoke resource use questionnaire. The average completion rates were 93% and 63%, respectively. Over 89% of participants completed the EQ-5D-5L fully. At baseline, responses to the EQ-5D-5L indicated relatively equal levels of health utility between the trial arms. By final follow-up, an increase in mean utility of 0.13 in the intervention group was observed, whilst mean utility in the standard treatment group remained the same. A positive mean difference in favour of the self-aligning prosthetic ankle-foot in the intervention group remained consistent after adjusting for baseline utility (Table 4).

For the resource use questionnaire, the completion rates did not differ by the respective components, suggesting all sections were acceptable and each type of data was feasible to collect. Resource use was minimal across both primary and secondary/tertiary care for participants in both arms of the trial. The most frequently received care was via outpatient

For peer review only - http://bmjopen.bmj.com/site/about/guidelines.xhtml

attendances at NHS hospitals (mean (SD): 0.21 (0.51) rising to 2.04 (3.72) visits from baseline to final follow-up for the intervention group; 0.08 (0.27) rising to 1.14 (2.19) visits for the standard treatment group.

Medications were grouped according to comorbidity to investigate their inclusion in a full-scale trial as follows: blood pressure/heart problems, neuropathic pain, diabetes, phantom pain. Participant uptake of the groups of medications explored using the resource use questionnaire was high, and there were some differences between groups, suggesting a micro-costing exercise including medications data may be merited in a full-scale trial. Approximately 73% of participants overall reported taking medications related to blood pressure and/or heart problems, and 62% of participants overall took medications for diabetes. Medication use appeared to increase over the course of the trial for participants in both arms.

The intervention (self-aligning prosthetic ankle-foot) was delivered in the NHS setting by the usual staff providing post-amputation aftercare (prosthetist, physiotherapist). Other than the self-aligning ankle-foot itself, participants received equal care to standard treatment. The cost (at 2020 UK prices) of the self-aligning ankle-foot used in the trial was £1192 compared to the non-self-aligning version at £287 (although this depends on the exact standard ankle-foot). The additional intervention cost to the NHS of delivering the self-aligning ankle-foot is therefore approximately £905.

Identification of primary outcome measure

The findings from this study have demonstrated feasibility, acceptability and a signal of efficacy for three measures: walking ability with the 2mWT, daily activity with activity monitor data, and quality-of-life with the EQ-5D-5L. We consulted with our patient advisory group (PAG) to inform the appropriateness of a primary outcome to be used in a future full-scale trial. The PAG members reported that, of the three measures, the EQ-5D-5L resonated most strongly with them as it captured the constructs that were important to them (e.g., mobility, pain, usual activities, general health). This measure will be considered for use as the primary outcome in a future trial.

DISCUSSION

Main findings

The primary objective of this study was to determine the feasibility of conducting a future, full-scale RCT of the effectiveness and cost-effectiveness of a self-aligning prosthetic ankle-foot compared to a standard prosthetic ankle-foot. There are few clinical trials involving older adults living with a lower limb amputation, who are an under-researched but growing group of patients. The consent, retention and completion rates were high, demonstrating that it is feasible to recruit and retain participants to a future trial addressing the research question. While the consent rate may be artificially high, due to screening methods used in

For peer review only - http://bmjopen.bmj.com/site/about/guidelines.xhtml

the current study, the final retention and completion rates indicate the study was acceptable to participants.

We did not meet the recruitment target of 90. Given that only 7.4% of screened patients met the eligibility criteria (though only 7.2% were truly eligible, due to the randomisation in error), it is important to consider whether the criteria were too restrictive and should be adjusted in a future trial. It is common that the residuum undergoes considerable volume changes within the first year post-amputation, necessitating the fabrication of new prosthetic sockets. As the intervention period of this feasibility study was only 12 weeks long (due to resource constraints), we sought to recruit established prosthesis users who had no current contraindications to wearing their ankle-foot prosthesis, had a stable residuum volume and the same prosthetic socket for ≥ 3 months and were free of open wounds or infections on the residuum. We also only included patients who had a transtibial amputation secondary to a health condition, as this group is most likely categorised as having 'limited community mobility' and frequently prescribed a non-self-aligning prosthetic ankle-foot. It would be clinically relevant to broaden the eligibility criteria to include any patient with a transtibial amputation within this mobility category, regardless of cause and time since amputation, in a future study. This would widen the pool of patients potentially eligible for the trial. However, we do not have data from the feasibility study to assess impact on recruitment and it would be important for the future trial to have an internal pilot with clear stop-go criteria to confirm the feasibility of a modified study design.

Five AEs, of which four were categorised as serious, occurred throughout this trial. All of the SAEs (one stroke and three falls) were not deemed related to the self-aligning prosthetic ankle-foot. This clinical group is vulnerable to falling, with one recent study stating that over 57% of community-living lower limb prosthesis users fall at least once a year[27] and that 36-75% of fallers experience recurrent falls.[27, 28] The participant who developed blistering on the residuum (the one AE) had been allocated to the intervention group. It is not uncommon for blistering to occur if the skin on the residuum is subjected to increased friction as a result of wearing the prosthesis for longer. In most instances, blisters heal relatively quickly, and the prosthesis user can soon return to normal function with their prosthesis.

Results in context with other research

We are not aware of any other randomised feasibility trials investigating prosthetic ankle-feet.

Outcome measures

This study was not designed to assess the effectiveness of the self-aligning prosthetic ankle-foot, and no formal comparisons between randomised groups were undertaken as the study was not powered to report effects. Three measures indicated a signal of potential efficacy including the 2-minute walk test (walking ability), daily activity as measured with activity

For peer review only - http://bmjopen.bmj.com/site/about/guidelines.xhtml

monitors, and quality-of-life as measured with the EQ-5D-5L. Conversely, other measures (including some population-specific measures and common clinical assessments) did not indicate any difference.

We demonstrated that the use of activity monitors, and their implementation using postal delivery, was feasible in this patient group. We believe this was supported by our study procedures, which included regular participant information letters alerting them to the next stages of the trial, and a detailed bespoke leaflet explaining how participants were requested to fit the activity monitor onto the prosthesis themselves. As the monitor was affixed to the prosthesis, there were few issues with discomfort or discontinued use. Trials using subjective outcomes, where the outcome measure is self-reported by a participant or assessment of an event requires exercise of judgement by an observer, may overestimate the treatment effect.[29] Therefore, it was important to establish the feasibility of using this more objective outcome in a future trial.

On average, both groups took fewer than 1800 daily steps over 30 minutes throughout the day. The data related to daily steps and stepping time reinforced the largely sedentary behaviour experienced by this clinical group. Our findings are broadly similar to a previous study that reported an average of 1450 (SD 1309) steps/day in a group of older individuals (age: 64 (SD 9) years) with a predominantly transtibial amputation (95% of total participants) as a result of vascular disease when measured over 10 days.[30] Further research is warranted to ascertain whether a more functional prosthetic ankle-foot, compared to current standard care with a non-self-aligning ankle-foot, has the long-term potential to increase daily stepping, which could have important implications for patient benefit.

Health economics

It was feasible to collect the data required for a full-scale trial economic evaluation from this patient population. The high levels of completion suggest acceptability of instruments. The EQ-5D-5L is likely to be sensitive to changes in health states over time. Some changes to the bespoke health resource use questionnaire could be made in future. Use of privately funded healthcare services was negligible so this section could potentially be omitted in a full-scale trial. Participant uptake of the medications in the resource use questionnaire was high and there were some differences between groups suggesting a micro-costing exercise including medications data may be merited in a full-scale trial. Although no resource use of walking aids, adaptations and accessories had an uptake greater than 20%, participants were established prosthesis users who may have already accessed the required adaptations prior to entering the trial. Therefore, if a future trial had broader eligibility criteria to include unestablished prosthesis users, it may be important to retain this item.

The approximate NHS cost of the intervention is ~£900 above current standard care as it is assumed that the self-aligning prosthetic ankle-foot is delivered in the usual NHS setting and

For peer review only - http://bmjopen.bmj.com/site/about/guidelines.xhtml

by the usual staff. There are various self-aligning prosthetic ankle-feet available commercially (e.g., using hydraulic mechanisms vs. micro-processor controlled and with actively powered propulsion), at varying costs and suitability (e.g., some prosthetic ankle-feet are heavier and more suitable for different patient needs). At the present time, we have only considered a single prosthetic ankle-foot, but one that is designed for our clinical population of interest. A full health economics evaluation, as part of full-scale trial, would be able to evaluate whether the initial higher cost of the ankle-foot could be offset by reduced use of other health resources (e.g., medications, prosthetic adjustments) and/or increased patient quality-of-life.

Strengths and weaknesses

Prosthetics services are an under-researched area within the NHS. As this was one of few clinical trials conducted in this area in the UK, we now have a better understanding of the challenges of recruiting in this setting, such as the limited research resources at clinical sites and variation in how sites organise their patient database systems. The recruiting sites in England provided a wide geographical spread (North East, North West, East of England, Midlands and South of England); however, the participants recruited across these sites were homogenous in their ethnicity (self-reported as White British, Irish or other). In future, we will develop strategies to maximise participation from patients across different ethnic groups and nations in the United Kingdom.

The majority of participants were recruited after a member of their clinical team screened the patient database for potentially eligible participants and posted them a study invitation pack. Therefore, the majority of the screening forms (79%) were initially completed over the phone before the face-to-face screening process confirmed their final eligibility. A limitation of this feasibility study was our lack of prior knowledge of the management of patient databases, which varied across the sites. Four sites used an electronic database for patient records while one used a paper-based system only; one of these sites used three different patient record systems. Not all patient records were up-to-date, resulting in two participants being randomised in error because they did not fulfil the inclusion criteria fully, and they were subsequently withdrawn. In a future trial, it will be essential to gather relevant information about the organisation of patient records at the local participating sites prior to opening that site to facilitate effective recruitment. This would also help sites recruit participants more consistently across the recruitment period. We were concerned at the outset about the risk of 'resentful demoralisation' amongst participants allocated to usual care and were mindful of this in developing the patient information for the study. The fact that adherence to the study processes and completion of outcome measures were similar across groups provides some reassurance in this regard. However, this would need continuing attention in the design of a full-scale trial.

For peer review only - http://bmjopen.bmj.com/site/about/guidelines.xhtml

Future research

Based on the findings from this feasibility study, we propose some adaptations to the study protocol for a future full-scale trial. They include:

- broadening the participant eligibility criteria to include new patients with a transtibial amputation, who would be categorised as having 'limited community mobility' by their multi-disciplinary team, and therefore likely to receive a non-self-aligning prosthetic ankle-foot;
- lengthening the intervention period of accommodating to the self-aligning ankle-foot, and including a longer follow-up period, would be important for unestablished prosthesis users if the criteria were more inclusive in a full-scale trial;
- refining the screening process to screen more potentially eligible participants, even if some prove ineligible afterwards, with advanced information about site patient databases;
- reviewing the secondary outcome measures;
- redesigning some aspects of the health resource use questionnaire; and
- approaching other prosthetics centres nationally to explore their willingness to be involved in a full-scale trial.

CONCLUSION

Although we did not reach our target sample, due to slower than anticipated recruitment, 71% of expected participants were in the final analysis, owing to our retention rate of 93%. Therefore, we believe the STEPFORWARD study demonstrated feasibility to recruit and retain participants and the ability to obtain a variety of complete datasets collected in clinic and via post. We have also evidenced the ability to deliver the trial across multiple prosthetics centres in England. The knowledge gained and lessons learned can be implemented in a future definitive trial to determine the effectiveness and cost-effectiveness of a self-aligning prosthetic ankle-foot compared to a non-self-aligning version for people with a transtibial amputation and categorised as having 'limited community mobility'.

FIGURE CAPTION

Figure 1: CONSORT flowchart for the STEPFORWARD study

For peer review only - http://bmjopen.bmj.com/site/about/guidelines.xhtml

REFERENCES

1. University of Salford. LIMBLESS STATISTICS: Repository for quantitative information on the UK limbless population REFERRED for prosthetics treatment, Annual report 2011-2012. University of Salford; 2013.

2. Davies M, Burdett L, Bowling F, Ahmad N, McClennon J. The epidemiology of major lower-limb amputation in England: a systematic review highlighting methodological differences of reported trials. Diabet Foot J. 2019;22:53-61.

3. Crawford F, Cezard G, Chappell FM, Murray GD, Price JF, Sheikh A, et al. A systematic review and individual patient data meta-analysis of prognostic factors for foot ulceration in people with diabetes: the international research collaboration for the prediction of diabetic foot ulcerations (PODUS). Health Technol Assess. 2015;19(57).

4. Sansam K, O'Connor RJ, Neumann V, Bhakta B. Clinicians' perspectives on decision making in lower limb amputee rehabilitation. J Rehabil Med. 2014;46(5):447-53.

5. Hofstad C, Linde H, Limbeek J, Postema K. Prescription of prosthetic ankle-foot mechanisms after lower limb amputation. Cochrane Database Syst Rev. 2004(1):CD003978.

6. Askew GN, McFarlane LA, Minetti AE, Buckley JG. Energy cost of ambulation in trans-tibial amputees using a dynamic-response foot with hydraulic versus rigid 'ankle': insights from body centre of mass dynamics. J Neuroeng Rehabil. 2019;16(39).

7. Vrieling AH, van Keeken HG, Schoppen T, Otten E, Halbertsma JPK, Hof AL, et al. Uphill and downhill walking in unilateral lower limb amputees. Gait Posture. 2008; 28(2):235-242.

8. Agrawal V, Gailey RS, Gaunaurd IA, O'Toole C, Finnieston A, Tolchin R. Comparison of four different categories of prosthetic feet during ramp ambulation in unilateral transtibial amputees. Prosthet Orthot Int. 2015;39(5):380-9.

9. Johnson L, De Asha AR, Munjal R, Kulkarni J, Buckley JG. Toe clearance when walking in people with unilateral transtibial amputation: Effects of passive hydraulic ankle. J Rehabil Res Dev. 2014;51(3):429-37.

10. De Asha AR, Johnson L, Munjal R, Kulkarni J, Buckley JG. Attenuation of centre-of-pressure trajectory fluctuations under the prosthetic foot when using an articulating hydraulic ankle attachment compared to fixed attachment. Clin Biomech. 2013;28(2):218-24.

11. Barnett CT, Brown OH, Bisele MM, Brown MJ, De Asha AR, Strutzenberger G. Individuals with unilateral transtibial amputation and lower activity levels walk more quickly when using a hydraulically articulating versus rigidly attached prosthetic ankle-foot device. J Prosthet Orthot. 2018;30(3):158-64.

12. Moore R. Effect on stance phase timing asymmetry in individuals with amputation using hydraulic ankle units. J Prosthet Orthot. 2016;28:44-8.

13. Moore R. Patient evaluation of a novel prosthetic foot with hydraulic ankle aimed at persons with amputation with lower activity levels. J Prosthet Orthot. 2017;29:44-7.

For peer review only - http://bmjopen.bmj.com/site/about/guidelines.xhtml

14. Goujon H, Bonnet X, Sautreuil P, Maurisset M, Darmon L, Fode P, et al. A functional evaluation of prosthetic foot kinematics during lower-limb amputee gait. Prosthet Orthot Int. 2006;30(2):213-23.

15. Eldridge SM, Lancaster GA, Campbell MJ, Thabane L, Hopewell S, Coleman CL, et al. Defining feasibility and pilot studies in preparation for randomised controlled trials: Development of a conceptual framework. PLoS ONE. 2016;11(3):e0150205.

16. Mitchell N, Coleman E, Watson J, Bell K, McDaid C, Barnett C, et al. Self-aligning prosthetic device for older patients with vascular-related amputations: protocol for a randomised feasibility study (the STEPFORWARD study). BMJ Open. 2019;9(9):e032924.

17. Franchignoni F, Orlandini D, Ferriero G, Moscato TA. Reliability, validity, and responsiveness of the locomotor capabilities index in adults with lower-limb amputation undergoing prosthetic training. Arch Phys Med Rehabil. 2004;85(5):743-8.

18. Devlin M, Pauley T, Head K, Garfinkel S. Houghton Scale of prosthetic use in people with lower extremity amputations: Reliability, validity, and responsiveness to change. Arch Phys Med Rehabil. 2004;85(8):1339-44.

19. Morgan SJ, Friedly JL, Amtmann D, Salem R, Hafner BJ. Cross-sectional assessment of factors related to pain ontensity and pain interference in lower limb prosthesis users. Arch Phys Med Rehabil. 2017;98(1):105-13.

20. Hurst NP, Kind P, Ruta D, Hunter M, Stubbings A. Measuring health-related quality of life in rheumatoid arthritis: Validity, responsiveness and reliability of EuroQol (EQ-5D). Br J Rheumatol. 1997;36(5):551-9.

21. Podsiadlo D, Richardson S. The timed "Up & Go": A test of basic functional mobility for frail elderly persons. J Am Geriatr Soc. 1991;39(2):142-8.

22. Bonnyaud C, Zory R, Pradon D, Vuillerme N, Roche N. Clinical and biomechanical factors which predict timed up and down stairs test performance in hemiparetic patients. Gait Posture. 2013;38(3):466-70.

23. Butland RJA, Pang J, Gross ER, Woodcock AA, Geddes DM. Two- ,six-, and 12-minute walking tests in respiratory-disease. Br Med J. 1982;284(6329):1607-8.

24. Major MJ, Fatone S, Roth EJ. Validity and reliability of the Berg Balance Scale for community-dwelling persons with lower-limb amputation. Arch Phys Med Rehabil. 2013;94(11):2194-202.

25. Department of Health. NHS reference costs 2017/18. London. 2018 [Available from: https://improvement.nhs.uk/resources/reference-costs/#rc1718.

26. Curtis L, Burns A. Unit Costs of Health and Social Care 2016. Personal Social Services Research Unit: University of Kent; 2016.

27. Kim J, Major MJ, Hafner B, Sawers A. Frequency and circumstances of falls reported by ambulatory unilateral lower limb prosthesis users: A secondary analysis. PM R. 2019;11(4):344-53.

For peer review only - http://bmjopen.bmj.com/site/about/guidelines.xhtml

28. Miller WC, Speechley M, Deathe B. The prevalence and risk factors of falling and fear of falling among lower extremity amputees. Arch Phys Med Rehabil. 2001;82(8):1031-7.

29. Wood L, Egger M, Gluud LL, Schulz KE, Juni P, Altman DG, Gluud C, Martin RM, Wood AJG, Sterne JAC. Empirical evidence of bias in treatment effect estimates in controlled trials with different interventions and outcomes: meta-epidemiological study. Br Med J. 2008;336(7644):601-5.

30. Miller MJ, Cook PF, Kline PW, Anderson CB, Stevens-Lapsley JE, Christiansen CL. Physical function and pre-amputation characteristics explain daily step count after dysvascular amputation. PM R. 2019;11(10):1050-8.

For peer review only

Table 1: Demographic characteristics according to trial arm

	Self-aligning (n=27)	Standard (n=28)	Overall (n=55)
**Age, years**	N=27	N=28	N=55
Mean (SD)	70.0 (9.2)	67.6 (10.0)	68.8 (9.6)
Median (min, max)	71.5 (52.7, 86.8)	68.3 (51.8, 84.5)	70.7 (51.8, 86.8)
**Gender, n(%)**			
Male	23 (85.2)	24 (85.7)	47 (85.5)
Female	4 (14.8)	4 (14.3)	8 (14.6)
**Ethnicity, n(%)**			
White (British, Irish or Other)	27 (100.0)	28 (100.0)	55 (100.0)
Black (African, Caribbean or Other)	0 (0.0)	0 (0.0)	0 (0.0)
Asian (Indian, Pakistani, Bangladeshi or Other)	0 (0.0)	0 (0.0)	0 (0.0)
Mixed Background	0 (0.0)	0 (0.0)	0 (0.0)
Chinese	0 (0.0)	0 (0.0)	0 (0.0)
**Marital status, n(%)**			
Living alone never married	2 (7.4)	3 (10.7)	5 (9.1)
Living with partner	4 (14.8)	0 (0.0)	4 (7.3)
Married/civil partnership	9 (33.3)	19 (67.9)	28 (50.9)
Separated	2 (7.4)	1 (3.6)	3 (5.5)
Divorced	5 (18.5)	4 (14.3)	9 (16.4)
Widowed	2 (7.4)	1 (3.6)	3 (5.5)
Missing	3 (11.1)	0 (0.0)	3 (5.5)
**Main activity, n(%)**			
Full-time employment	1 (3.7)	3 (10.7)	4 (7.3)
Part-time employment	0 (0.0)	1 (3.6)	1 (1.8)
Self-employed	0 (0.0)	0 (0.0)	0 (0.0)
Unable to work due to poor health	1 (3.7)	5 (17.9)	6 (10.9)
Unemployed	0 (0.0)	1 (3.6)	1 (1.8)
Retired	24 (88.9)	17 (60.7)	41 (74.6)
Other	1 (3.7)	1 (3.6)	2 (3.6)
**Smoking status, n(%)**			
Ex-smoker	15 (55.6)	17 (60.7)	32 (58.2)
Current smoker	1 (3.7)	3 (10.7)	4 (7.3)
Never smoked	11 (40.7)	8 (28.6)	19 (34.6)
**Diabetes status, n(%)**			
Yes	19 (70.4)	18 (64.3)	37 (67.3)
No	8 (29.6)	10 (35.7)	18 (32.7)
**If yes:**			
Type one	5 (26.3)	2 (11.1)	7 (18.9)
Type two	12 (63.2)	13 (72.2)	25 (67.8)
Missing	2 (10.5)	3 (16.7)	5 (13.5)
**Cause of amputation, n(%)[a]**			
Diabetes	14 (51.9)	11 (39.3)	25 (45.5)
Peripheral vascular disease	10 (37.0)	10 (35.7)	20 (36.4)
Blood clot	3 (11.1)	2 (7.1)	5 (9.1)

For peer review only - http://bmjopen.bmj.com/site/about/guidelines.xhtml

Deep vein thrombosis	1 (3.7)	0 (0.0)	1 (1.8)
Aneurysm	0 (0.0)	1 (3.6)	1 (1.8)
Diabetic neuropathy	2 (7.4)	1 (3.6)	3 (5.5)
Other neuropathy	1 (3.7)	0 (0.0)	1 (1.8)
Cancer	1 (3.7)	1 (3.6)	2 (3.6)
Other	5 (18.5)	6 (21.4)	11 (20.0)

[a] Multiple options could be selected

For peer review only

Table 2. Standard outcomes at each time point (baseline, interim and final follow-up), by trial arm and overall

	Self-aligning (n=27)	Standard (n=28)	Overall (n=55)
**LCI-5 overall score (0 – 56): average completion 94%**			
**Baseline**	N=25	N=28	N=53
Mean (SD)	36.4 (12.0)	39.6 (11.2)	39.1 (11.6)
Median (p25, p75)	38 (30.3, 46)	38 (31, 50)	38 (30.3, 47)
**Interim**	N=24	N=25	N=49
Mean (SD)	37.3 (12.3)	38.5 (10.8)	37.9 (11.4)
Median (p25, p75)	39.5 (28.5, 47.5)	41 (30, 44.7)	41 (29, 46)
**Final**	N=25	N=26	N=51
Mean (SD)	37.8 (11.4)	39.2 (11.2)	38.5 (11.2)
Median (p25, p75)	40 (30, 47)	40.5 (29, 49)	40 (29, 47)
**Houghton overall score (0 – 12): average completion: 89%**			
**Baseline**	N=24	N=28	N=52
Mean (SD)	7.8 (2.0)	8.8 (1.8)	8.3 (1.9)
Median (p25, p75)	8 (7.5, 9)	9 (8, 10)	9 (8, 9)
**Interim**	N=20	N=23	N=43
Mean (SD)	8.4 (2.0)	8.5 (1.3)	8.5 (1.6)
Median (p25, p75)	9 (7, 10)	9 (8, 9)	9 (8, 10)
**Final**	N=23	N=26	N=49
Mean (SD)	8.3 (1.9)	8.5 (2.0)	8.4 (1.9)
Median (p25, p75)	8 (8, 10)	9 (8, 9)	9 (8, 9)
**PROMIS 3a overall score (30.7 – 71.8): average completion: 90%**			
**Baseline**	N=27	N=27	N=54
Mean (SD)	41.3 (10.0)	43.1 (9.5)	42.2 (9.7)
Median (p25, p75)	43.5 (30.7, 52.1)	43.5 (30.7, 49.4)	43.5 (30.7, 49.4)
**Interim**	N=21	N=25	N=46
Mean (SD)	43.6 (9.6)	45.6 (10.3)	44.7 (10.0)
Median (p25, p75)	43.5 (30.7, 49.4)	46.3 (40.2, 52.1)	46.3 (30.7, 52.1)
**Final**	N=24	N=22	N=46
Mean (SD)	40.4 (9.4)	42.7 (8.7)	41.4 (9.0)
Median (p25, p75)	40.2 (30.7, 47.9)	44.9 (30.7, 49.4)	43.5 (30.7, 49.4)
**PROMIS 8a overall score (40.7 – 77.0): average completion: 94%**			
**Baseline**	N=27	N=28	N=55
Mean (SD)	52.8 (11.2)	51.7 (9.4)	52.2 (10.2)
Median (p25, p75)	55 (40.7, 63.5)	51.8 (40.7, 58.5)	52.3 (40.7, 60.2)
**Interim**	N=23	N=24	N=47
Mean (SD)	53.5 (11.3)	56.7 (8.9)	55.1 (10.1)
Median (p25, p75)	55 (40.7, 64.1)	58.8 (52.3, 63.2)	57.4 (40.7, 63.5)
**Final**	N=25	N=26	N=51
Mean (SD)	51.2 (11.4)	53.3 (10.0)	52.3 (10.7)
Median (p25, p75)	49.9 (40.7, 60.2)	54.5 (40.7, 60.2)	53.2 (40.7, 60.2)

LCI-5 (Locomotor Capabilities Index-5): higher scores indicate a greater locomotor capability with the prosthesis; Houghton: higher scores indicate greater performance and comfort; PROMIS (Patient-Reported Outcomes Measurement Information System) 3a (pain intensity): coverts raw scores to T-scores, higher scores indicate more intense pain; PROMIS 8a: higher scores indicate more pain interference.

For peer review only - http://bmjopen.bmj.com/site/about/guidelines.xhtml

Table 3. Clinical assessments (TUG, TUDS, 2mWT and BBS) at baseline and final follow-up, by trial arm and overall

	Self-aligning (n=27)	Standard (n=28)	Overall (n=55)
**TUG, seconds: average completion 95%**			
**Baseline**	N=27	N=28	N=55
Mean (SD)	20.8 (15.2)	21.3 (23.5)	21.1 (19.7)
Median (p25, p75)	17 (12, 24)	14.5 (11.5, 23)	17 (12, 23)
Unable to complete	0	0	0
**Final**	N=23	N=22	N=45
Mean (SD)	21.0 (19.1)	17.8 (8.4)	19.5 (14.8)
Median (p25, p75)	16 (11, 13)	14.5 (13, 22)	15 (11, 23)
Unable to complete	0	2	0
**TUDS, seconds: average completion 93%**			
**Baseline**	N=17	N=27	N=44
Mean (SD)	61.4 (33.0)	66.9 (38.5)	64.7 (36.2)
Median (p25, p75)	50 (39, 72)	51 (41, 88)	50.5 (41, 88)
Unable to complete	9	1	10
**Final**	N=19	N=21	N=40
Mean (SD)	59.2 (29.2)	68.1 (56.7)	63.9 (45.4)
Median (p25, p75)	51 (33, 79)	48 (39, 74)	48.5 (38, 76.5)
Unable to complete:	4	3	7
**2mWT, metres: average completion 95%**			
**Baseline**	N=27	N=28	N=55
Mean (SD)	81.0 (31.4)	94.7 (33.5)	87.9 (33.5)
Median (p25, p75)	77 (60, 110)	95 (68, 113)	83 (61, 113)
**Final**	N=23	N=24	N=47
Mean (SD)	88.8 (40.0)	83.9 (41.5)	86.3 (39.5)
Median (p25, p75)	80 (62, 120)	80 (57.5, 106)	80 (60, 108)
**BBS, scored 0-56: average completion 92%**			
**Baseline**	N=26	N=27	N=53
Mean (SD)	39.5 (11.3)	42.9 (8.1)	41.3 (9.8)
Median (p25, p75)	41.5 (32, 48)	43 (36, 51)	43 (34, 48)
**Final**	N=22	N=24	N=46
Mean (SD)	40.7 (12.9)	39.4 (11.6)	40.0 (12.1)
Median (p25, p75)	44 (30, 51)	41.5 (33, 49)	41.5 (33, 50)
**Activity monitor: minutes stepping per day**			
**Baseline**	N=26	N=27	N=53
Mean (SD)	29.9 (30.8)	31.1 (24.9)	30.5 (27.7)
Median (p25, p75)	24.7 (9.4, 31.4)	26 (14.5, 40.3)	25.9 (10.6, 34.1)
**Final**	N=22	N=25	N=47
Mean (SD)	27.7 (24.0)	31.2 (25.8)	29.6 (24.8)
Median (p25, p75)	24.1 (3.6, 45.0)	25.9 (17.3, 31.1)	25.7 (10.6, 40.3)
**Activity monitor: steps taken per day**			
**Baseline**	N=26	N=27	N=53
Mean (SD)	1755.5 (1963.7)	1750.9 (1369.6)	1753.2 (1670.9)
Median (p25, p75)	1292.3 (587.1, 1835.7)	1572.9 (802.3, 2234.6)	1448.6 (654.6, 2144.0)
**Final**	N=22	N=25	N=47

For peer review only - http://bmjopen.bmj.com/site/about/guidelines.xhtml

Mean (SD)	1673.2 (1594.4)	1836.1 (1647.8)	1759.9 (1607.4)
Median (p25, p75)	1356.4 (204.0, 2719.4)	1510.3 (922.9, 1908.0)	1446.3 (551.4, 2270.0)

TUG (Timed Up and Go test) and TUDS (Timed Up and Down Stair test): faster time indicates better performance; 2mWT (2-minute walk test): further distance indicates better walking ability; BBS (Berg Balance Scale): higher score indicates better balance.

For peer review only

Table 4: EQ-5D-5L mean (SD) utility scores, unadjusted mean difference (95% CI) and adjusted for baseline utility at each time point (baseline, interim and final follow-up), by trial arm

	**Self-aligning (n=27)**	**Standard (n=28)**	**Unadjusted difference (self-aligning vs. standard) (95% CI)**	**Adjusted difference (self-aligning vs. standard) (95% CI)**
**EQ-5D-5L (average completion 93%)**				
**Baseline**	0.62 (0.30)	0.63 (0.35)	-0.009 (-0.188, 0.169)	-0.009 (-0.188, 0.169)
**Interim**	0.64 (0.18)	0.57 (0.24)	0.067 (-0.055, 0.189)	0.104 (0.001, 0.207)
**Final**	0.75 (0.16)	0.63 (0.32)	0.120 (-0.021, 0.262)	0.117 (0.017, 0.216)

For peer review only

Twitter
Follow Natalie Vanicek @NatalieVanicek and Cleveland Barnett @Cleve_Barnett

Acknowledgements
This work uses data provided by patients and collected by researchers and members of the NHS who worked collaboratively on the STEPFORWARD trial. The collection of data would not have been possible without the support of the participating NHS Trusts and their staff. The authors would also like to thank the members of the Project Advisory Group (PAG): Dennis Harrison, Peter Wignall, Anthony Gick and Kevin Waller for their invaluable advice on many aspects of the project.

Author contributions
NV led on the conception, design and writing of this manuscript. NM, EC, JW, KB, CMcD, CB, MT, FJ, AS and DH contributed to the design, writing, critical review of intellectual content and final manuscript approval. All authors agree to be accountable for their work. As Chief Investigator, NV takes overall responsibility for the work. EC provided statistical expertise in the study design and development stages of the project and the protocol. NM, JW, and CMcD made substantial contributions to the trial design and management. DH provided particular input to PPI. KB was specifically responsible for the health economic aspects of the study design and CB, MT, FJ and AS were responsible for providing expert clinical support.

Funding statement
This paper presents independent research funded by the National Institute for Health Research (NIHR) under its Research for Patient Benefit (RfPB) Programme (Grant Reference Number PB-PG-0816-20029). Hull University Teaching Hospitals NHS Trust are the sponsors of the study, on behalf of the funder.

Blatchford (Basingstoke, UK) provided the prosthetic feet examined in this research at no cost. The prosthetic feet are currently commercially available devices. Blatchford have had no part in the study design nor are they be involved directly in the research, its subsequent analysis and ultimate dissemination.

Disclaimer
The views expressed are those of the author(s) and not necessarily those of the NHS, the NIHR, the Department of Health and Social Care, or Blatchford.

Patient and public involvement
Patients and public involvement members were involved in the design, conduct, reporting and dissemination plans of this research.

Patient consent for publication
Not required

Competing interests
None declared.

For peer review only

Ethics approval

Ethical approval by Yorkshire & The Humber – Leeds West Research Ethics Committee (IRAS project ID 232950; REC reference 18/YH/0089) and HRA approval were obtained on 4th May 2018.

Data availability statement

The data that support the findings of this study are available from the corresponding author, [NV], upon reasonable request.

For peer review only

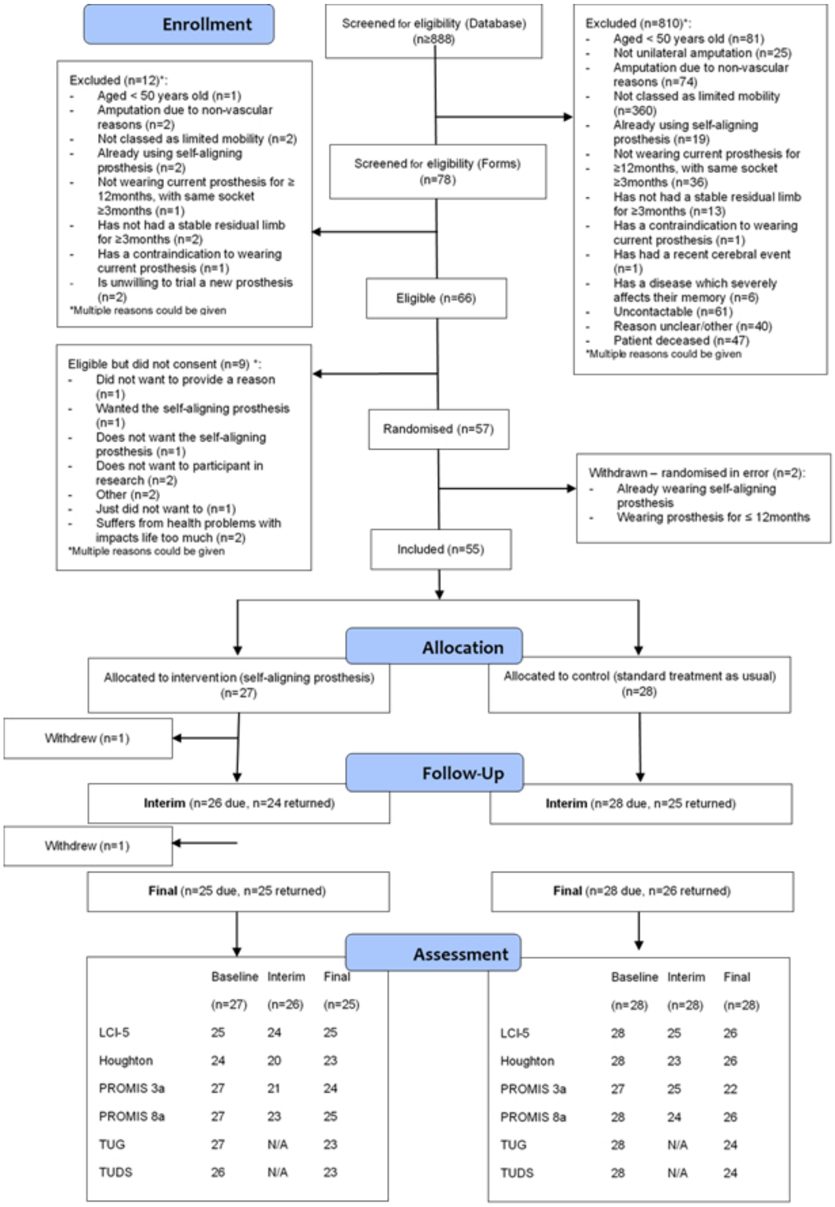

Figure 1: CONSORT flowchart for the STEPFORWARD study

161x233mm (96 x 96 DPI)

CONSORT 2010 checklist of information to include when reporting a pilot or feasibility trial*

Section/Topic	Item No	Checklist item	Reported on page No
**Title and abstract**			
	1a	Identification as a pilot or feasibility randomised trial in the title	1
	1b	Structured summary of pilot trial design, methods, results, and conclusions (for specific guidance see CONSORT abstract extension for pilot trials)	2
**Introduction**			
Background and objectives	2a	Scientific background and explanation of rationale for future definitive trial, and reasons for randomised pilot trial	4-5
	2b	Specific objectives or research questions for pilot trial	5
**Methods**			
Trial design	3a	Description of pilot trial design (such as parallel, factorial) including allocation ratio	5
	3b	Important changes to methods after pilot trial commencement (such as eligibility criteria), with reasons	n/a
Participants	4a	Eligibility criteria for participants	5-6
	4b	Settings and locations where the data were collected	6
	4c	How participants were identified and consented	6
Interventions	5	The interventions for each group with sufficient details to allow replication, including how and when they were actually administered	6
Outcomes	6a	Completely defined prespecified assessments or measurements to address each pilot trial objective specified in 2b, including how and when they were assessed	7
	6b	Any changes to pilot trial assessments or measurements after the pilot trial commenced, with reasons	n/a
	6c	If applicable, prespecified criteria used to judge whether, or how, to proceed with future definitive trial	n/a
Sample size	7a	Rationale for numbers in the pilot trial	6
	7b	When applicable, explanation of any interim analyses and stopping guidelines	n/a
Randomisation:			
Sequence generation	8a	Method used to generate the random allocation sequence	n/a
	8b	Type of randomisation(s); details of any restriction (such as blocking and block size)	6
Allocation concealment mechanism	9	Mechanism used to implement the random allocation sequence (such as sequentially numbered containers), describing any steps taken to conceal the sequence until interventions were assigned	n/a

For peer review only

Implementation	10	Who generated the random allocation sequence, who enrolled participants, and who assigned participants to interventions	6
Blinding	11a	If done, who was blinded after assignment to interventions (for example, participants, care providers, those assessing outcomes) and how	n/a
	11b	If relevant, description of the similarity of interventions	n/a
Statistical methods	12	Methods used to address each pilot trial objective whether qualitative or quantitative	7
**Results**			
Participant flow (a diagram is strongly recommended)	13a	For each group, the numbers of participants who were approached and/or assessed for eligibility, randomly assigned, received intended treatment, and were assessed for each objective	8, Figure 1
	13b	For each group, losses and exclusions after randomisation, together with reasons	8, Figure 1
Recruitment	14a	Dates defining the periods of recruitment and follow-up	6
	14b	Why the pilot trial ended or was stopped	n/a
Baseline data	15	A table showing baseline demographic and clinical characteristics for each group	Table 1
Numbers analysed	16	For each objective, number of participants (denominator) included in each analysis. If relevant, these numbers should be by randomised group	Tables 1-4
Outcomes and estimation	17	For each objective, results including expressions of uncertainty (such as 95% confidence interval) for any estimates. If relevant, these results should be by randomised group	Tables 1-4
Ancillary analyses	18	Results of any other analyses performed that could be used to inform the future definitive trial	n/a
Harms	19	All important harms or unintended effects in each group (for specific guidance see CONSORT for harms)	8
	19a	If relevant, other important unintended consequences	n/a
**Discussion**			
Limitations	20	Pilot trial limitations, addressing sources of potential bias and remaining uncertainty about feasibility	13
Generalisability	21	Generalisability (applicability) of pilot trial methods and findings to future definitive trial and other studies	10-13
Interpretation	22	Interpretation consistent with pilot trial objectives and findings, balancing potential benefits and harms, and considering other relevant evidence	10-13
	22a	Implications for progression from pilot to future definitive trial, including any proposed amendments	14
**Other information**			
Registration	23	Registration number for pilot trial and name of trial registry	2
Protocol	24	Where the pilot trial protocol can be accessed, if available	5-6
Funding	25	Sources of funding and other support (such as supply of drugs), role of funders	24
	26	Ethical approval or approval by research review committee, confirmed with reference number	25

For peer review only

Citation: Eldridge SM, Chan CL, Campbell MJ, Bond CM, Hopewell S, Thabane L, et al. CONSORT 2010 statement: extension to randomised pilot and feasibility trials. BMJ. 2016;355.

*We strongly recommend reading this statement in conjunction with the CONSORT 2010, extension to randomised pilot and feasibility trials, Explanation and Elaboration for important clarifications on all the items. If relevant, we also recommend reading CONSORT extensions for cluster randomised trials, non-inferiority and equivalence trials, non-pharmacological treatments, herbal interventions, and pragmatic trials. Additional extensions are forthcoming: for those and for up to date references relevant to this checklist, see www.consort-statement.org.

For peer review only

BMJ Open

The STEPFORWARD study: a randomised controlled feasibility trial of a self-aligning prosthetic ankle-foot for older patients with vascular-related amputations

Journal:	*BMJ Open*
Manuscript ID	bmjopen-2020-045195.R2
Article Type:	Original research
Date Submitted by the Author:	19-Jan-2021
Complete List of Authors:	Vanicek, Natalie; University of Hull, Sport, Health and Exercise Science<br>Coleman, Elizabeth; University of York, Health Sciences<br>Watson, Judith; University of York, Health Sciences<br>Bell, Kerry; University of York, Health Sciences<br>McDaid, Catriona; University of York, Health Sciences<br>Barnett, Cleveland; Nottingham Trent University, School of Science and Tecnology<br>Twiste, Martin; University of Salford<br>Jepson, Fergus; Lancashire Teaching Hospitals NHS Foundation Trust, Specialist Mobility Rehabilitation Centre<br>Salawu, Abayomi; Hull University Teaching Hospitals NHS Trust, Disability Medicine and Rehabilitation Unit<br>Harrison, Dennis<br>Mitchell, Natasha; University of York, York Trials Unit, Department of Health Sciences
**Primary Subject Heading**:	Rehabilitation medicine
Secondary Subject Heading:	Patient-centred medicine
Keywords:	REHABILITATION MEDICINE, Clinical trials < THERAPEUTICS, Foot & ankle < ORTHOPAEDIC & TRAUMA SURGERY, Musculoskeletal disorders < ORTHOPAEDIC & TRAUMA SURGERY

SCHOLARONE™
Manuscripts

I, the Submitting Author has the right to grant and does grant on behalf of all authors of the Work (as defined in the below author licence), an exclusive licence and/or a non-exclusive licence for contributions from authors who are: i) UK Crown employees; ii) where BMJ has agreed a CC-BY licence shall apply, and/or iii) in accordance with the terms applicable for US Federal Government officers or employees acting as part of their official duties; on a worldwide, perpetual, irrevocable, royalty-free basis to BMJ Publishing Group Ltd ("BMJ") its licensees and where the relevant Journal is co-owned by BMJ to the co-owners of the Journal, to publish the Work in this journal and any other BMJ products and to exploit all rights, as set out in our licence.

The Submitting Author accepts and understands that any supply made under these terms is made by BMJ to the Submitting Author unless you are acting as an employee on behalf of your employer or a postgraduate student of an affiliated institution which is paying any applicable article publishing charge ("APC") for Open Access articles. Where the Submitting Author wishes to make the Work available on an Open Access basis (and intends to pay the relevant APC), the terms of reuse of such Open Access shall be governed by a Creative Commons licence – details of these licences and which Creative Commons licence will apply to this Work are set out in our licence referred to above.

Other than as permitted in any relevant BMJ Author's Self Archiving Policies, I confirm this Work has not been accepted for publication elsewhere, is not being considered for publication elsewhere and does not duplicate material already published. I confirm all authors consent to publication of this Work and authorise the granting of this licence.

For peer review only

Title page:
The STEPFORWARD study: a randomised controlled feasibility trial of a self-aligning prosthetic ankle-foot for older patients with vascular-related amputations

Authors: Natalie Vanicek[1], Elizabeth Coleman[2], Judith Watson[2], Kerry Bell[2], Catriona McDaid[2], Cleveland Barnett[3], Martin Twiste[4], Fergus Jepson[5], Abayomi Salawu[6], Dennis Harrison[7] and Natasha Mitchell[2]

Corresponding author: Professor Natalie Vanicek
Department of Sport, Health and Exercise Science
Faculty of Health Sciences
University of Hull
Hull
HU6 7RX
E: n.vanicek@hull.ac.uk
T: 01482 463607

Author affiliations
[1] Department of Sport, Health and Exercise Science, University of Hull, Hull, UK
n.vanicek@hull.ac.uk Orcid iD 000-0002-9602-3172
[2] York Trials Unit, Department of Health Sciences, University of York, York, UK
izzy.coleman@york.ac.uk Orcid iD 0000-0003-4210-1865
jude.watson@york.ac.uk Orcid iD 0000-0003-0694-3854
kerry.bell@york.ac.uk Orcid iD 0000-0001-5124-138X
catriona.mcdaid@york.ac.uk Orcid iD 0000-0002-3751-7260
natasha.mitchell@york.ac.uk Orcid iD 0000-0002-6904-2976
[3] School of Science and Technology, Nottingham Trent University, Nottingham, UK
cleveland.barnett@ntu.ac.uk Orcid iD 0000-0001-6898-9095
[4] School of Health and Society, University of Salford, Manchester, UK
m.twiste@salford.ac.uk Orcid iD 0000-0003-2724-3283
[5] Specialist Mobility Rehabilitation Centre, Lancashire Teaching Hospitals NHS Foundation Trust, Preston, UK
Fergus.Jepson@lthtr.nhs.uk
[6] Disability Medicine and Rehabilitation Unit, Hull University Teaching Hospitals NHS Trust, Hull, UK
abayomi.salawu@hey.nhs.uk Orcid iD 0000-0003-2496-0826
[7] Public Involvement Member
dharrisonpublic@gmail.com

Keywords
Randomised controlled trial, feasibility, self-aligning, ankle-foot prosthesis, transtibial amputation, acceptability, vascular

Word count: 4,971 words

For peer review only - http://bmjopen.bmj.com/site/about/guidelines.xhtml

ABSTRACT

Objectives: To determine the feasibility of conducting a full-scale randomised controlled trial (RCT) of the effectiveness and cost-effectiveness of a self-aligning prosthetic ankle-foot compared with a standard prosthetic ankle-foot.

Design: Multi-centre parallel group feasibility RCT.

Setting: Five prosthetics centres in England recruiting from July 2018 to August 2019.

Participants: Adults aged ≥ 50 years with a vascular-related or non-traumatic transtibial amputation for one year or longer, categorised as having 'limited community mobility' and using a non-self-aligning ankle-foot.

Intervention: Participants were randomised into one of two groups for 12 weeks: self-aligning prosthetic ankle-foot or existing non-self-aligning prosthetic ankle-foot.

Outcomes: Feasibility measures: recruitment, consent and retention rates; and completeness of questionnaire and clinical assessment datasets across multiple time points. Feasibility of collecting daily activity data with wearable technology and health resource use data with a bespoke questionnaire.

Results: Fifty-five participants were randomised (61% of the target 90 participants): n=27 self-aligning ankle-foot group, n=28 non-self-aligning ankle-foot group. Fifty-one participants were included in the final analysis (71% of the target number of participants). The consent rate and retention at final follow-up were 86% and 93%, respectively. The average recruitment rate was 1.25 participants/site/month (95% CI 0.39 to 2.1). Completeness of questionnaires ranged from 89-94%, and clinical assessment were 92-95%, including the activity monitor data. The average completion rates for the EQ-5D-5L and bespoke resource use questionnaire were 93% and 63%, respectively.

Conclusions: This feasibility trial recruited and retained participants who were categorised as having 'limited community mobility' following a transtibial amputation. The high retention rate of 93% indicated the trial was acceptable to participants and feasible to deliver as a full-scale RCT. The findings support a future, fully-powered evaluation of the effectiveness and cost-effectiveness of a self-aligning prosthetic ankle-foot compared to a standard non-self-aligning version with some adjustments to the trial design and delivery.

Trial registration

ISRCTN15043643

For peer review only - http://bmjopen.bmj.com/site/about/guidelines.xhtml

ARTICLE SUMMARY

Strengths and limitations of this study

- This was one of few clinical trials investigating ankle-foot prostheses within prosthetics services in the UK. The study design is a multi-centre, randomised controlled trial in an under-researched clinical population involving prosthetics.
- The completeness of subjective and objective assessment measures, administered in a clinical setting and via post, was high.
- Feasibility and acceptability were ascertained with quantitative and trial procedure data.
- Recruitment was lower and slower than anticipated, but the ability to retain participants was high.
- Effectiveness and cost-effectiveness of a self-aligning ankle-foot will need to be established in a definitive full-scale trial.

For peer review only

For peer review only - http://bmjopen.bmj.com/site/about/guidelines.xhtml

INTRODUCTION

In the UK, there are approximately 6,000 new referrals to prosthetics services every year. Most of these referrals are patients aged over 50 years who have had an amputation at the transtibial (below-knee) level.[1] The majority of lower limb amputations result from long-term diabetes mellitus, coronary and peripheral vascular diseases.[2, 3] Consequently, most new referrals for a prosthesis involve older adults who have other health comorbidities and who are categorised as having 'limited community mobility'. These patients are an under-researched group, with few studies focused on their mobility with a prosthesis.

Many factors determine the type of prosthesis that a patient receives, such as estimation of patient outcomes and goals, and the healthcare budget.[4] A Cochrane Review reported there was insufficient evidence from robust studies to support the "overall superiority of any individual type of prosthetic ankle-foot mechanism".[5] There are also no standardised criteria for the prescription of ankle-foot prostheses. Practice varies across UK centres and is frequently cost-driven. Subsequently, the majority of older people with a transtibial amputation receive a standard, non-self-aligning ankle-foot, such as the non-articulated solid ankle cushioned heel (SACH), uniaxial or multiaxial prosthetic foot. These prosthetic ankle-feet have limited functionality. The SACH foot has a solid 'ankle' that does not allow any movement. The uniaxial ankle-foot is only capable of moving about a single axis with some ankle dorsi- and plantar-flexion; the multiaxial ankle-foot can rotate about multiple axes, allowing ankle dorsi- and plantar-flexion and eversion-inversion. The elastic recoil to return to a plantigrade position makes the uni- and multi-axial ankle-feet unable to self-align to inclined surfaces, making these feet more suitable for standing or walking on level ground. This requires the user to make compensatory actions at proximal joints (compensations at the hip bilaterally and intact knee of the affected limb) and at the intact ankle to achieve stability with a 'foot flat' position when walking on slopes.[6, 7] These actions result in greater asymmetry between the two limbs and increase the metabolic requirements of walking.[6] This can make walking more difficult and tiring. Our public involvement members reported that a poorly functioning prosthesis can contribute to sedentary behaviour, pain and more frequent visits to prosthetics centres and other healthcare services, which may lead to disuse and poorer quality-of-life. Therefore, identifying a more functionally suitable prosthesis could benefit patients in meaningful ways following amputation.

A 'family' of prosthetic ankle-feet that use a hydraulic mechanism to self-align to sloped surfaces have been designed to attenuate many of the burdens associated with standard, non-self-aligning ankle-feet. These prosthetic ankle-feet have been designed for users of varying ability (more active users, K3-K4 classification) and also for users categorised as having 'limited community mobility' (K2 users).[8] The hydraulic mechanism allows the prosthetic ankle-foot to self-align to sloped surfaces (due to ground reaction forces) and remain in a dorsiflexed position throughout the swing phase. This can improve ground

For peer review only - http://bmjopen.bmj.com/site/about/guidelines.xhtml

clearance and securing the biological knee, which is important for falls prevention.[9] Previous laboratory-based studies reported that a self-aligning ankle-foot reduced residuum-socket interface pressures in active users,[10] which could alleviate pain in the residuum longer-term, improved walking[11] and enhanced quality of life[12, 13] in users with lower activity levels. More functional prostheses may improve patient function and mobility following a lower limb amputation.[14] However, the effects of a self-aligning prosthesis have not been evaluated with robust randomised controlled trials (RCTs) in a community setting. In particular there is a lack of research involving participants who have had a non-traumatic (e.g., vascular-related) amputation. A self-aligning ankle-foot is more expensive than a non-self-aligning version by approximately £900 for the K2-user version (at 2020 UK prices) with variations in price across manufacturers. However, the potential patient benefits, such as improved mobility, better quality-of-life, and reduced falls, could offset future healthcare and socioeconomic costs.

There is a need for a RCT of the effectiveness and cost-effectiveness of a self-aligning prosthetic ankle-foot for older patients, with a vascular-related or non-traumatic transtibial amputation, compared to a standard, non-self-aligning ankle-foot. The STEPFORWARD study used a mixed-methods approach to determine feasibility of conducting such a study. A feasibility study was considered important in advance of a full-scale RCT given the lack of large community-based RCTs in this field. A full-scale trial will be powered appropriately to detect a difference in the primary outcome between the two trial arms (and as such has a sample size large enough to answer a research question on intervention effectiveness). A feasibility study addresses the question of whether a specific study can be done, whether it should proceed and, if so, how.[15] It is not designed to address the question of whether an intervention works. This article reports the feasibility as determined by the quantitative data, which was assessed according to: (1) participant recruitment, including time to recruit, consent and retention rates and adverse events; (2) completeness of data across multiple time points, including health economics data while piloting a bespoke health resource use questionnaire; and (3) day-to-day activity measured with wearable technology. The findings from this work could be relevant to clinical practitioners, researchers and policy makers.

METHODS

Study design and participants

The STEPFORWARD trial was a multi-centre, parallel group, randomised controlled feasibility trial to assess the possibility of conducting a full-scale RCT in the future, which would be adequately powered to answer the primary research question. The trial received favourable ethical approval from the National Health Service Yorkshire and the Humber—Leeds West Research Ethics Committee (ref:18/YH/0089) and Health Research Authority in May 2018. The trial was registered on the ISRCTN clinical trial registry (ISRCTN15043643). The full study protocol, with detailed inclusion/exclusion criteria and recruitment/screening pathways, has been published[16] and is briefly described below.

For peer review only - http://bmjopen.bmj.com/site/about/guidelines.xhtml

We aimed to recruit 90 participants who were aged ≥ 50 years with a unilateral transtibial amputation for ≥1 year due to vascular, neurological or life-limiting reasons, who were categorised as having 'limited community mobility', with a stable residuum, and using a non-self-aligning prosthetic ankle-foot (e.g., SACH, uniaxial, multiaxial (e.g., Multiflex) or other K1/K2 feet). We excluded anyone who had a contraindication to wearing their current prosthesis or the self-aligning ankle-foot (according to the manufacturer's specifications), had experienced a recent cerebrovascular event or had a disease affecting their memory. Allowing for 20% attrition, we anticipated 72 participants in our final analysis.

Recruitment and randomisation

We used a two-stage screening process to identify eligible patients; full details are provided in the published protocol.[16] In summary, potential participants were identified by database screening or during routine appointments to five prosthetics centres in England from July 2018 to August 2019. They were posted a study invitation pack and asked to return a Consent to Contact form if they were interested in being contacted about the trial. A member of their multidisciplinary team then contacted them and completed the first section of the Screening Form over the telephone. If potentially eligible, they were invited into clinic to complete the outstanding screening questions, which had to be done face-to-face, and provide their written consent. Following consent, eligible patients completed all baseline measures before being randomised into one of two trial arms: keep their non-self-aligning prosthetic ankle-foot (standard treatment group) or receive a new self-aligning ankle-foot (intervention group). Participants were randomised individually, stratified according to prosthetics centre on a 1:1 basis, by a telephone randomisation service set up by York Trials Unit. Blinding of the participants and investigators was not possible because of the nature of the intervention.

Standard treatment and intervention

Participants in the standard treatment group continued using their normal prosthetic ankle-foot and had access to all clinical services as normal. They were asked to carry on with their normal daily routine during the intervention period.

Participants in the intervention group were fitted with the self-aligning prosthetic ankle-foot by their regular prosthetist. The self-aligning ankle-foot used in this trial was the Avalon^{K2}, manufactured by Blatchford, Basingstoke, UK (Patent reg: 5336386), which was already commercially available and could be prescribed under the national health service (NHS). Once fitted, participants were asked to ambulate with it as they would normally with any new prosthetic ankle-foot for approximately 12 weeks after fitting (intervention period). At the end of the study, participants in the intervention group only could keep the self-aligning ankle-foot if they preferred it. Participants in standard treatment group were not offered

For peer review only - http://bmjopen.bmj.com/site/about/guidelines.xhtml

the self-aligning ankle-foot at the end of the study as it is not known whether it is better or more acceptable to users than the standard prosthesis.

Outcome measures

The primary outcome of this feasibility study related to collecting data on recruitment, consent and retention rates. Reasons for non-participation or withdrawal were recorded where possible. We assessed the completeness of data across multiple time points: baseline, interim and final follow-ups. The baseline and final assessments were conducted at the participant's usual prosthetics centre. At the interim follow-up, only questionnaire data were collected via post. Data were gathered from questionnaires (baseline demographics and bespoke health resource use questionnaires, the Locomotor Capabilities Index (LCI-5),[17] Houghton Scale,[18] the Patient-Reported Outcomes Measurement Information System (PROMIS) Short-Form v1.0 Pain (3a and 8a) questionnaires[19] and EQ-5D-5L[20]) and from clinical assessments (Timed Up and Go (TUG) test,[21] Timed Up and Down Stairs (TUDS) test,[22] 2-minute walk test (2mWT)[23] and Berg Balance Scale (BBS)[24]). Data related to daily step count and time spent walking were collected from a loaned activity monitor (activPAL4™, PAL Technologies Ltd, Glasgow, UK) worn on the prosthesis for one week. At baseline, all participants wore their normal (non-self-aligning) prosthetic ankle-foot during the clinical assessments and activity monitor data collection; at final follow-up they wore whichever prosthetic ankle-foot they were using currently. Adverse events (AEs) related to the prosthesis only, and any serious adverse events (SAEs), were recorded throughout the study.

Statistical analyses

The recruitment rate was reported monthly, and overall, by site. An average monthly recruitment rate was calculated, with a 95% confidence interval (CI) estimated from the data collected. The number of eligible patients and those approached for consent was summarised overall, by site, using counts and percentages.

Baseline data were summarised by trial arm, as randomised, with no formal comparison between the groups. Continuous data were reported descriptively and categorical data by counts and percentages. Completion rates of all the clinical outcome measures were reported by trial arm and overall.

Health economic analysis

The health economics work investigated the feasibility of collecting self-reported patient use of health service resources (e.g., primary care visits, prescription medication) over the study period and to evaluate the appropriateness of these data. This information would help to refine the bespoke questionnaire in a future trial. A full cost-effectiveness analysis was not completed as this was a feasibility study. Nonetheless, the costing approach was undertaken from an NHS perspective; unit costs were derived from established national costing sources

For peer review only - http://bmjopen.bmj.com/site/about/guidelines.xhtml

such as NHS Reference Costs[25] and Personal Social Services Research Unit costs of health and social care.[26]

RESULTS

Feasibility assessment

Recruitment

Over 880 potential participants were initially screened by searching patient databases across the five prosthetics centres. Seventy-eight were identified as potentially eligible after the first screening, but 12 were subsequently excluded following the final, second screening. Eighty-six percent of those screened, who were found to be fully eligible, consented to participating (55 of 64 participants) in the trial. The denominator was 64 as two were randomised in error and subsequently withdrawn (Figure 1). As we used a two-stage screening process, only potential participants who met the initial inclusion criteria were approached; those uninterested in being involved during the initial screening were not screened further. Therefore, the consent rate may be artificially high. Reasons for non-participation are also presented in Figure 1. In total, 55 participants (61% of the target 90 participants) were included and randomised as follows: 27 to the self-aligning prosthetic ankle-foot (intervention) group and 28 to the non-self-aligning (standard treatment) group.

Initial recruitment was slower than anticipated, therefore two additional recruiting sites were included midway through the trial, bringing the total number of participating sites to five. The average recruitment rate was 1.25 participants/site/month (95% CI 0.39 to 2.1) ranging from 0.83 to 2.2 participants per month. Each site recruited approximately the same number of participants (mean=11, range=9-13 participants), despite them being open to recruitment for different lengths of time (range=5.0-13.7 months).

Participants

The mean (SD) age of participants in this study was 68.8 (9.6) years, ranging from 51.8 to 86.8 years. Most participants were male (85.5%) and all classified themselves as White British, Irish or other. The most common reason for amputation was diabetes (45.5%). Full participant characteristics are described in Table 1.

Follow-up and adverse events

In addition to the two people randomised in error and withdrawn, a further two participants who were allocated to the intervention group withdrew from the study: one as a result of having suffered a stroke and one reported the self-aligning prosthetic ankle-foot was too heavy. At final follow-up, 51 of the 55 participants returned the questionnaires giving a retention rate of 93%. This was 71% of the planned target of 72 participants included in the final analysis.

For peer review only

For peer review only - http://bmjopen.bmj.com/site/about/guidelines.xhtml

A total of four SAEs and one AE were reported. The SAEs were due to the following reasons: stroke; fall and femoral fracture when not wearing their prosthesis (both of these cases occurred in the intervention group); fall and hip fracture, leading to hospitalisation and subsequently death; and recurrent falls with fibular fracture (both of these cases were in the standard treatment group). The AE occurred because one participant (intervention group) developed blistering on their residuum.

Data completeness

There were four standardised measures completed at all three time points: LCI-5, Houghton, PROMIS 3A and PROMIS 8A. The average completion rate across all time points ranged from 89-94%. Data from four clinical assessments (TUG, TUDS, 2mWT and BBS), and an activity monitor worn for a week, were collected at both baseline and final follow-up, and their average completion rate ranged from 92-95%. All the measures were well completed, or attempted, with at least 75% completed in full. Details of the outcome measures and completion rates can be found in Tables 2 and 3.

None of the four standardised measures appeared to show a change between baseline and follow-up. For the 2-minute walk test, participants with the self-aligning prosthetic ankle-foot walked on average further at final follow-up (mean (SD): 88.8 (40.0) metres) than at baseline (81.0 (31.4) metres). This equates to an average increase of 6.2 (16.2) metres in walking distance for the participants in the intervention group between these two time points. However, in the standard treatment group, there was an average decrease of 9.0 (29.8) metres, indicating that these participants were walking less distance over two minutes at the final follow-up. Scores for the TUG, TUDS and BBS remained largely unchanged across both groups. Activity monitor data revealed that the daily time spent stepping between baseline and follow-up was mostly unchanged for both groups, which was approximately 30 minutes per day. Conversely, the number of steps taken per day decreased in the intervention group (1755.5 (1963.7) steps at baseline vs. 1673.2 (1594.4) steps at final follow-up) yet increased in the standard treatment group (1750.9 (1369.9) steps at baseline vs. 1836.1 (1647.8) steps at final follow-up). The average change in number of steps taken per day decreased by 2.9 (756.2) steps in the intervention group between baseline and final follow-up, yet increased by 283.3 (1614.8) steps in the standard treatment group. In both groups, the variability in daily stepping was high.

Health economic data

The health economic outcomes are based on the patient-completed questionnaires, which incorporated the EQ-5D-5L and a bespoke resource use questionnaire. The average completion rates were 93% and 63%, respectively. Over 89% of participants completed the EQ-5D-5L fully. At baseline, responses to the EQ-5D-5L indicated relatively equal levels of health utility between the trial arms. By final follow-up, an increase in mean utility of 0.13 in the intervention group was observed, whilst mean utility in the standard treatment group

For peer review only - http://bmjopen.bmj.com/site/about/guidelines.xhtml

remained the same. A positive mean difference in favour of the self-aligning prosthetic ankle-foot in the intervention group remained consistent after adjusting for baseline utility (Table 4).

For the resource use questionnaire, the completion rates did not differ by the respective components, suggesting all sections were acceptable and each type of data was feasible to collect. Resource use was minimal across both primary and secondary/tertiary care for participants in both arms of the trial. The most frequently received care was via outpatient attendances at NHS hospitals (mean (SD): 0.21 (0.51) rising to 2.04 (3.72) visits from baseline to final follow-up for the intervention group; 0.08 (0.27) rising to 1.14 (2.19) visits for the standard treatment group.

Medications were grouped according to comorbidity to investigate their inclusion in a full-scale trial as follows: blood pressure/heart problems, neuropathic pain, diabetes, phantom pain. Participant uptake of the groups of medications explored using the resource use questionnaire was high, and there were some differences between groups, suggesting a micro-costing exercise including medications data may be merited in a full-scale trial. Approximately 73% of participants overall reported taking medications related to blood pressure and/or heart problems, and 62% of participants overall took medications for diabetes. Medication use appeared to increase over the course of the trial for participants in both arms.

The intervention (self-aligning prosthetic ankle-foot) was delivered in the NHS setting by the usual staff providing post-amputation aftercare (prosthetist, physiotherapist). Other than the self-aligning ankle-foot itself, participants received equal care to standard treatment. The cost (at 2020 UK prices) of the self-aligning ankle-foot used in the trial was £1192 compared to the non-self-aligning version estimated at £287 (although this depends on the exact standard ankle-foot and would need to be determined accurately based on the user's actual foot). The additional intervention cost to the NHS of delivering the self-aligning ankle-foot is therefore approximately £905.

Identification of primary outcome measure

The findings from this study have demonstrated feasibility, acceptability and a signal of efficacy for three measures: walking ability with the 2mWT, daily activity with activity monitor data, and quality-of-life with the EQ-5D-5L. We consulted with our patient advisory group (PAG) to inform the appropriateness of a primary outcome to be used in a future full-scale trial. The PAG members reported that, of the three measures, the EQ-5D-5L resonated most strongly with them as it captured the constructs that were important to them (e.g., mobility, pain, usual activities, general health). This measure will be considered for use as the primary outcome in a future trial.

For peer review only - http://bmjopen.bmj.com/site/about/guidelines.xhtml

DISCUSSION

Main findings

The primary objective of this study was to determine the feasibility of conducting a future, full-scale RCT of the effectiveness and cost-effectiveness of a self-aligning prosthetic ankle-foot compared to a standard prosthetic ankle-foot. There are few clinical trials involving older adults living with a lower limb amputation, who are an under-researched but growing group of patients. The consent, retention and completion rates were high, demonstrating that it is feasible to recruit and retain participants to a future trial addressing the research question. While the consent rate may be artificially high, due to screening methods used in the current study, the final retention and completion rates indicate the study was acceptable to participants.

We did not meet the recruitment target of 90. Given that only 7.4% of screened patients met the eligibility criteria (though only 7.2% were truly eligible, due to the randomisation in error), it is important to consider whether the criteria were too restrictive and should be adjusted in a future trial. It is common that the residuum undergoes considerable volume changes within the first year post-amputation, necessitating the fabrication of new prosthetic sockets. As the intervention period of this feasibility study was only 12 weeks long (due to resource constraints), we sought to recruit established prosthesis users who had no current contraindications to wearing their ankle-foot prosthesis, had a stable residuum volume and the same prosthetic socket for ≥ 3 months and were free of open wounds or infections on the residuum. We also only included patients who had a transtibial amputation secondary to a health condition, as this group is most likely categorised as having 'limited community mobility' and frequently prescribed a non-self-aligning prosthetic ankle-foot. It would be clinically relevant to broaden the eligibility criteria to include any patient with a transtibial amputation within this mobility category, regardless of cause and time since amputation, in a future study. This would widen the pool of patients potentially eligible for the trial. However, we do not have data from the feasibility study to assess impact on recruitment and it would be important for the future trial to have an internal pilot with clear stop-go criteria to confirm the feasibility of a modified study design.

Five AEs, of which four were categorised as serious, occurred throughout this trial. All of the SAEs (one stroke and three falls) were not deemed related to the self-aligning prosthetic ankle-foot. This clinical group is vulnerable to falling, with one recent study stating that over 57% of community-living lower limb prosthesis users fall at least once a year[27] and that 36-75% of fallers experience recurrent falls.[27, 28] The participant who developed blistering on the residuum (the one AE) had been allocated to the intervention group. It is not uncommon for blistering to occur if the skin on the residuum is subjected to increased friction as a result of wearing the prosthesis for longer. In most instances, blisters heal relatively quickly, and the prosthesis user can soon return to normal function with their prosthesis.

For peer review only - http://bmjopen.bmj.com/site/about/guidelines.xhtml

Results in context with other research

A few randomised trials of several different prosthetic ankle-feet (although not self-aligning), evaluating function and self-report measures, have been conducted previously.[29-31] Some of these studies lacked sample size calculations and presented small samples (range n=10-27 participants/trial). Moreover, they represented a heterogeneous group in terms of their mobility level classifications (participants were mostly categorised as 'unlimited community ambulators' (K3) or 'active adults' (K4)), cause of amputation and health comorbidities. The age of the participants in these studies was also younger than in the current feasibility trial (mean range 42.3-57 years). Therefore, the findings from existing studies are difficult to generalise to the majority of people with a lower limb amputation due to dysvascularity, in the UK and other developed nations. We are not aware of any randomised trials investigating prosthetic ankle-feet uniquely involving older adults classified as having 'limited community mobility' as a result of health-related issues.

Outcome measures

This study was not designed to assess the effectiveness of the self-aligning prosthetic ankle-foot, and no formal comparisons between randomised groups were undertaken as the study was not powered to report effects. Three measures indicated a signal of potential efficacy including the 2-minute walk test (walking ability), daily activity as measured with activity monitors, and quality-of-life as measured with the EQ-5D-5L. Conversely, other measures (including some population-specific measures and common clinical assessments) did not indicate any difference.

We demonstrated that the use of activity monitors, and their implementation using postal delivery, was feasible in this patient group. We believe this was supported by our study procedures, which included regular participant information letters alerting them to the next stages of the trial, and a detailed bespoke leaflet explaining how participants were requested to fit the activity monitor onto the prosthesis themselves. As the monitor was affixed to the prosthesis, there were few issues with discomfort or discontinued use. Trials using subjective outcomes, where the outcome measure is self-reported by a participant or assessment of an event requires exercise of judgement by an observer, may overestimate the treatment effect.[32] Therefore, it was important to establish the feasibility of using this more objective outcome in a future trial.

On average, both groups took fewer than 1800 daily steps over 30 minutes throughout the day. The data related to daily steps and stepping time reinforced the largely sedentary behaviour experienced by this clinical group. Our findings are broadly similar to a previous study that reported an average of 1450 (SD 1309) steps/day in a group of older individuals (age: 64 (SD 9) years) with a predominantly transtibial amputation (95% of total participants) as a result of vascular disease when measured over 10 days.[33] Further research

For peer review only - http://bmjopen.bmj.com/site/about/guidelines.xhtml

is warranted to ascertain whether a more functional prosthetic ankle-foot, compared to current standard care with a non-self-aligning ankle-foot, has the long-term potential to increase daily stepping, which could have important implications for patient benefit.

Health economics

It was feasible to collect the data required for a full-scale trial economic evaluation from this patient population. The high levels of completion suggest acceptability of instruments. The EQ-5D-5L is likely to be sensitive to changes in health states over time. Some changes to the bespoke health resource use questionnaire could be made in future. Use of privately funded healthcare services was negligible so this section could potentially be omitted in a full-scale trial. Participant uptake of the medications in the resource use questionnaire was high and there were some differences between groups suggesting a micro-costing exercise including medications data may be merited in a full-scale trial. Although no resource use of walking aids, adaptations and accessories had an uptake greater than 20%, participants were established prosthesis users who may have already accessed the required adaptations prior to entering the trial. Therefore, if a future trial had broader eligibility criteria to include unestablished prosthesis users, it may be important to retain this item.

The approximate NHS cost of the intervention is ~£900 above current standard care as it is assumed that the self-aligning prosthetic ankle-foot is delivered in the usual NHS setting and by the usual staff. There are various self-aligning prosthetic ankle-feet available commercially (e.g., using hydraulic mechanisms vs. micro-processor controlled and with actively powered propulsion), at varying costs and suitability (e.g., some prosthetic ankle-feet are heavier and more suitable for different patient needs). At the present time, we have only considered a single prosthetic ankle-foot, but one that is designed for our clinical population of interest. A full health economics evaluation, as part of full-scale trial, would be able to evaluate whether the initial higher cost of the ankle-foot could be offset by reduced use of other health resources (e.g., medications, prosthetic adjustments) and/or increased patient quality-of-life.

Strengths and weaknesses

Prosthetics services are an under-researched area within the NHS. As this was one of few clinical trials conducted in this area in the UK, we now have a better understanding of the challenges of recruiting in this setting, such as the limited research resources at clinical sites and variation in how sites organise their patient database systems. The recruiting sites in England provided a wide geographical spread (North East, North West, East of England, Midlands and South of England); however, the participants recruited across these sites were homogenous in their ethnicity (self-reported as White British, Irish or other). In future, we will develop strategies to maximise participation from patients across different ethnic groups and nations in the United Kingdom.

For peer review only - http://bmjopen.bmj.com/site/about/guidelines.xhtml

The majority of participants were recruited after a member of their clinical team screened the patient database for potentially eligible participants and posted them a study invitation pack. Therefore, the majority of the screening forms (79%) were initially completed over the phone before the face-to-face screening process confirmed their final eligibility. A limitation of this feasibility study was our lack of prior knowledge of the management of patient databases, which varied across the sites. Four sites used an electronic database for patient records while one used a paper-based system only; one of these sites used three different patient record systems. Not all patient records were up-to-date, resulting in two participants being randomised in error because they did not fulfil the inclusion criteria fully, and they were subsequently withdrawn. In a future trial, it will be essential to gather relevant information about the organisation of patient records at the local participating sites prior to opening that site to facilitate effective recruitment. This would also help sites recruit participants more consistently across the recruitment period. We were concerned at the outset about the risk of 'resentful demoralisation' amongst participants allocated to usual care and were mindful of this in developing the patient information for the study. The fact that adherence to the study processes and completion of outcome measures were similar across groups provides some reassurance in this regard. However, this would need continuing attention in the design of a full-scale trial.

For peer review only - http://bmjopen.bmj.com/site/about/guidelines.xhtml

Future research

Based on the findings from this feasibility study, we propose some adaptations to the study protocol for a future full-scale trial. They include:

- broadening the participant eligibility criteria to include new patients with a transtibial amputation, who would be categorised as having 'limited community mobility' by their multi-disciplinary team, and therefore likely to receive a non-self-aligning prosthetic ankle-foot;
- lengthening the intervention period of accommodating to the self-aligning ankle-foot, and including a longer follow-up period, would be important for unestablished prosthesis users if the criteria were more inclusive in a full-scale trial;
- refining the screening process to screen more potentially eligible participants, even if some prove ineligible afterwards, with advanced information about site patient databases;
- reviewing the secondary outcome measures;
- redesigning some aspects of the health resource use questionnaire; and
- approaching other prosthetics centres nationally to explore their willingness to be involved in a full-scale trial.

CONCLUSION

Although we did not reach our target sample, due to slower than anticipated recruitment, 71% of expected participants were in the final analysis, owing to our retention rate of 93%. Therefore, we believe the STEPFORWARD study demonstrated feasibility to recruit and retain participants and the ability to obtain a variety of complete datasets collected in clinic and via post. We have also evidenced the ability to deliver the trial across multiple prosthetics centres in England. The knowledge gained and lessons learned can be implemented in a future definitive trial to determine the effectiveness and cost-effectiveness of a self-aligning prosthetic ankle-foot compared to a non-self-aligning version for people with a transtibial amputation and categorised as having 'limited community mobility'.

FIGURE CAPTION

Figure 1: CONSORT flowchart for the STEPFORWARD study

For peer review only - http://bmjopen.bmj.com/site/about/guidelines.xhtml

REFERENCES

1. University of Salford. LIMBLESS STATISTICS: Repository for quantitative information on the UK limbless population REFERRED for prosthetics treatment, Annual report 2011-2012. University of Salford; 2013.

2. Davies M, Burdett L, Bowling F, Ahmad N, McClennon J. The epidemiology of major lower-limb amputation in England: a systematic review highlighting methodological differences of reported trials. Diabet Foot J. 2019;22:53-61.

3. Crawford F, Cezard G, Chappell FM, Murray GD, Price JF, Sheikh A, et al. A systematic review and individual patient data meta-analysis of prognostic factors for foot ulceration in people with diabetes: the international research collaboration for the prediction of diabetic foot ulcerations (PODUS). Health Technol Assess. 2015;19(57).

4. Sansam K, O'Connor RJ, Neumann V, Bhakta B. Clinicians' perspectives on decision making in lower limb amputee rehabilitation. J Rehabil Med. 2014;46(5):447-53.

5. Hofstad C, Linde H, Limbeek J, Postema K. Prescription of prosthetic ankle-foot mechanisms after lower limb amputation. Cochrane Database Syst Rev. 2004(1):CD003978.

6. Askew GN, McFarlane LA, Minetti AE, Buckley JG. Energy cost of ambulation in trans-tibial amputees using a dynamic-response foot with hydraulic versus rigid 'ankle': insights from body centre of mass dynamics. J Neuroeng Rehabil. 2019;16(39).

7. Vrieling AH, van Keeken HG, Schoppen T, Otten E, Halbertsma JPK, Hof AL, et al. Uphill and downhill walking in unilateral lower limb amputees. Gait Posture. 2008; 28(2):235-242.

8. Agrawal V, Gailey RS, Gaunaurd IA, O'Toole C, Finnieston A, Tolchin R. Comparison of four different categories of prosthetic feet during ramp ambulation in unilateral transtibial amputees. Prosthet Orthot Int. 2015;39(5):380-9.

9. Johnson L, De Asha AR, Munjal R, Kulkarni J, Buckley JG. Toe clearance when walking in people with unilateral transtibial amputation: Effects of passive hydraulic ankle. J Rehabil Res Dev. 2014;51(3):429-37.

10. De Asha AR, Johnson L, Munjal R, Kulkarni J, Buckley JG. Attenuation of centre-of-pressure trajectory fluctuations under the prosthetic foot when using an articulating hydraulic ankle attachment compared to fixed attachment. Clin Biomech. 2013;28(2):218-24.

11. Barnett CT, Brown OH, Bisele MM, Brown MJ, De Asha AR, Strutzenberger G. Individuals with unilateral transtibial amputation and lower activity levels walk more quickly when using a hydraulically articulating versus rigidly attached prosthetic ankle-foot device. J Prosthet Orthot. 2018;30(3):158-64.

12. Moore R. Effect on stance phase timing asymmetry in individuals with amputation using hydraulic ankle units. J Prosthet Orthot. 2016;28:44-8.

13. Moore R. Patient evaluation of a novel prosthetic foot with hydraulic ankle aimed at persons with amputation with lower activity levels. J Prosthet Orthot. 2017;29:44-7.

For peer review only - http://bmjopen.bmj.com/site/about/guidelines.xhtml

14. Goujon H, Bonnet X, Sautreuil P, Maurisset M, Darmon L, Fode P, et al. A functional evaluation of prosthetic foot kinematics during lower-limb amputee gait. Prosthet Orthot Int. 2006;30(2):213-23.

15. Eldridge SM, Lancaster GA, Campbell MJ, Thabane L, Hopewell S, Coleman CL, et al. Defining feasibility and pilot studies in preparation for randomised controlled trials: Development of a conceptual framework. PLoS ONE. 2016;11(3):e0150205.

16. Mitchell N, Coleman E, Watson J, Bell K, McDaid C, Barnett C, et al. Self-aligning prosthetic device for older patients with vascular-related amputations: protocol for a randomised feasibility study (the STEPFORWARD study). BMJ Open. 2019;9(9):e032924.

17. Franchignoni F, Orlandini D, Ferriero G, Moscato TA. Reliability, validity, and responsiveness of the locomotor capabilities index in adults with lower-limb amputation undergoing prosthetic training. Arch Phys Med Rehabil. 2004;85(5):743-8.

18. Devlin M, Pauley T, Head K, Garfinkel S. Houghton Scale of prosthetic use in people with lower extremity amputations: Reliability, validity, and responsiveness to change. Arch Phys Med Rehabil. 2004;85(8):1339-44.

19. Morgan SJ, Friedly JL, Amtmann D, Salem R, Hafner BJ. Cross-sectional assessment of factors related to pain ontensity and pain interference in lower limb prosthesis users. Arch Phys Med Rehabil. 2017;98(1):105-13.

20. Hurst NP, Kind P, Ruta D, Hunter M, Stubbings A. Measuring health-related quality of life in rheumatoid arthritis: Validity, responsiveness and reliability of EuroQol (EQ-5D). Br J Rheumatol. 1997;36(5):551-9.

21. Podsiadlo D, Richardson S. The timed "Up & Go": A test of basic functional mobility for frail elderly persons. J Am Geriatr Soc. 1991;39(2):142-8.

22. Bonnyaud C, Zory R, Pradon D, Vuillerme N, Roche N. Clinical and biomechanical factors which predict timed up and down stairs test performance in hemiparetic patients. Gait Posture. 2013;38(3):466-70.

23. Butland RJA, Pang J, Gross ER, Woodcock AA, Geddes DM. Two-,six-, and 12-minute walking tests in respiratory-disease. Br Med J. 1982;284(6329):1607-8.

24. Major MJ, Fatone S, Roth EJ. Validity and reliability of the Berg Balance Scale for community-dwelling persons with lower-limb amputation. Arch Phys Med Rehabil. 2013;94(11):2194-202.

25. Department of Health. NHS reference costs 2017/18. London. 2018 [Available from: https://improvement.nhs.uk/resources/reference-costs/#rc1718.

26. Curtis L, Burns A. Unit Costs of Health and Social Care 2016. Personal Social Services Research Unit: University of Kent; 2016.

27. Kim J, Major MJ, Hafner B, Sawers A. Frequency and circumstances of falls reported by ambulatory unilateral lower limb prosthesis users: A secondary analysis. PM R. 2019;11(4):344-53.

For peer review only - http://bmjopen.bmj.com/site/about/guidelines.xhtml

28. Miller WC, Speechley M, Deathe B. The prevalence and risk factors of falling and fear of falling among lower extremity amputees. Arch Phys Med Rehabil. 2001;82(8):1031-7.

29. Gailey RS, Gaunaurd I, Agrawal V, Finnieston A, O'Toole C, Tolchin R. Application of self-report and performance-based outcome measures to determine functional differences between four categories of prosthetic feet. J Rehabil Res Dev. 2012;49(4):597-612.

30. Morgan SJ, McDonald CL, Halsne EG, Cheever SM, Salem R, Kramer PA, Hafner BJ. Laboratory- and community-based health outcomes in people with transtibial amputation using crossover and energy-storing prosthetic feet: A randomized crossover trial. PLoS ONE. 2018;13(2): e0189652.

31. Raschke SU, Orendurff MS, Mattie JL, Kenyon DEA, Jones OY, Moe D, Winder L, Wong AS, Moreno-Hernandez A, Highsmith MJ, Sanderson DJ, Kobayashi T. Biomechanical characteristics, patient preference and activity level with different prosthetic feet: A randomized double blind trial with laboratory and community testing. J Biomech. 2015;48(1):146-52.

32. Wood L, Egger M, Gluud LL, Schulz KE, Juni P, Altman DG, Gluud C, Martin RM, Wood AJG, Sterne JAC. Empirical evidence of bias in treatment effect estimates in controlled trials with different interventions and outcomes: meta-epidemiological study. Br Med J. 2008;336(7644):601-5.

33. Miller MJ, Cook PF, Kline PW, Anderson CB, Stevens-Lapsley JE, Christiansen CL. Physical function and pre-amputation characteristics explain daily step count after dysvascular amputation. PM R. 2019;11(10):1050-8.

For peer review only

For peer review only - http://bmjopen.bmj.com/site/about/guidelines.xhtml

Table 1: Demographic characteristics according to trial arm

	Self-aligning (n=27)	Standard (n=28)	Overall (n=55)
**Age, years**	N=27	N=28	N=55
Mean (SD)	70.0 (9.2)	67.6 (10.0)	68.8 (9.6)
Median (min, max)	71.5 (52.7, 86.8)	68.3 (51.8, 84.5)	70.7 (51.8, 86.8)
**Gender, n(%)**			
Male	23 (85.2)	24 (85.7)	47 (85.5)
Female	4 (14.8)	4 (14.3)	8 (14.6)
**Ethnicity, n(%)**			
White (British, Irish or Other)	27 (100.0)	28 (100.0)	55 (100.0)
Black (African, Caribbean or Other)	0 (0.0)	0 (0.0)	0 (0.0)
Asian (Indian, Pakistani, Bangladeshi or Other)	0 (0.0)	0 (0.0)	0 (0.0)
Mixed Background	0 (0.0)	0 (0.0)	0 (0.0)
Chinese	0 (0.0)	0 (0.0)	0 (0.0)
**Marital status, n(%)**			
Living alone never married	2 (7.4)	3 (10.7)	5 (9.1)
Living with partner	4 (14.8)	0 (0.0)	4 (7.3)
Married/civil partnership	9 (33.3)	19 (67.9)	28 (50.9)
Separated	2 (7.4)	1 (3.6)	3 (5.5)
Divorced	5 (18.5)	4 (14.3)	9 (16.4)
Widowed	2 (7.4)	1 (3.6)	3 (5.5)
Missing	3 (11.1)	0 (0.0)	3 (5.5)
**Main activity, n(%)**			
Full-time employment	1 (3.7)	3 (10.7)	4 (7.3)
Part-time employment	0 (0.0)	1 (3.6)	1 (1.8)
Self-employed	0 (0.0)	0 (0.0)	0 (0.0)
Unable to work due to poor health	1 (3.7)	5 (17.9)	6 (10.9)
Unemployed	0 (0.0)	1 (3.6)	1 (1.8)
Retired	24 (88.9)	17 (60.7)	41 (74.6)
Other	1 (3.7)	1 (3.6)	2 (3.6)
**Smoking status, n(%)**			
Ex-smoker	15 (55.6)	17 (60.7)	32 (58.2)
Current smoker	1 (3.7)	3 (10.7)	4 (7.3)
Never smoked	11 (40.7)	8 (28.6)	19 (34.6)
**Diabetes status, n(%)**			
Yes	19 (70.4)	18 (64.3)	37 (67.3)
No	8 (29.6)	10 (35.7)	18 (32.7)
**If yes:**			
Type one	5 (26.3)	2 (11.1)	7 (18.9)
Type two	12 (63.2)	13 (72.2)	25 (67.8)
Missing	2 (10.5)	3 (16.7)	5 (13.5)
**Cause of amputation, n(%)[a]**			
Diabetes	14 (51.9)	11 (39.3)	25 (45.5)
Peripheral vascular disease	10 (37.0)	10 (35.7)	20 (36.4)
Blood clot	3 (11.1)	2 (7.1)	5 (9.1)

For peer review only - http://bmjopen.bmj.com/site/about/guidelines.xhtml

Deep vein thrombosis	1 (3.7)	0 (0.0)	1 (1.8)
Aneurysm	0 (0.0)	1 (3.6)	1 (1.8)
Diabetic neuropathy	2 (7.4)	1 (3.6)	3 (5.5)
Other neuropathy	1 (3.7)	0 (0.0)	1 (1.8)
Cancer	1 (3.7)	1 (3.6)	2 (3.6)
Other	5 (18.5)	6 (21.4)	11 (20.0)

[a] Multiple options could be selected

For peer review only

For peer review only - http://bmjopen.bmj.com/site/about/guidelines.xhtml

Table 2. Standard outcomes at each time point (baseline, interim and final follow-up), by trial arm and overall

	Self-aligning (n=27)	Standard (n=28)	Overall (n=55)
**LCI-5 overall score (0 – 56): average completion 94%**			
**Baseline**	N=25	N=28	N=53
Mean (SD)	36.4 (12.0)	39.6 (11.2)	39.1 (11.6)
Median (p25, p75)	38 (30.3, 46)	38 (31, 50)	38 (30.3, 47)
**Interim**	N=24	N=25	N=49
Mean (SD)	37.3 (12.3)	38.5 (10.8)	37.9 (11.4)
Median (p25, p75)	39.5 (28.5, 47.5)	41 (30, 44.7)	41 (29, 46)
**Final**	N=25	N=26	N=51
Mean (SD)	37.8 (11.4)	39.2 (11.2)	38.5 (11.2)
Median (p25, p75)	40 (30, 47)	40.5 (29, 49)	40 (29, 47)
**Houghton overall score (0 – 12): average completion: 89%**			
**Baseline**	N=24	N=28	N=52
Mean (SD)	7.8 (2.0)	8.8 (1.8)	8.3 (1.9)
Median (p25, p75)	8 (7.5, 9)	9 (8, 10)	9 (8, 9)
**Interim**	N=20	N=23	N=43
Mean (SD)	8.4 (2.0)	8.5 (1.3)	8.5 (1.6)
Median (p25, p75)	9 (7, 10)	9 (8, 9)	9 (8, 10)
**Final**	N=23	N=26	N=49
Mean (SD)	8.3 (1.9)	8.5 (2.0)	8.4 (1.9)
Median (p25, p75)	8 (8, 10)	9 (8, 9)	9 (8, 9)
**PROMIS 3a overall score (30.7 – 71.8): average completion: 90%**			
**Baseline**	N=27	N=27	N=54
Mean (SD)	41.3 (10.0)	43.1 (9.5)	42.2 (9.7)
Median (p25, p75)	43.5 (30.7, 52.1)	43.5 (30.7, 49.4)	43.5 (30.7, 49.4)
**Interim**	N=21	N=25	N=46
Mean (SD)	43.6 (9.6)	45.6 (10.3)	44.7 (10.0)
Median (p25, p75)	43.5 (30.7, 49.4)	46.3 (40.2, 52.1)	46.3 (30.7, 52.1)
**Final**	N=24	N=22	N=46
Mean (SD)	40.4 (9.4)	42.7 (8.7)	41.4 (9.0)
Median (p25, p75)	40.2 (30.7, 47.9)	44.9 (30.7, 49.4)	43.5 (30.7, 49.4)
**PROMIS 8a overall score (40.7 – 77.0): average completion: 94%**			
**Baseline**	N=27	N=28	N=55
Mean (SD)	52.8 (11.2)	51.7 (9.4)	52.2 (10.2)
Median (p25, p75)	55 (40.7, 63.5)	51.8 (40.7, 58.5)	52.3 (40.7, 60.2)
**Interim**	N=23	N=24	N=47
Mean (SD)	53.5 (11.3)	56.7 (8.9)	55.1 (10.1)
Median (p25, p75)	55 (40.7, 64.1)	58.8 (52.3, 63.2)	57.4 (40.7, 63.5)
**Final**	N=25	N=26	N=51
Mean (SD)	51.2 (11.4)	53.3 (10.0)	52.3 (10.7)
Median (p25, p75)	49.9 (40.7, 60.2)	54.5 (40.7, 60.2)	53.2 (40.7, 60.2)

LCI-5 (Locomotor Capabilities Index-5): higher scores indicate a greater locomotor capability with the prosthesis; Houghton: higher scores indicate greater performance and comfort; PROMIS (Patient-Reported Outcomes Measurement Information System) 3a (pain intensity): coverts raw scores to T-scores, higher scores indicate more intense pain; PROMIS 8a: higher scores indicate more pain interference.

For peer review only - http://bmjopen.bmj.com/site/about/guidelines.xhtml

Table 3. Clinical assessments (TUG, TUDS, 2mWT and BBS) at baseline and final follow-up, by trial arm and overall

	Self-aligning (n=27)	Standard (n=28)	Overall (n=55)
**TUG, seconds: average completion 95%**			
**Baseline**	N=27	N=28	N=55
Mean (SD)	20.8 (15.2)	21.3 (23.5)	21.1 (19.7)
Median (p25, p75)	17 (12, 24)	14.5 (11.5, 23)	17 (12, 23)
Unable to complete	0	0	0
**Final**	N=23	N=22	N=45
Mean (SD)	21.0 (19.1)	17.8 (8.4)	19.5 (14.8)
Median (p25, p75)	16 (11, 13)	14.5 (13, 22)	15 (11, 23)
Unable to complete	0	2	0
**TUDS, seconds: average completion 93%**			
**Baseline**	N=17	N=27	N=44
Mean (SD)	61.4 (33.0)	66.9 (38.5)	64.7 (36.2)
Median (p25, p75)	50 (39, 72)	51 (41, 88)	50.5 (41, 88)
Unable to complete	9	1	10
**Final**	N=19	N=21	N=40
Mean (SD)	59.2 (29.2)	68.1 (56.7)	63.9 (45.4)
Median (p25, p75)	51 (33, 79)	48 (39, 74)	48.5 (38, 76.5)
Unable to complete:	4	3	7
**2mWT, metres: average completion 95%**			
**Baseline**	N=27	N=28	N=55
Mean (SD)	81.0 (31.4)	94.7 (33.5)	87.9 (33.5)
Median (p25, p75)	77 (60, 110)	95 (68, 113)	83 (61, 113)
**Final**	N=23	N=24	N=47
Mean (SD)	88.8 (40.0)	83.9 (41.5)	86.3 (39.5)
Median (p25, p75)	80 (62, 120)	80 (57.5, 106)	80 (60, 108)
**BBS, scored 0-56: average completion 92%**			
**Baseline**	N=26	N=27	N=53
Mean (SD)	39.5 (11.3)	42.9 (8.1)	41.3 (9.8)
Median (p25, p75)	41.5 (32, 48)	43 (36, 51)	43 (34, 48)
**Final**	N=22	N=24	N=46
Mean (SD)	40.7 (12.9)	39.4 (11.6)	40.0 (12.1)
Median (p25, p75)	44 (30, 51)	41.5 (33, 49)	41.5 (33, 50)
**Activity monitor: minutes stepping per day**			
**Baseline**	N=26	N=27	N=53
Mean (SD)	29.9 (30.8)	31.1 (24.9)	30.5 (27.7)
Median (p25, p75)	24.7 (9.4, 31.4)	26 (14.5, 40.3)	25.9 (10.6, 34.1)
**Final**	N=22	N=25	N=47
Mean (SD)	27.7 (24.0)	31.2 (25.8)	29.6 (24.8)
Median (p25, p75)	24.1 (3.6, 45.0)	25.9 (17.3, 31.1)	25.7 (10.6, 40.3)
**Activity monitor: steps taken per day**			
**Baseline**	N=26	N=27	N=53
Mean (SD)	1755.5 (1963.7)	1750.9 (1369.6)	1753.2 (1670.9)
Median (p25, p75)	1292.3 (587.1, 1835.7)	1572.9 (802.3, 2234.6)	1448.6 (654.6, 2144.0)
**Final**	N=22	N=25	N=47

For peer review only - http://bmjopen.bmj.com/site/about/guidelines.xhtml

Mean (SD)	1673.2 (1594.4)	1836.1 (1647.8)	1759.9 (1607.4)
Median (p25, p75)	1356.4 (204.0, 2719.4)	1510.3 (922.9, 1908.0)	1446.3 (551.4, 2270.0)

TUG (Timed Up and Go test) and TUDS (Timed Up and Down Stair test): faster time indicates better performance; 2mWT (2-minute walk test): further distance indicates better walking ability; BBS (Berg Balance Scale): higher score indicates better balance.

For peer review only

For peer review only - http://bmjopen.bmj.com/site/about/guidelines.xhtml

Table 4: EQ-5D-5L mean (SD) utility scores, unadjusted mean difference (95% CI) and adjusted for baseline utility at each time point (baseline, interim and final follow-up), by trial arm

	**Self-aligning (n=27)**	**Standard (n=28)**	**Unadjusted difference (self-aligning vs. standard) (95% CI)**	**Adjusted difference (self-aligning vs. standard) (95% CI)**
**EQ-5D-5L (average completion 93%)**				
**Baseline**	0.62 (0.30)	0.63 (0.35)	-0.009 (-0.188, 0.169)	-0.009 (-0.188, 0.169)
**Interim**	0.64 (0.18)	0.57 (0.24)	0.067 (-0.055, 0.189)	0.104 (0.001, 0.207)
**Final**	0.75 (0.16)	0.63 (0.32)	0.120 (-0.021, 0.262)	0.117 (0.017, 0.216)

For peer review only

Twitter
Follow Natalie Vanicek @NatalieVanicek and Cleveland Barnett @Cleve_Barnett

Acknowledgements
This work uses data provided by patients and collected by researchers and members of the NHS who worked collaboratively on the STEPFORWARD trial. The collection of data would not have been possible without the support of the participating NHS Trusts and their staff. The authors would also like to thank the members of the Project Advisory Group (PAG): Dennis Harrison, Peter Wignall, Anthony Gick and Kevin Waller for their invaluable advice on many aspects of the project.

Author contributions
NV led on the conception, design and writing of this manuscript. NM, EC, JW, KB, CMcD, CB, MT, FJ, AS and DH contributed to the design, writing, critical review of intellectual content and final manuscript approval. All authors agree to be accountable for their work. As Chief Investigator, NV takes overall responsibility for the work. EC provided statistical expertise in the study design and development stages of the project and the protocol. NM, JW, and CMcD made substantial contributions to the trial design and management. DH provided particular input to PPI. KB was specifically responsible for the health economic aspects of the study design and CB, MT, FJ and AS were responsible for providing expert clinical support.

Funding statement
This paper presents independent research funded by the National Institute for Health Research (NIHR) under its Research for Patient Benefit (RfPB) Programme (Grant Reference Number PB-PG-0816-20029). Hull University Teaching Hospitals NHS Trust are the sponsors of the study, on behalf of the funder.

Blatchford (Basingstoke, UK) provided the prosthetic feet examined in this research at no cost. The prosthetic feet are currently commercially available devices. Blatchford have had no part in the study design nor are they be involved directly in the research, its subsequent analysis and ultimate dissemination.

Disclaimer
The views expressed are those of the author(s) and not necessarily those of the NHS, the NIHR, the Department of Health and Social Care, or Blatchford.

Patient and public involvement
Patients and public involvement members were involved in the design, conduct, reporting and dissemination plans of this research.

Patient consent for publication
Not required

Competing interests
None declared.

For peer review only

Ethics approval
Ethical approval by Yorkshire & The Humber – Leeds West Research Ethics Committee (IRAS project ID 232950; REC reference 18/YH/0089) and HRA approval were obtained on 4th May 2018.

Data availability statement
The data that support the findings of this study are available from the corresponding author, [NV], upon reasonable request.

For peer review only

For peer review only - http://bmjopen.bmj.com/site/about/guidelines.xhtml

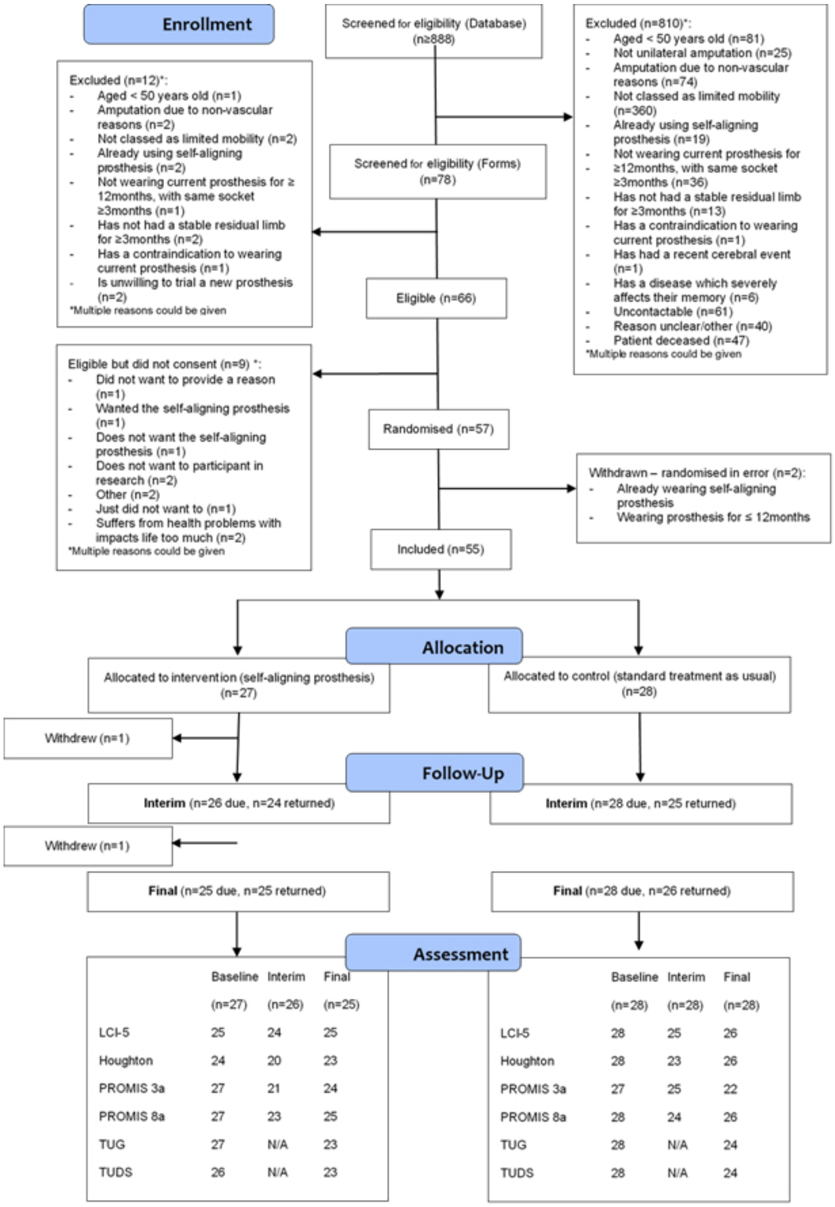

Figure 1: CONSORT flowchart for the STEPFORWARD study

161x233mm (96 x 96 DPI)

CONSORT 2010 checklist of information to include when reporting a pilot or feasibility trial*

Section/Topic	Item No	Checklist item	Reported on page No
**Title and abstract**			
	1a	Identification as a pilot or feasibility randomised trial in the title	1
	1b	Structured summary of pilot trial design, methods, results, and conclusions (for specific guidance see CONSORT abstract extension for pilot trials)	2
**Introduction**			
Background and objectives	2a	Scientific background and explanation of rationale for future definitive trial, and reasons for randomised pilot trial	4-5
	2b	Specific objectives or research questions for pilot trial	5
**Methods**			
Trial design	3a	Description of pilot trial design (such as parallel, factorial) including allocation ratio	5
	3b	Important changes to methods after pilot trial commencement (such as eligibility criteria), with reasons	n/a
Participants	4a	Eligibility criteria for participants	5-6
	4b	Settings and locations where the data were collected	6
	4c	How participants were identified and consented	6
Interventions	5	The interventions for each group with sufficient details to allow replication, including how and when they were actually administered	6
Outcomes	6a	Completely defined prespecified assessments or measurements to address each pilot trial objective specified in 2b, including how and when they were assessed	7
	6b	Any changes to pilot trial assessments or measurements after the pilot trial commenced, with reasons	n/a
	6c	If applicable, prespecified criteria used to judge whether, or how, to proceed with future definitive trial	n/a
Sample size	7a	Rationale for numbers in the pilot trial	6
	7b	When applicable, explanation of any interim analyses and stopping guidelines	n/a
Randomisation:			
Sequence generation	8a	Method used to generate the random allocation sequence	n/a
	8b	Type of randomisation(s); details of any restriction (such as blocking and block size)	6
Allocation concealment mechanism	9	Mechanism used to implement the random allocation sequence (such as sequentially numbered containers), describing any steps taken to conceal the sequence until interventions were assigned	n/a

For peer review only

Implementation	10	Who generated the random allocation sequence, who enrolled participants, and who assigned participants to interventions	6
Blinding	11a	If done, who was blinded after assignment to interventions (for example, participants, care providers, those assessing outcomes) and how	n/a
	11b	If relevant, description of the similarity of interventions	n/a
Statistical methods	12	Methods used to address each pilot trial objective whether qualitative or quantitative	7
**Results**			
Participant flow (a diagram is strongly recommended)	13a	For each group, the numbers of participants who were approached and/or assessed for eligibility, randomly assigned, received intended treatment, and were assessed for each objective	8, Figure 1
	13b	For each group, losses and exclusions after randomisation, together with reasons	8, Figure 1
Recruitment	14a	Dates defining the periods of recruitment and follow-up	6
	14b	Why the pilot trial ended or was stopped	n/a
Baseline data	15	A table showing baseline demographic and clinical characteristics for each group	Table 1
Numbers analysed	16	For each objective, number of participants (denominator) included in each analysis. If relevant, these numbers should be by randomised group	Tables 1-4
Outcomes and estimation	17	For each objective, results including expressions of uncertainty (such as 95% confidence interval) for any estimates. If relevant, these results should be by randomised group	Tables 1-4
Ancillary analyses	18	Results of any other analyses performed that could be used to inform the future definitive trial	n/a
Harms	19	All important harms or unintended effects in each group (for specific guidance see CONSORT for harms)	8
	19a	If relevant, other important unintended consequences	n/a
**Discussion**			
Limitations	20	Pilot trial limitations, addressing sources of potential bias and remaining uncertainty about feasibility	13
Generalisability	21	Generalisability (applicability) of pilot trial methods and findings to future definitive trial and other studies	10-13
Interpretation	22	Interpretation consistent with pilot trial objectives and findings, balancing potential benefits and harms, and considering other relevant evidence	10-13
	22a	Implications for progression from pilot to future definitive trial, including any proposed amendments	14
**Other information**			
Registration	23	Registration number for pilot trial and name of trial registry	2
Protocol	24	Where the pilot trial protocol can be accessed, if available	5-6
Funding	25	Sources of funding and other support (such as supply of drugs), role of funders	24
	26	Ethical approval or approval by research review committee, confirmed with reference number	25

For peer review only

Citation: Eldridge SM, Chan CL, Campbell MJ, Bond CM, Hopewell S, Thabane L, et al. CONSORT 2010 statement: extension to randomised pilot and feasibility trials. BMJ. 2016;355.
*We strongly recommend reading this statement in conjunction with the CONSORT 2010, extension to randomised pilot and feasibility trials, Explanation and Elaboration for important clarifications on all the items. If relevant, we also recommend reading CONSORT extensions for cluster randomised trials, non-inferiority and equivalence trials, non-pharmacological treatments, herbal interventions, and pragmatic trials. Additional extensions are forthcoming: for those and for up to date references relevant to this checklist, see www.consort-statement.org.

For peer review only

The STEPFORWARD study: a randomised controlled feasibility trial of a self-aligning prosthetic ankle-foot for older patients with vascular-related amputations

Journal:	*BMJ Open*
Manuscript ID	bmjopen-2020-045195.R3
Article Type:	Original research
Date Submitted by the Author:	18-Feb-2021
Complete List of Authors:	Vanicek, Natalie; University of Hull, Sport, Health and Exercise Science<br>Coleman, Elizabeth; University of York, Health Sciences<br>Watson, Judith; University of York, Health Sciences<br>Bell, Kerry; University of York, Health Sciences<br>McDaid, Catriona; University of York, Health Sciences<br>Barnett, Cleveland; Nottingham Trent University, School of Science and Tecnology<br>Twiste, Martin; University of Salford<br>Jepson, Fergus; Lancashire Teaching Hospitals NHS Foundation Trust, Specialist Mobility Rehabilitation Centre<br>Salawu, Abayomi; Hull University Teaching Hospitals NHS Trust, Disability Medicine and Rehabilitation Unit<br>Harrison, Dennis<br>Mitchell, Natasha; University of York, York Trials Unit, Department of Health Sciences
**Primary Subject Heading**:	Rehabilitation medicine
Secondary Subject Heading:	Patient-centred medicine
Keywords:	REHABILITATION MEDICINE, Clinical trials < THERAPEUTICS, Foot & ankle < ORTHOPAEDIC & TRAUMA SURGERY, Musculoskeletal disorders < ORTHOPAEDIC & TRAUMA SURGERY

I, the Submitting Author has the right to grant and does grant on behalf of all authors of the Work (as defined in the below author licence), an exclusive licence and/or a non-exclusive licence for contributions from authors who are: i) UK Crown employees; ii) where BMJ has agreed a CC-BY licence shall apply, and/or iii) in accordance with the terms applicable for US Federal Government officers or employees acting as part of their official duties; on a worldwide, perpetual, irrevocable, royalty-free basis to BMJ Publishing Group Ltd ("BMJ") its licensees and where the relevant Journal is co-owned by BMJ to the co-owners of the Journal, to publish the Work in this journal and any other BMJ products and to exploit all rights, as set out in our licence.

The Submitting Author accepts and understands that any supply made under these terms is made by BMJ to the Submitting Author unless you are acting as an employee on behalf of your employer or a postgraduate student of an affiliated institution which is paying any applicable article publishing charge ("APC") for Open Access articles. Where the Submitting Author wishes to make the Work available on an Open Access basis (and intends to pay the relevant APC), the terms of reuse of such Open Access shall be governed by a Creative Commons licence – details of these licences and which Creative Commons licence will apply to this Work are set out in our licence referred to above.

Other than as permitted in any relevant BMJ Author's Self Archiving Policies, I confirm this Work has not been accepted for publication elsewhere, is not being considered for publication elsewhere and does not duplicate material already published. I confirm all authors consent to publication of this Work and authorise the granting of this licence.

For peer review only

Title page:
The STEPFORWARD study: a randomised controlled feasibility trial of a self-aligning prosthetic ankle-foot for older patients with vascular-related amputations

Authors: Natalie Vanicek[1], Elizabeth Coleman[2], Judith Watson[2], Kerry Bell[2], Catriona McDaid[2], Cleveland Barnett[3], Martin Twiste[4], Fergus Jepson[5], Abayomi Salawu[6], Dennis Harrison[7] and Natasha Mitchell[2]

Corresponding author: Professor Natalie Vanicek
Department of Sport, Health and Exercise Science
Faculty of Health Sciences
University of Hull
Hull
HU6 7RX
E: n.vanicek@hull.ac.uk
T: 01482 463607

Author affiliations
[1] Department of Sport, Health and Exercise Science, University of Hull, Hull, UK
n.vanicek@hull.ac.uk Orcid iD 000-0002-9602-3172
[2] York Trials Unit, Department of Health Sciences, University of York, York, UK
izzy.coleman@york.ac.uk Orcid iD 0000-0003-4210-1865
jude.watson@york.ac.uk Orcid iD 0000-0003-0694-3854
kerry.bell@york.ac.uk Orcid iD 0000-0001-5124-138X
catriona.mcdaid@york.ac.uk Orcid iD 0000-0002-3751-7260
natasha.mitchell@york.ac.uk Orcid iD 0000-0002-6904-2976
[3] School of Science and Technology, Nottingham Trent University, Nottingham, UK
cleveland.barnett@ntu.ac.uk Orcid iD 0000-0001-6898-9095
[4] School of Health and Society, University of Salford, Manchester, UK
m.twiste@salford.ac.uk Orcid iD 0000-0003-2724-3283
[5] Specialist Mobility Rehabilitation Centre, Lancashire Teaching Hospitals NHS Foundation Trust, Preston, UK
Fergus.Jepson@lthtr.nhs.uk
[6] Disability Medicine and Rehabilitation Unit, Hull University Teaching Hospitals NHS Trust, Hull, UK
abayomi.salawu@hey.nhs.uk Orcid iD 0000-0003-2496-0826
[7] Public Involvement Member
dharrisonpublic@gmail.com

Keywords
Randomised controlled trial, feasibility, self-aligning, ankle-foot prosthesis, transtibial amputation, acceptability, vascular

Word count: 4,971 words

For peer review only - http://bmjopen.bmj.com/site/about/guidelines.xhtml

ABSTRACT

Objectives: To determine the feasibility of conducting a full-scale randomised controlled trial (RCT) of the effectiveness and cost-effectiveness of a self-aligning prosthetic ankle-foot compared with a standard prosthetic ankle-foot.

Design: Multi-centre parallel group feasibility RCT.

Setting: Five prosthetics centres in England recruiting from July 2018 to August 2019.

Participants: Adults aged ≥ 50 years with a vascular-related or non-traumatic transtibial amputation for one year or longer, categorised as having 'limited community mobility' and using a non-self-aligning ankle-foot.

Intervention: Participants were randomised into one of two groups for 12 weeks: self-aligning prosthetic ankle-foot or existing non-self-aligning prosthetic ankle-foot.

Outcomes: Feasibility measures: recruitment, consent and retention rates; and completeness of questionnaire and clinical assessment datasets across multiple time points. Feasibility of collecting daily activity data with wearable technology and health resource use data with a bespoke questionnaire.

Results: Fifty-five participants were randomised (61% of the target 90 participants): n=27 self-aligning ankle-foot group, n=28 non-self-aligning ankle-foot group. Fifty-one participants were included in the final analysis (71% of the target number of participants). The consent rate and retention at final follow-up were 86% and 93%, respectively. The average recruitment rate was 1.25 participants/site/month (95% CI 0.39 to 2.1). Completeness of questionnaires ranged from 89-94%, and clinical assessment were 92-95%, including the activity monitor data. The average completion rates for the EQ-5D-5L and bespoke resource use questionnaire were 93% and 63%, respectively.

Conclusions: This feasibility trial recruited and retained participants who were categorised as having 'limited community mobility' following a transtibial amputation. The high retention rate of 93% indicated the trial was acceptable to participants and feasible to deliver as a full-scale RCT. The findings support a future, fully-powered evaluation of the effectiveness and cost-effectiveness of a self-aligning prosthetic ankle-foot compared to a standard non-self-aligning version with some adjustments to the trial design and delivery.

Trial registration

ISRCTN15043643

For peer review only - http://bmjopen.bmj.com/site/about/guidelines.xhtml

ARTICLE SUMMARY

Strengths and limitations of this study

- This was one of few clinical trials investigating ankle-foot prostheses within prosthetics services in the UK. The study design is a multi-centre, randomised controlled trial in an under-researched clinical population involving prosthetics.
- The completeness of subjective and objective assessment measures, administered in a clinical setting and via post, was high.
- Feasibility and acceptability were ascertained with quantitative and trial procedure data.
- Recruitment was lower and slower than anticipated, but the ability to retain participants was high.
- Effectiveness and cost-effectiveness of a self-aligning ankle-foot will need to be established in a definitive full-scale trial.

For peer review only

For peer review only - http://bmjopen.bmj.com/site/about/guidelines.xhtml

INTRODUCTION

In the UK, there are approximately 6,000 new referrals to prosthetics services every year. Most of these referrals are patients aged over 50 years who have had an amputation at the transtibial (below-knee) level.[1] The majority of lower limb amputations result from long-term diabetes mellitus, coronary and peripheral vascular diseases.[2, 3] Consequently, most new referrals for a prosthesis involve older adults who have other health comorbidities and who are categorised as having 'limited community mobility'. These patients are an under-researched group, with few studies focused on their mobility with a prosthesis.

Many factors determine the type of prosthesis that a patient receives, such as estimation of patient outcomes and goals, and the healthcare budget.[4] A Cochrane Review reported there was insufficient evidence from robust studies to support the "overall superiority of any individual type of prosthetic ankle-foot mechanism".[5] There are also no standardised criteria for the prescription of ankle-foot prostheses. Practice varies across UK centres and is frequently cost-driven. Subsequently, the majority of older people with a transtibial amputation receive a standard, non-self-aligning ankle-foot, such as the non-articulated solid ankle cushioned heel (SACH), uniaxial or multiaxial prosthetic foot. These prosthetic ankle-feet have limited functionality. The SACH foot has a solid 'ankle' that does not allow any movement. The uniaxial ankle-foot is only capable of moving about a single axis with some ankle dorsi- and plantar-flexion; the multiaxial ankle-foot can rotate about multiple axes, allowing ankle dorsi- and plantar-flexion and eversion-inversion. The elastic recoil to return to a plantigrade position makes the uni- and multi-axial ankle-feet unable to self-align to inclined surfaces, making these feet more suitable for standing or walking on level ground. This requires the user to make compensatory actions at proximal joints (compensations at the hip bilaterally and intact knee of the affected limb) and at the intact ankle to achieve stability with a 'foot flat' position when walking on slopes.[6, 7] These actions result in greater asymmetry between the two limbs and increase the metabolic requirements of walking.[6] This can make walking more difficult and tiring. Our public involvement members reported that a poorly functioning prosthesis can contribute to sedentary behaviour, pain and more frequent visits to prosthetics centres and other healthcare services, which may lead to disuse and poorer quality-of-life. Therefore, identifying a more functionally suitable prosthesis could benefit patients in meaningful ways following amputation.

A 'family' of prosthetic ankle-feet that use a hydraulic mechanism to self-align to sloped surfaces have been designed to attenuate many of the burdens associated with standard, non-self-aligning ankle-feet. These prosthetic ankle-feet have been designed for users of varying ability (more active users, K3-K4 classification) and also for users categorised as having 'limited community mobility' (K2 users).[8] The hydraulic mechanism allows the prosthetic ankle-foot to self-align to sloped surfaces (due to ground reaction forces) and remain in a dorsiflexed position throughout the swing phase. This can improve ground

For peer review only - http://bmjopen.bmj.com/site/about/guidelines.xhtml

clearance and securing the biological knee, which is important for falls prevention.[9] Previous laboratory-based studies reported that a self-aligning ankle-foot reduced residuum-socket interface pressures in active users,[10] which could alleviate pain in the residuum longer-term, improved walking[11] and enhanced quality of life[12, 13] in users with lower activity levels. More functional prostheses may improve patient function and mobility following a lower limb amputation.[14] However, the effects of a self-aligning prosthesis have not been evaluated with robust randomised controlled trials (RCTs) in a community setting. In particular there is a lack of research involving participants who have had a non-traumatic (e.g., vascular-related) amputation. A self-aligning ankle-foot is more expensive than a non-self-aligning version by approximately £900 for the K2-user version (at 2020 UK prices) with variations in price across manufacturers. However, the potential patient benefits, such as improved mobility, better quality-of-life, and reduced falls, could offset future healthcare and socioeconomic costs.

There is a need for a RCT of the effectiveness and cost-effectiveness of a self-aligning prosthetic ankle-foot for older patients, with a vascular-related or non-traumatic transtibial amputation, compared to a standard, non-self-aligning ankle-foot. The STEPFORWARD study used a mixed-methods approach to determine feasibility of conducting such a study. A feasibility study was considered important in advance of a full-scale RCT given the lack of large community-based RCTs in this field. A full-scale trial will be powered appropriately to detect a difference in the primary outcome between the two trial arms (and as such has a sample size large enough to answer a research question on intervention effectiveness). A feasibility study addresses the question of whether a specific study can be done, whether it should proceed and, if so, how.[15] It is not designed to address the question of whether an intervention works. This article reports the feasibility as determined by the quantitative data, which was assessed according to: (1) participant recruitment, including time to recruit, consent and retention rates and adverse events; (2) completeness of data across multiple time points, including health economics data while piloting a bespoke health resource use questionnaire; and (3) day-to-day activity measured with wearable technology. The findings from this work could be relevant to clinical practitioners, researchers and policy makers.

METHODS

Study design and participants

The STEPFORWARD trial was a multi-centre, parallel group, randomised controlled feasibility trial to assess the possibility of conducting a full-scale RCT in the future, which would be adequately powered to answer the primary research question. The trial received favourable ethical approval from the National Health Service Yorkshire and the Humber—Leeds West Research Ethics Committee (ref:18/YH/0089) and Health Research Authority in May 2018. The trial was registered on the ISRCTN clinical trial registry (ISRCTN15043643). The full study protocol, with detailed inclusion/exclusion criteria and recruitment/screening pathways, has been published[16] and is briefly described below.

For peer review only - http://bmjopen.bmj.com/site/about/guidelines.xhtml

We aimed to recruit 90 participants who were aged ≥ 50 years with a unilateral transtibial amputation for ≥1 year due to vascular, neurological or life-limiting reasons, who were categorised as having 'limited community mobility', with a stable residuum, and using a non-self-aligning prosthetic ankle-foot (e.g., SACH, uniaxial, multiaxial (e.g., Multiflex) or other K1/K2 feet). We excluded anyone who had a contraindication to wearing their current prosthesis or the self-aligning ankle-foot (according to the manufacturer's specifications), had experienced a recent cerebrovascular event or had a disease affecting their memory. Allowing for 20% attrition, we anticipated 72 participants in our final analysis.

Recruitment and randomisation

We used a two-stage screening process to identify eligible patients; full details are provided in the published protocol.[16] In summary, potential participants were identified by database screening or during routine appointments to five prosthetics centres in England from July 2018 to August 2019. They were posted a study invitation pack and asked to return a Consent to Contact form if they were interested in being contacted about the trial. A member of their multidisciplinary team then contacted them and completed the first section of the Screening Form over the telephone. If potentially eligible, they were invited into clinic to complete the outstanding screening questions, which had to be done face-to-face, and provide their written consent. Following consent, eligible patients completed all baseline measures before being randomised into one of two trial arms: keep their non-self-aligning prosthetic ankle-foot (standard treatment group) or receive a new self-aligning ankle-foot (intervention group). Participants were randomised individually, stratified according to prosthetics centre on a 1:1 basis, by a telephone randomisation service set up by York Trials Unit. Blinding of the participants and investigators was not possible because of the nature of the intervention.

Standard treatment and intervention

Participants in the standard treatment group continued using their normal prosthetic ankle-foot and had access to all clinical services as normal. They were asked to carry on with their normal daily routine during the intervention period.

Participants in the intervention group were fitted with the self-aligning prosthetic ankle-foot by their regular prosthetist. The self-aligning ankle-foot used in this trial was the Avalon^{K2}, manufactured by Blatchford, Basingstoke, UK (Patent reg: 5336386), which was already commercially available and could be prescribed under the national health service (NHS). Once fitted, participants were asked to ambulate with it as they would normally with any new prosthetic ankle-foot for approximately 12 weeks after fitting (intervention period). At the end of the study, participants in the intervention group only could keep the self-aligning ankle-foot if they preferred it. Participants in standard treatment group were not offered

For peer review only - http://bmjopen.bmj.com/site/about/guidelines.xhtml

the self-aligning ankle-foot at the end of the study as it is not known whether it is better or more acceptable to users than the standard prosthesis.

Outcome measures

The primary outcome of this feasibility study related to collecting data on recruitment, consent and retention rates. Reasons for non-participation or withdrawal were recorded where possible. We assessed the completeness of data across multiple time points: baseline, interim and final follow-ups. The baseline and final assessments were conducted at the participant's usual prosthetics centre. At the interim follow-up, only questionnaire data were collected via post. Data were gathered from questionnaires (baseline demographics and bespoke health resource use questionnaires, the Locomotor Capabilities Index (LCI-5),[17] Houghton Scale,[18] the Patient-Reported Outcomes Measurement Information System (PROMIS) Short-Form v1.0 Pain (3a and 8a) questionnaires[19] and EQ-5D-5L[20]) and from clinical assessments (Timed Up and Go (TUG) test,[21] Timed Up and Down Stairs (TUDS) test,[22] 2-minute walk test (2mWT)[23] and Berg Balance Scale (BBS)[24]). Data related to daily step count and time spent walking were collected from a loaned activity monitor (activPAL4™, PAL Technologies Ltd, Glasgow, UK) worn on the prosthesis for one week. At baseline, all participants wore their normal (non-self-aligning) prosthetic ankle-foot during the clinical assessments and activity monitor data collection; at final follow-up they wore whichever prosthetic ankle-foot they were using currently. Adverse events (AEs) related to the prosthesis only, and any serious adverse events (SAEs), were recorded throughout the study.

Statistical analyses

The recruitment rate was reported monthly, and overall, by site. An average monthly recruitment rate was calculated, with a 95% confidence interval (CI) estimated from the data collected. The number of eligible patients and those approached for consent was summarised overall, by site, using counts and percentages.

Baseline data were summarised by trial arm, as randomised, with no formal comparison between the groups. Continuous data were reported descriptively and categorical data by counts and percentages. Completion rates of all the clinical outcome measures were reported by trial arm and overall.

Health economic analysis

The health economics work investigated the feasibility of collecting self-reported patient use of health service resources (e.g., primary care visits, prescription medication) over the study period and to evaluate the appropriateness of these data. This information would help to refine the bespoke questionnaire in a future trial. A full cost-effectiveness analysis was not completed as this was a feasibility study. Nonetheless, the costing approach was undertaken from an NHS perspective; unit costs were derived from established national costing sources

For peer review only - http://bmjopen.bmj.com/site/about/guidelines.xhtml

such as NHS Reference Costs[25] and Personal Social Services Research Unit costs of health and social care.[26]

RESULTS

Feasibility assessment

Recruitment

Over 880 potential participants were initially screened by searching patient databases across the five prosthetics centres. Seventy-eight were identified as potentially eligible after the first screening, but 12 were subsequently excluded following the final, second screening. Eighty-six percent of those screened, who were found to be fully eligible, consented to participating (55 of 64 participants) in the trial. The denominator was 64 as two were randomised in error and subsequently withdrawn (Figure 1). As we used a two-stage screening process, only potential participants who met the initial inclusion criteria were approached; those uninterested in being involved during the initial screening were not screened further. Therefore, the consent rate may be artificially high. Reasons for non-participation are also presented in Figure 1. In total, 55 participants (61% of the target 90 participants) were included and randomised as follows: 27 to the self-aligning prosthetic ankle-foot (intervention) group and 28 to the non-self-aligning (standard treatment) group.

Initial recruitment was slower than anticipated, therefore two additional recruiting sites were included midway through the trial, bringing the total number of participating sites to five. The average recruitment rate was 1.25 participants/site/month (95% CI 0.39 to 2.1) ranging from 0.83 to 2.2 participants per month. Each site recruited approximately the same number of participants (mean=11, range=9-13 participants), despite them being open to recruitment for different lengths of time (range=5.0-13.7 months).

Participants

The mean (SD) age of participants in this study was 68.8 (9.6) years, ranging from 51.8 to 86.8 years. Most participants were male (85.5%) and all classified themselves as White British, Irish or other. The most common reason for amputation was diabetes (45.5%). Full participant characteristics are described in Table 1.

Follow-up and adverse events

In addition to the two people randomised in error and withdrawn, a further two participants who were allocated to the intervention group withdrew from the study: one as a result of having suffered a stroke and one reported the self-aligning prosthetic ankle-foot was too heavy. At final follow-up, 51 of the 55 participants returned the questionnaires giving a retention rate of 93%. This was 71% of the planned target of 72 participants included in the final analysis.

For peer review only - http://bmjopen.bmj.com/site/about/guidelines.xhtml

A total of four SAEs and one AE were reported. The SAEs were due to the following reasons: stroke; fall and femoral fracture when not wearing their prosthesis (both of these cases occurred in the intervention group); fall and hip fracture, leading to hospitalisation and subsequently death; and recurrent falls with fibular fracture (both of these cases were in the standard treatment group). The AE occurred because one participant (intervention group) developed blistering on their residuum.

Data completeness

There were four standardised measures completed at all three time points: LCI-5, Houghton, PROMIS 3A and PROMIS 8A. The average completion rate across all time points ranged from 89-94%. Data from four clinical assessments (TUG, TUDS, 2mWT and BBS), and an activity monitor worn for a week, were collected at both baseline and final follow-up, and their average completion rate ranged from 92-95%. All the measures were well completed, or attempted, with at least 75% completed in full. Details of the outcome measures and completion rates can be found in Tables 2 and 3.

None of the four standardised measures appeared to show a change between baseline and follow-up. For the 2-minute walk test, participants with the self-aligning prosthetic ankle-foot walked on average further at final follow-up (mean (SD): 88.8 (40.0) metres) than at baseline (81.0 (31.4) metres). This equates to an average increase of 6.2 (16.2) metres in walking distance for the participants in the intervention group between these two time points. However, in the standard treatment group, there was an average decrease of 9.0 (29.8) metres, indicating that these participants were walking less distance over two minutes at the final follow-up. Scores for the TUG, TUDS and BBS remained largely unchanged across both groups. Activity monitor data revealed that the daily time spent stepping between baseline and follow-up was mostly unchanged for both groups, which was approximately 30 minutes per day. Conversely, the number of steps taken per day decreased in the intervention group (1755.5 (1963.7) steps at baseline vs. 1673.2 (1594.4) steps at final follow-up) yet increased in the standard treatment group (1750.9 (1369.9) steps at baseline vs. 1836.1 (1647.8) steps at final follow-up). The average change in number of steps taken per day decreased by 2.9 (756.2) steps in the intervention group between baseline and final follow-up, yet increased by 283.3 (1614.8) steps in the standard treatment group. In both groups, the variability in daily stepping was high.

Health economic data

The health economic outcomes are based on the patient-completed questionnaires, which incorporated the EQ-5D-5L and a bespoke resource use questionnaire. The average completion rates were 93% and 63%, respectively. Over 89% of participants completed the EQ-5D-5L fully. At baseline, responses to the EQ-5D-5L indicated relatively equal levels of health utility between the trial arms. By final follow-up, an increase in mean utility of 0.13 in the intervention group was observed, whilst mean utility in the standard treatment group

For peer review only - http://bmjopen.bmj.com/site/about/guidelines.xhtml

remained the same. A positive mean difference in favour of the self-aligning prosthetic ankle-foot in the intervention group remained consistent after adjusting for baseline utility (Table 4).

For the resource use questionnaire, the completion rates did not differ by the respective components, suggesting all sections were acceptable and each type of data was feasible to collect. Resource use was minimal across both primary and secondary/tertiary care for participants in both arms of the trial. The most frequently received care was via outpatient attendances at NHS hospitals (mean (SD): 0.21 (0.51) rising to 2.04 (3.72) visits from baseline to final follow-up for the intervention group; 0.08 (0.27) rising to 1.14 (2.19) visits for the standard treatment group.

Medications were grouped according to comorbidity to investigate their inclusion in a full-scale trial as follows: blood pressure/heart problems, neuropathic pain, diabetes, phantom pain. Participant uptake of the groups of medications explored using the resource use questionnaire was high, and there were some differences between groups, suggesting a micro-costing exercise including medications data may be merited in a full-scale trial. Approximately 73% of participants overall reported taking medications related to blood pressure and/or heart problems, and 62% of participants overall took medications for diabetes. Medication use appeared to increase over the course of the trial for participants in both arms.

The intervention (self-aligning prosthetic ankle-foot) was delivered in the NHS setting by the usual staff providing post-amputation aftercare (prosthetist, physiotherapist). Other than the self-aligning ankle-foot itself, participants received equal care to standard treatment. The cost (at 2020 UK prices) of the self-aligning ankle-foot used in the trial was £1192 compared to the non-self-aligning version estimated at £287 (although this depends on the exact standard ankle-foot and would need to be determined accurately based on the user's actual foot). The additional intervention cost to the NHS of delivering the self-aligning ankle-foot is therefore approximately £905.

Identification of primary outcome measure

The findings from this study have demonstrated feasibility, acceptability and a signal of efficacy for three measures: walking ability with the 2mWT, daily activity with activity monitor data, and quality-of-life with the EQ-5D-5L. We consulted with our patient advisory group (PAG) to inform the appropriateness of a primary outcome to be used in a future full-scale trial. The PAG members reported that, of the three measures, the EQ-5D-5L resonated most strongly with them as it captured the constructs that were important to them (e.g., mobility, pain, usual activities, general health). This measure will be considered for use as the primary outcome in a future trial.

For peer review only - http://bmjopen.bmj.com/site/about/guidelines.xhtml

DISCUSSION

Main findings

The primary objective of this study was to determine the feasibility of conducting a future, full-scale RCT of the effectiveness and cost-effectiveness of a self-aligning prosthetic ankle-foot compared to a standard prosthetic ankle-foot. There are few clinical trials involving older adults living with a lower limb amputation, who are an under-researched but growing group of patients. The consent, retention and completion rates were high, demonstrating that it is feasible to recruit and retain participants to a future trial addressing the research question. While the consent rate may be artificially high, due to screening methods used in the current study, the final retention and completion rates indicate the study was acceptable to participants.

We did not meet the recruitment target of 90. Given that only 7.4% of screened patients met the eligibility criteria (though only 7.2% were truly eligible, due to the randomisation in error), it is important to consider whether the criteria were too restrictive and should be adjusted in a future trial. It is common that the residuum undergoes considerable volume changes within the first year post-amputation, necessitating the fabrication of new prosthetic sockets. As the intervention period of this feasibility study was only 12 weeks long (due to resource constraints), we sought to recruit established prosthesis users who had no current contraindications to wearing their ankle-foot prosthesis, had a stable residuum volume and the same prosthetic socket for ≥ 3 months and were free of open wounds or infections on the residuum. We also only included patients who had a transtibial amputation secondary to a health condition, as this group is most likely categorised as having 'limited community mobility' and frequently prescribed a non-self-aligning prosthetic ankle-foot. It would be clinically relevant to broaden the eligibility criteria to include any patient with a transtibial amputation within this mobility category, regardless of cause and time since amputation, in a future study. This would widen the pool of patients potentially eligible for the trial. However, we do not have data from the feasibility study to assess impact on recruitment and it would be important for the future trial to have an internal pilot with clear stop-go criteria to confirm the feasibility of a modified study design.

Five AEs, of which four were categorised as serious, occurred throughout this trial. All of the SAEs (one stroke and three falls) were not deemed related to the self-aligning prosthetic ankle-foot. This clinical group is vulnerable to falling, with one recent study stating that over 57% of community-living lower limb prosthesis users fall at least once a year[27] and that 36-75% of fallers experience recurrent falls.[27, 28] The participant who developed blistering on the residuum (the one AE) had been allocated to the intervention group. It is not uncommon for blistering to occur if the skin on the residuum is subjected to increased friction as a result of wearing the prosthesis for longer. In most instances, blisters heal relatively quickly, and the prosthesis user can soon return to normal function with their prosthesis.

For peer review only - http://bmjopen.bmj.com/site/about/guidelines.xhtml

Results in context with other research

A few randomised trials of several different prosthetic ankle-feet (although not self-aligning), evaluating function and self-report measures, have been conducted previously.[29-31] Some of these studies lacked sample size calculations and presented small samples (range n=10-27 participants/trial). Moreover, they represented a heterogeneous group in terms of their mobility level classifications (participants were mostly categorised as 'unlimited community ambulators' (K3) or 'active adults' (K4)), cause of amputation and health comorbidities. The age of the participants in these studies was also younger than in the current feasibility trial (mean range 42.3-57 years). Therefore, the findings from existing studies are difficult to generalise to the majority of people with a lower limb amputation due to dysvascularity, in the UK and other developed nations. We are not aware of any randomised trials investigating prosthetic ankle-feet uniquely involving older adults classified as having 'limited community mobility' as a result of health-related issues.

Outcome measures

This study was not designed to assess the effectiveness of the self-aligning prosthetic ankle-foot, and no formal comparisons between randomised groups were undertaken as the study was not powered to report effects. Three measures indicated a signal of potential efficacy including the 2-minute walk test (walking ability), daily activity as measured with activity monitors, and quality-of-life as measured with the EQ-5D-5L. Conversely, other measures (including some population-specific measures and common clinical assessments) did not indicate any difference.

We demonstrated that the use of activity monitors, and their implementation using postal delivery, was feasible in this patient group. We believe this was supported by our study procedures, which included regular participant information letters alerting them to the next stages of the trial, and a detailed bespoke leaflet explaining how participants were requested to fit the activity monitor onto the prosthesis themselves. As the monitor was affixed to the prosthesis, there were few issues with discomfort or discontinued use. Trials using subjective outcomes, where the outcome measure is self-reported by a participant or assessment of an event requires exercise of judgement by an observer, may overestimate the treatment effect.[32] Therefore, it was important to establish the feasibility of using this more objective outcome in a future trial.

On average, both groups took fewer than 1800 daily steps over 30 minutes throughout the day. The data related to daily steps and stepping time reinforced the largely sedentary behaviour experienced by this clinical group. Our findings are broadly similar to a previous study that reported an average of 1450 (SD 1309) steps/day in a group of older individuals (age: 64 (SD 9) years) with a predominantly transtibial amputation (95% of total participants) as a result of vascular disease when measured over 10 days.[33] Further research

For peer review only - http://bmjopen.bmj.com/site/about/guidelines.xhtml

is warranted to ascertain whether a more functional prosthetic ankle-foot, compared to current standard care with a non-self-aligning ankle-foot, has the long-term potential to increase daily stepping, which could have important implications for patient benefit.

Health economics

It was feasible to collect the data required for a full-scale trial economic evaluation from this patient population. The high levels of completion suggest acceptability of instruments. The EQ-5D-5L is likely to be sensitive to changes in health states over time. Some changes to the bespoke health resource use questionnaire could be made in future. Use of privately funded healthcare services was negligible so this section could potentially be omitted in a full-scale trial. Participant uptake of the medications in the resource use questionnaire was high and there were some differences between groups suggesting a micro-costing exercise including medications data may be merited in a full-scale trial. Although no resource use of walking aids, adaptations and accessories had an uptake greater than 20%, participants were established prosthesis users who may have already accessed the required adaptations prior to entering the trial. Therefore, if a future trial had broader eligibility criteria to include unestablished prosthesis users, it may be important to retain this item.

The approximate NHS cost of the intervention is ~£900 above current standard care as it is assumed that the self-aligning prosthetic ankle-foot is delivered in the usual NHS setting and by the usual staff. There are various self-aligning prosthetic ankle-feet available commercially (e.g., using hydraulic mechanisms vs. micro-processor controlled and with actively powered propulsion), at varying costs and suitability (e.g., some prosthetic ankle-feet are heavier and more suitable for different patient needs). At the present time, we have only considered a single prosthetic ankle-foot, but one that is designed for our clinical population of interest. A full health economics evaluation, as part of full-scale trial, would be able to evaluate whether the initial higher cost of the ankle-foot could be offset by reduced use of other health resources (e.g., medications, prosthetic adjustments) and/or increased patient quality-of-life.

Strengths and weaknesses

Prosthetics services are an under-researched area within the NHS. As this was one of few clinical trials conducted in this area in the UK, we now have a better understanding of the challenges of recruiting in this setting, such as the limited research resources at clinical sites and variation in how sites organise their patient database systems. The recruiting sites in England provided a wide geographical spread (North East, North West, East of England, Midlands and South of England); however, the participants recruited across these sites were homogenous in their ethnicity (self-reported as White British, Irish or other). In future, we will develop strategies to maximise participation from patients across different ethnic groups and nations in the United Kingdom.

For peer review only - http://bmjopen.bmj.com/site/about/guidelines.xhtml

The majority of participants were recruited after a member of their clinical team screened the patient database for potentially eligible participants and posted them a study invitation pack. Therefore, the majority of the screening forms (79%) were initially completed over the phone before the face-to-face screening process confirmed their final eligibility. A limitation of this feasibility study was our lack of prior knowledge of the management of patient databases, which varied across the sites. Four sites used an electronic database for patient records while one used a paper-based system only; one of these sites used three different patient record systems. Not all patient records were up-to-date, resulting in two participants being randomised in error because they did not fulfil the inclusion criteria fully, and they were subsequently withdrawn. In a future trial, it will be essential to gather relevant information about the organisation of patient records at the local participating sites prior to opening that site to facilitate effective recruitment. This would also help sites recruit participants more consistently across the recruitment period. We were concerned at the outset about the risk of 'resentful demoralisation' amongst participants allocated to usual care and were mindful of this in developing the patient information for the study. The fact that adherence to the study processes and completion of outcome measures were similar across groups provides some reassurance in this regard. However, this would need continuing attention in the design of a full-scale trial.

For peer review only

Future research

Based on the findings from this feasibility study, we propose some adaptations to the study protocol for a future full-scale trial. They include:

- broadening the participant eligibility criteria to include new patients with a transtibial amputation, who would be categorised as having 'limited community mobility' by their multi-disciplinary team, and therefore likely to receive a non-self-aligning prosthetic ankle-foot;
- lengthening the intervention period of accommodating to the self-aligning ankle-foot, and including a longer follow-up period, would be important for unestablished prosthesis users if the criteria were more inclusive in a full-scale trial;
- refining the screening process to screen more potentially eligible participants, even if some prove ineligible afterwards, with advanced information about site patient databases;
- reviewing the secondary outcome measures;
- redesigning some aspects of the health resource use questionnaire; and
- approaching other prosthetics centres nationally to explore their willingness to be involved in a full-scale trial.

CONCLUSION

Although we did not reach our target sample, due to slower than anticipated recruitment, 71% of expected participants were in the final analysis, owing to our retention rate of 93%. Therefore, we believe the STEPFORWARD study demonstrated feasibility to recruit and retain participants and the ability to obtain a variety of complete datasets collected in clinic and via post. We have also evidenced the ability to deliver the trial across multiple prosthetics centres in England. The knowledge gained and lessons learned can be implemented in a future definitive trial to determine the effectiveness and cost-effectiveness of a self-aligning prosthetic ankle-foot compared to a non-self-aligning version for people with a transtibial amputation and categorised as having 'limited community mobility'.

FIGURE CAPTION

Figure 1: CONSORT flowchart for the STEPFORWARD study

For peer review only - http://bmjopen.bmj.com/site/about/guidelines.xhtml

REFERENCES

1. University of Salford. LIMBLESS STATISTICS: Repository for quantitative information on the UK limbless population REFERRED for prosthetics treatment, Annual report 2011-2012. University of Salford; 2013.

2. Davies M, Burdett L, Bowling F, Ahmad N, McClennon J. The epidemiology of major lower-limb amputation in England: a systematic review highlighting methodological differences of reported trials. Diabet Foot J. 2019;22:53-61.

3. Crawford F, Cezard G, Chappell FM, Murray GD, Price JF, Sheikh A, et al. A systematic review and individual patient data meta-analysis of prognostic factors for foot ulceration in people with diabetes: the international research collaboration for the prediction of diabetic foot ulcerations (PODUS). Health Technol Assess. 2015;19(57).

4. Sansam K, O'Connor RJ, Neumann V, Bhakta B. Clinicians' perspectives on decision making in lower limb amputee rehabilitation. J Rehabil Med. 2014;46(5):447-53.

5. Hofstad C, Linde H, Limbeek J, Postema K. Prescription of prosthetic ankle-foot mechanisms after lower limb amputation. Cochrane Database Syst Rev. 2004(1):CD003978.

6. Askew GN, McFarlane LA, Minetti AE, Buckley JG. Energy cost of ambulation in trans-tibial amputees using a dynamic-response foot with hydraulic versus rigid 'ankle': insights from body centre of mass dynamics. J Neuroeng Rehabil. 2019;16(39).

7. Vrieling AH, van Keeken HG, Schoppen T, Otten E, Halbertsma JPK, Hof AL, et al. Uphill and downhill walking in unilateral lower limb amputees. Gait Posture. 2008; 28(2):235-242.

8. Agrawal V, Gailey RS, Gaunaurd IA, O'Toole C, Finnieston A, Tolchin R. Comparison of four different categories of prosthetic feet during ramp ambulation in unilateral transtibial amputees. Prosthet Orthot Int. 2015;39(5):380-9.

9. Johnson L, De Asha AR, Munjal R, Kulkarni J, Buckley JG. Toe clearance when walking in people with unilateral transtibial amputation: Effects of passive hydraulic ankle. J Rehabil Res Dev. 2014;51(3):429-37.

10. De Asha AR, Johnson L, Munjal R, Kulkarni J, Buckley JG. Attenuation of centre-of-pressure trajectory fluctuations under the prosthetic foot when using an articulating hydraulic ankle attachment compared to fixed attachment. Clin Biomech. 2013;28(2):218-24.

11. Barnett CT, Brown OH, Bisele MM, Brown MJ, De Asha AR, Strutzenberger G. Individuals with unilateral transtibial amputation and lower activity levels walk more quickly when using a hydraulically articulating versus rigidly attached prosthetic ankle-foot device. J Prosthet Orthot. 2018;30(3):158-64.

12. Moore R. Effect on stance phase timing asymmetry in individuals with amputation using hydraulic ankle units. J Prosthet Orthot. 2016;28:44-8.

13. Moore R. Patient evaluation of a novel prosthetic foot with hydraulic ankle aimed at persons with amputation with lower activity levels. J Prosthet Orthot. 2017;29:44-7.

For peer review only - http://bmjopen.bmj.com/site/about/guidelines.xhtml

14. Goujon H, Bonnet X, Sautreuil P, Maurisset M, Darmon L, Fode P, et al. A functional evaluation of prosthetic foot kinematics during lower-limb amputee gait. Prosthet Orthot Int. 2006;30(2):213-23.

15. Eldridge SM, Lancaster GA, Campbell MJ, Thabane L, Hopewell S, Coleman CL, et al. Defining feasibility and pilot studies in preparation for randomised controlled trials: Development of a conceptual framework. PLoS ONE. 2016;11(3):e0150205.

16. Mitchell N, Coleman E, Watson J, Bell K, McDaid C, Barnett C, et al. Self-aligning prosthetic device for older patients with vascular-related amputations: protocol for a randomised feasibility study (the STEPFORWARD study). BMJ Open. 2019;9(9):e032924.

17. Franchignoni F, Orlandini D, Ferriero G, Moscato TA. Reliability, validity, and responsiveness of the locomotor capabilities index in adults with lower-limb amputation undergoing prosthetic training. Arch Phys Med Rehabil. 2004;85(5):743-8.

18. Devlin M, Pauley T, Head K, Garfinkel S. Houghton Scale of prosthetic use in people with lower extremity amputations: Reliability, validity, and responsiveness to change. Arch Phys Med Rehabil. 2004;85(8):1339-44.

19. Morgan SJ, Friedly JL, Amtmann D, Salem R, Hafner BJ. Cross-sectional assessment of factors related to pain ontensity and pain interference in lower limb prosthesis users. Arch Phys Med Rehabil. 2017;98(1):105-13.

20. Hurst NP, Kind P, Ruta D, Hunter M, Stubbings A. Measuring health-related quality of life in rheumatoid arthritis: Validity, responsiveness and reliability of EuroQol (EQ-5D). Br J Rheumatol. 1997;36(5):551-9.

21. Podsiadlo D, Richardson S. The timed "Up & Go": A test of basic functional mobility for frail elderly persons. J Am Geriatr Soc. 1991;39(2):142-8.

22. Bonnyaud C, Zory R, Pradon D, Vuillerme N, Roche N. Clinical and biomechanical factors which predict timed up and down stairs test performance in hemiparetic patients. Gait Posture. 2013;38(3):466-70.

23. Butland RJA, Pang J, Gross ER, Woodcock AA, Geddes DM. Two- ,six-, and 12-minute walking tests in respiratory-disease. Br Med J. 1982;284(6329):1607-8.

24. Major MJ, Fatone S, Roth EJ. Validity and reliability of the Berg Balance Scale for community-dwelling persons with lower-limb amputation. Arch Phys Med Rehabil. 2013;94(11):2194-202.

25. Department of Health. NHS reference costs 2017/18. London. 2018 [Available from: https://improvement.nhs.uk/resources/reference-costs/#rc1718.

26. Curtis L, Burns A. Unit Costs of Health and Social Care 2016. Personal Social Services Research Unit: University of Kent; 2016.

27. Kim J, Major MJ, Hafner B, Sawers A. Frequency and circumstances of falls reported by ambulatory unilateral lower limb prosthesis users: A secondary analysis. PM R. 2019;11(4):344-53.

For peer review only - http://bmjopen.bmj.com/site/about/guidelines.xhtml

28. Miller WC, Speechley M, Deathe B. The prevalence and risk factors of falling and fear of falling among lower extremity amputees. Arch Phys Med Rehabil. 2001;82(8):1031-7.

29. Gailey RS, Gaunaurd I, Agrawal V, Finnieston A, O'Toole C, Tolchin R. Application of self-report and performance-based outcome measures to determine functional differences between four categories of prosthetic feet. J Rehabil Res Dev. 2012;49(4):597-612.

30. Morgan SJ, McDonald CL, Halsne EG, Cheever SM, Salem R, Kramer PA, Hafner BJ. Laboratory- and community-based health outcomes in people with transtibial amputation using crossover and energy-storing prosthetic feet: A randomized crossover trial. PLoS ONE. 2018;13(2): e0189652.

31. Raschke SU, Orendurff MS, Mattie JL, Kenyon DEA, Jones OY, Moe D, Winder L, Wong AS, Moreno-Hernandez A, Highsmith MJ, Sanderson DJ, Kobayashi T. Biomechanical characteristics, patient preference and activity level with different prosthetic feet: A randomized double blind trial with laboratory and community testing. J Biomech. 2015;48(1):146-52.

32. Wood L, Egger M, Gluud LL, Schulz KE, Juni P, Altman DG, Gluud C, Martin RM, Wood AJG, Sterne JAC. Empirical evidence of bias in treatment effect estimates in controlled trials with different interventions and outcomes: meta-epidemiological study. Br Med J. 2008;336(7644):601-5.

33. Miller MJ, Cook PF, Kline PW, Anderson CB, Stevens-Lapsley JE, Christiansen CL. Physical function and pre-amputation characteristics explain daily step count after dysvascular amputation. PM R. 2019;11(10):1050-8.

For peer review only

For peer review only - http://bmjopen.bmj.com/site/about/guidelines.xhtml

Table 1: Demographic characteristics according to trial arm

	Self-aligning (n=27)	Standard (n=28)	Overall (n=55)
**Age, years**	N=27	N=28	N=55
Mean (SD)	70.0 (9.2)	67.6 (10.0)	68.8 (9.6)
Median (min, max)	71.5 (52.7, 86.8)	68.3 (51.8, 84.5)	70.7 (51.8, 86.8)
**Gender, n(%)**			
Male	23 (85.2)	24 (85.7)	47 (85.5)
Female	4 (14.8)	4 (14.3)	8 (14.6)
**Ethnicity, n(%)**			
White (British, Irish or Other)	27 (100.0)	28 (100.0)	55 (100.0)
Black (African, Caribbean or Other)	0 (0.0)	0 (0.0)	0 (0.0)
Asian (Indian, Pakistani, Bangladeshi or Other)	0 (0.0)	0 (0.0)	0 (0.0)
Mixed Background	0 (0.0)	0 (0.0)	0 (0.0)
Chinese	0 (0.0)	0 (0.0)	0 (0.0)
**Marital status, n(%)**			
Living alone never married	2 (7.4)	3 (10.7)	5 (9.1)
Living with partner	4 (14.8)	0 (0.0)	4 (7.3)
Married/civil partnership	9 (33.3)	19 (67.9)	28 (50.9)
Separated	2 (7.4)	1 (3.6)	3 (5.5)
Divorced	5 (18.5)	4 (14.3)	9 (16.4)
Widowed	2 (7.4)	1 (3.6)	3 (5.5)
Missing	3 (11.1)	0 (0.0)	3 (5.5)
**Main activity, n(%)**			
Full-time employment	1 (3.7)	3 (10.7)	4 (7.3)
Part-time employment	0 (0.0)	1 (3.6)	1 (1.8)
Self-employed	0 (0.0)	0 (0.0)	0 (0.0)
Unable to work due to poor health	1 (3.7)	5 (17.9)	6 (10.9)
Unemployed	0 (0.0)	1 (3.6)	1 (1.8)
Retired	24 (88.9)	17 (60.7)	41 (74.6)
Other	1 (3.7)	1 (3.6)	2 (3.6)
**Smoking status, n(%)**			
Ex-smoker	15 (55.6)	17 (60.7)	32 (58.2)
Current smoker	1 (3.7)	3 (10.7)	4 (7.3)
Never smoked	11 (40.7)	8 (28.6)	19 (34.6)
**Diabetes status, n(%)**			
Yes	19 (70.4)	18 (64.3)	37 (67.3)
No	8 (29.6)	10 (35.7)	18 (32.7)
**If yes:**			
Type one	5 (26.3)	2 (11.1)	7 (18.9)
Type two	12 (63.2)	13 (72.2)	25 (67.8)
Missing	2 (10.5)	3 (16.7)	5 (13.5)
**Cause of amputation, n(%)[a]**			
Diabetes	14 (51.9)	11 (39.3)	25 (45.5)
Peripheral vascular disease	10 (37.0)	10 (35.7)	20 (36.4)
Blood clot	3 (11.1)	2 (7.1)	5 (9.1)

For peer review only - http://bmjopen.bmj.com/site/about/guidelines.xhtml

Deep vein thrombosis	1 (3.7)	0 (0.0)	1 (1.8)
Aneurysm	0 (0.0)	1 (3.6)	1 (1.8)
Diabetic neuropathy	2 (7.4)	1 (3.6)	3 (5.5)
Other neuropathy	1 (3.7)	0 (0.0)	1 (1.8)
Cancer	1 (3.7)	1 (3.6)	2 (3.6)
Other	5 (18.5)	6 (21.4)	11 (20.0)

[a] Multiple options could be selected

For peer review only

For peer review only - http://bmjopen.bmj.com/site/about/guidelines.xhtml

Table 2. Standard outcomes at each time point (baseline, interim and final follow-up), by trial arm and overall

	Self-aligning (n=27)	Standard (n=28)	Overall (n=55)
**LCI-5 overall score (0 – 56): average completion 94%**			
**Baseline**	N=25	N=28	N=53
Mean (SD)	36.4 (12.0)	39.6 (11.2)	39.1 (11.6)
Median (p25, p75)	38 (30.3, 46)	38 (31, 50)	38 (30.3, 47)
**Interim**	N=24	N=25	N=49
Mean (SD)	37.3 (12.3)	38.5 (10.8)	37.9 (11.4)
Median (p25, p75)	39.5 (28.5, 47.5)	41 (30, 44.7)	41 (29, 46)
**Final**	N=25	N=26	N=51
Mean (SD)	37.8 (11.4)	39.2 (11.2)	38.5 (11.2)
Median (p25, p75)	40 (30, 47)	40.5 (29, 49)	40 (29, 47)
**Houghton overall score (0 – 12): average completion: 89%**			
**Baseline**	N=24	N=28	N=52
Mean (SD)	7.8 (2.0)	8.8 (1.8)	8.3 (1.9)
Median (p25, p75)	8 (7.5, 9)	9 (8, 10)	9 (8, 9)
**Interim**	N=20	N=23	N=43
Mean (SD)	8.4 (2.0)	8.5 (1.3)	8.5 (1.6)
Median (p25, p75)	9 (7, 10)	9 (8, 9)	9 (8, 10)
**Final**	N=23	N=26	N=49
Mean (SD)	8.3 (1.9)	8.5 (2.0)	8.4 (1.9)
Median (p25, p75)	8 (8, 10)	9 (8, 9)	9 (8, 9)
**PROMIS 3a overall score (30.7 – 71.8): average completion: 90%**			
**Baseline**	N=27	N=27	N=54
Mean (SD)	41.3 (10.0)	43.1 (9.5)	42.2 (9.7)
Median (p25, p75)	43.5 (30.7, 52.1)	43.5 (30.7, 49.4)	43.5 (30.7, 49.4)
**Interim**	N=21	N=25	N=46
Mean (SD)	43.6 (9.6)	45.6 (10.3)	44.7 (10.0)
Median (p25, p75)	43.5 (30.7, 49.4)	46.3 (40.2, 52.1)	46.3 (30.7, 52.1)
**Final**	N=24	N=22	N=46
Mean (SD)	40.4 (9.4)	42.7 (8.7)	41.4 (9.0)
Median (p25, p75)	40.2 (30.7, 47.9)	44.9 (30.7, 49.4)	43.5 (30.7, 49.4)
**PROMIS 8a overall score (40.7 – 77.0): average completion: 94%**			
**Baseline**	N=27	N=28	N=55
Mean (SD)	52.8 (11.2)	51.7 (9.4)	52.2 (10.2)
Median (p25, p75)	55 (40.7, 63.5)	51.8 (40.7, 58.5)	52.3 (40.7, 60.2)
**Interim**	N=23	N=24	N=47
Mean (SD)	53.5 (11.3)	56.7 (8.9)	55.1 (10.1)
Median (p25, p75)	55 (40.7, 64.1)	58.8 (52.3, 63.2)	57.4 (40.7, 63.5)
**Final**	N=25	N=26	N=51
Mean (SD)	51.2 (11.4)	53.3 (10.0)	52.3 (10.7)
Median (p25, p75)	49.9 (40.7, 60.2)	54.5 (40.7, 60.2)	53.2 (40.7, 60.2)

LCI-5 (Locomotor Capabilities Index-5): higher scores indicate a greater locomotor capability with the prosthesis; Houghton: higher scores indicate greater performance and comfort; PROMIS (Patient-Reported Outcomes Measurement Information System) 3a (pain intensity): coverts raw scores to T-scores, higher scores indicate more intense pain; PROMIS 8a: higher scores indicate more pain interference.

For peer review only - http://bmjopen.bmj.com/site/about/guidelines.xhtml

Table 3. Clinical assessments (TUG, TUDS, 2mWT and BBS) at baseline and final follow-up, by trial arm and overall

	Self-aligning (n=27)	Standard (n=28)	Overall (n=55)
**TUG, seconds: average completion 95%**			
**Baseline**	N=27	N=28	N=55
Mean (SD)	20.8 (15.2)	21.3 (23.5)	21.1 (19.7)
Median (p25, p75)	17 (12, 24)	14.5 (11.5, 23)	17 (12, 23)
Unable to complete	0	0	0
**Final**	N=23	N=22	N=45
Mean (SD)	21.0 (19.1)	17.8 (8.4)	19.5 (14.8)
Median (p25, p75)	16 (11, 13)	14.5 (13, 22)	15 (11, 23)
Unable to complete	0	2	0
**TUDS, seconds: average completion 93%**			
**Baseline**	N=17	N=27	N=44
Mean (SD)	61.4 (33.0)	66.9 (38.5)	64.7 (36.2)
Median (p25, p75)	50 (39, 72)	51 (41, 88)	50.5 (41, 88)
Unable to complete	9	1	10
**Final**	N=19	N=21	N=40
Mean (SD)	59.2 (29.2)	68.1 (56.7)	63.9 (45.4)
Median (p25, p75)	51 (33, 79)	48 (39, 74)	48.5 (38, 76.5)
Unable to complete:	4	3	7
**2mWT, metres: average completion 95%**			
**Baseline**	N=27	N=28	N=55
Mean (SD)	81.0 (31.4)	94.7 (33.5)	87.9 (33.5)
Median (p25, p75)	77 (60, 110)	95 (68, 113)	83 (61, 113)
**Final**	N=23	N=24	N=47
Mean (SD)	88.8 (40.0)	83.9 (41.5)	86.3 (39.5)
Median (p25, p75)	80 (62, 120)	80 (57.5, 106)	80 (60, 108)
**BBS, scored 0-56: average completion 92%**			
**Baseline**	N=26	N=27	N=53
Mean (SD)	39.5 (11.3)	42.9 (8.1)	41.3 (9.8)
Median (p25, p75)	41.5 (32, 48)	43 (36, 51)	43 (34, 48)
**Final**	N=22	N=24	N=46
Mean (SD)	40.7 (12.9)	39.4 (11.6)	40.0 (12.1)
Median (p25, p75)	44 (30, 51)	41.5 (33, 49)	41.5 (33, 50)
**Activity monitor: minutes stepping per day**			
**Baseline**	N=26	N=27	N=53
Mean (SD)	29.9 (30.8)	31.1 (24.9)	30.5 (27.7)
Median (p25, p75)	24.7 (9.4, 31.4)	26 (14.5, 40.3)	25.9 (10.6, 34.1)
**Final**	N=22	N=25	N=47
Mean (SD)	27.7 (24.0)	31.2 (25.8)	29.6 (24.8)
Median (p25, p75)	24.1 (3.6, 45.0)	25.9 (17.3, 31.1)	25.7 (10.6, 40.3)
**Activity monitor: steps taken per day**			
**Baseline**	N=26	N=27	N=53
Mean (SD)	1755.5 (1963.7)	1750.9 (1369.6)	1753.2 (1670.9)
Median (p25, p75)	1292.3 (587.1, 1835.7)	1572.9 (802.3, 2234.6)	1448.6 (654.6, 2144.0)
**Final**	N=22	N=25	N=47

For peer review only - http://bmjopen.bmj.com/site/about/guidelines.xhtml

Mean (SD)	1673.2 (1594.4)	1836.1 (1647.8)	1759.9 (1607.4)
Median (p25, p75)	1356.4 (204.0, 2719.4)	1510.3 (922.9, 1908.0)	1446.3 (551.4, 2270.0)

TUG (Timed Up and Go test) and TUDS (Timed Up and Down Stair test): faster time indicates better performance; 2mWT (2-minute walk test): further distance indicates better walking ability; BBS (Berg Balance Scale): higher score indicates better balance.

For peer review only

For peer review only - http://bmjopen.bmj.com/site/about/guidelines.xhtml

Table 4: EQ-5D-5L mean (SD) utility scores, unadjusted mean difference (95% CI) and adjusted for baseline utility at each time point (baseline, interim and final follow-up), by trial arm

	Self-aligning (n=27)	Standard (n=28)	Unadjusted difference (self-aligning vs. standard) (95% CI)	Adjusted difference (self-aligning vs. standard) (95% CI)
**EQ-5D-5L (average completion 93%)**				
**Baseline**	0.62 (0.30)	0.63 (0.35)	-0.009 (-0.188, 0.169)	-0.009 (-0.188, 0.169)
**Interim**	0.64 (0.18)	0.57 (0.24)	0.067 (-0.055, 0.189)	0.104 (0.001, 0.207)
**Final**	0.75 (0.16)	0.63 (0.32)	0.120 (-0.021, 0.262)	0.117 (0.017, 0.216)

For peer review only

For peer review only - http://bmjopen.bmj.com/site/about/guidelines.xhtml

Twitter
Follow Natalie Vanicek @NatalieVanicek and Cleveland Barnett @Cleve_Barnett

Acknowledgements
This work uses data provided by patients and collected by researchers and members of the NHS who worked collaboratively on the STEPFORWARD trial. The collection of data would not have been possible without the support of the participating NHS Trusts and their staff. The authors would also like to thank the members of the Project Advisory Group (PAG): Dennis Harrison, Peter Wignall, Anthony Gick and Kevin Waller for their invaluable advice on many aspects of the project.

Author contributions
NV led on the conception, design and writing of this manuscript. NM, EC, JW, KB, CMcD, CB, MT, FJ, AS and DH contributed to the design, writing, critical review of intellectual content and final manuscript approval. All authors agree to be accountable for their work. As Chief Investigator, NV takes overall responsibility for the work. EC provided statistical expertise in the study design and development stages of the project and the protocol. NM, JW, and CMcD made substantial contributions to the trial design and management. DH provided particular input to PPI. KB was specifically responsible for the health economic aspects of the study design and CB, MT, FJ and AS were responsible for providing expert clinical support.

Funding statement
This paper presents independent research funded by the National Institute for Health Research (NIHR) under its Research for Patient Benefit (RfPB) Programme (Grant Reference Number PB-PG-0816-20029). Hull University Teaching Hospitals NHS Trust are the sponsors of the study, on behalf of the funder.

Blatchford (Basingstoke, UK) provided the prosthetic feet examined in this research at no cost. The prosthetic feet are currently commercially available devices. Blatchford have had no part in the study design nor are they be involved directly in the research, its subsequent analysis and ultimate dissemination.

Disclaimer
The views expressed are those of the author(s) and not necessarily those of the NHS, the NIHR, the Department of Health and Social Care, or Blatchford.

Patient and public involvement
Patients and public involvement members were involved in the design, conduct, reporting and dissemination plans of this research.

Patient consent for publication
Not required

Competing interests
None declared.

For peer review only

Ethics approval

Ethical approval by Yorkshire & The Humber – Leeds West Research Ethics Committee (IRAS project ID 232950; REC reference 18/YH/0089) and HRA approval were obtained on 4th May 2018.

Data availability statement

The data that support the findings of this study are available from the corresponding author, [NV], upon reasonable request.

For peer review only

For peer review only - http://bmjopen.bmj.com/site/about/guidelines.xhtml

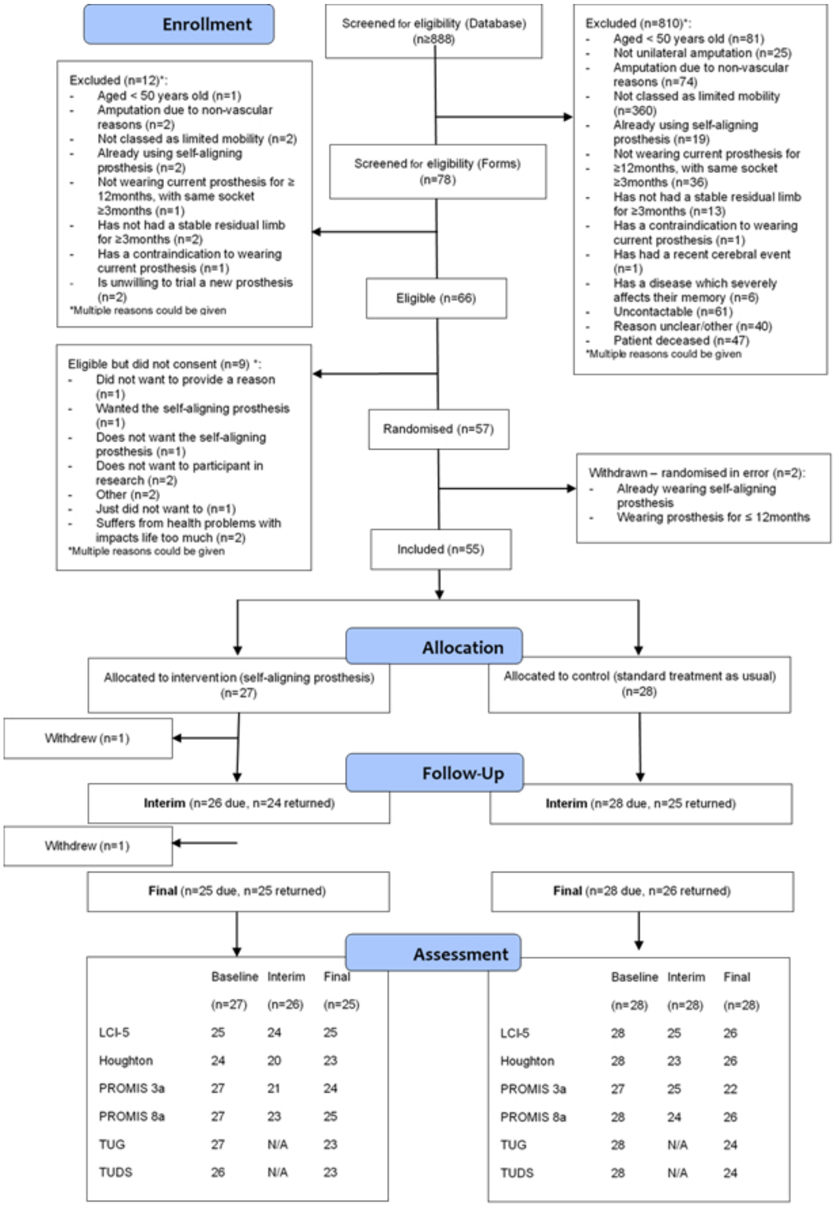

Figure 1: CONSORT flowchart for the STEPFORWARD study

161x233mm (96 x 96 DPI)

CONSORT 2010 checklist of information to include when reporting a pilot or feasibility trial*

Section/Topic	Item No	Checklist item	Reported on page No
**Title and abstract**			
	1a	Identification as a pilot or feasibility randomised trial in the title	1
	1b	Structured summary of pilot trial design, methods, results, and conclusions (for specific guidance see CONSORT abstract extension for pilot trials)	2
**Introduction**			
Background and objectives	2a	Scientific background and explanation of rationale for future definitive trial, and reasons for randomised pilot trial	4-5
	2b	Specific objectives or research questions for pilot trial	5
**Methods**			
Trial design	3a	Description of pilot trial design (such as parallel, factorial) including allocation ratio	5
	3b	Important changes to methods after pilot trial commencement (such as eligibility criteria), with reasons	n/a
Participants	4a	Eligibility criteria for participants	5-6
	4b	Settings and locations where the data were collected	6
	4c	How participants were identified and consented	6
Interventions	5	The interventions for each group with sufficient details to allow replication, including how and when they were actually administered	6
Outcomes	6a	Completely defined prespecified assessments or measurements to address each pilot trial objective specified in 2b, including how and when they were assessed	7
	6b	Any changes to pilot trial assessments or measurements after the pilot trial commenced, with reasons	n/a
	6c	If applicable, prespecified criteria used to judge whether, or how, to proceed with future definitive trial	n/a
Sample size	7a	Rationale for numbers in the pilot trial	6
	7b	When applicable, explanation of any interim analyses and stopping guidelines	n/a
Randomisation:			
Sequence generation	8a	Method used to generate the random allocation sequence	n/a
	8b	Type of randomisation(s); details of any restriction (such as blocking and block size)	6
Allocation concealment mechanism	9	Mechanism used to implement the random allocation sequence (such as sequentially numbered containers), describing any steps taken to conceal the sequence until interventions were assigned	n/a

Implementation	10	Who generated the random allocation sequence, who enrolled participants, and who assigned participants to interventions	6
Blinding	11a	If done, who was blinded after assignment to interventions (for example, participants, care providers, those assessing outcomes) and how	n/a
	11b	If relevant, description of the similarity of interventions	n/a
Statistical methods	12	Methods used to address each pilot trial objective whether qualitative or quantitative	7
**Results**			
Participant flow (a diagram is strongly recommended)	13a	For each group, the numbers of participants who were approached and/or assessed for eligibility, randomly assigned, received intended treatment, and were assessed for each objective	8, Figure 1
	13b	For each group, losses and exclusions after randomisation, together with reasons	8, Figure 1
Recruitment	14a	Dates defining the periods of recruitment and follow-up	6
	14b	Why the pilot trial ended or was stopped	n/a
Baseline data	15	A table showing baseline demographic and clinical characteristics for each group	Table 1
Numbers analysed	16	For each objective, number of participants (denominator) included in each analysis. If relevant, these numbers should be by randomised group	Tables 1-4
Outcomes and estimation	17	For each objective, results including expressions of uncertainty (such as 95% confidence interval) for any estimates. If relevant, these results should be by randomised group	Tables 1-4
Ancillary analyses	18	Results of any other analyses performed that could be used to inform the future definitive trial	n/a
Harms	19	All important harms or unintended effects in each group (for specific guidance see CONSORT for harms)	8
	19a	If relevant, other important unintended consequences	n/a
**Discussion**			
Limitations	20	Pilot trial limitations, addressing sources of potential bias and remaining uncertainty about feasibility	13
Generalisability	21	Generalisability (applicability) of pilot trial methods and findings to future definitive trial and other studies	10-13
Interpretation	22	Interpretation consistent with pilot trial objectives and findings, balancing potential benefits and harms, and considering other relevant evidence	10-13
	22a	Implications for progression from pilot to future definitive trial, including any proposed amendments	14
**Other information**			
Registration	23	Registration number for pilot trial and name of trial registry	2
Protocol	24	Where the pilot trial protocol can be accessed, if available	5-6
Funding	25	Sources of funding and other support (such as supply of drugs), role of funders	24
	26	Ethical approval or approval by research review committee, confirmed with reference number	25

For peer review only

Citation: Eldridge SM, Chan CL, Campbell MJ, Bond CM, Hopewell S, Thabane L, et al. CONSORT 2010 statement: extension to randomised pilot and feasibility trials. BMJ. 2016;355.
*We strongly recommend reading this statement in conjunction with the CONSORT 2010, extension to randomised pilot and feasibility trials, Explanation and Elaboration for important clarifications on all the items. If relevant, we also recommend reading CONSORT extensions for cluster randomised trials, non-inferiority and equivalence trials, non-pharmacological treatments, herbal interventions, and pragmatic trials. Additional extensions are forthcoming: for those and for up to date references relevant to this checklist, see www.consort-statement.org.

For peer review only
